# Soil Metabarcoding Offers a New Tool for the Investigation and Hunting of Truffles in Northern Thailand

**DOI:** 10.3390/jof7040293

**Published:** 2021-04-13

**Authors:** Nakarin Suwannarach, Jaturong Kumla, Ammarin In-on, Saisamorn Lumyong

**Affiliations:** 1 Research Center of Microbial Diversity and Sustainable Utilization, Chiang Mai University, Chiang Mai 50200, Thailand; Jaturong_yai@hotmail.com (J.K.); scboi009@gmail.com (S.L.); 2 Department of Biology, Faculty of Science, Chiang Mai University, Chiang Mai 50200, Thailand; 3 Bioinformatic and Systems Biology Program, King Mongkut’s University of Technology Thonburi, Bang Kun Thian, Bangkok 10150, Thailand; ammarin.ammarinin@mail.kmutt.ac.th; 4 Academy of Science, The Royal Society of Thailand, Bangkok 10300, Thailand

**Keywords:** environmental DNA, hypogeous fungi, next-generation sequencing, soil fungal community, *Tuber*

## Abstract

Truffles (*Tuber* spp.) are well-known as edible ectomycorrhizal mushrooms, and some species are one of the most expensive foods in the world. During the fruiting process, truffles produce hypogeous ascocarps; a trained pig or dog is needed to locate the ascocarps under the ground. Truffles in northern Thailand have been recorded in association with *Betula* *alnoides* and *Carpinus poilanei*. In this study, we investigated the soil mycobiota diversity of soil samples from both of these truffle host plants in native forests using environmental DNA metabarcoding to target the internal transcribed spacer 1 (ITS1) region of the rDNA gene for the purposes of investigation of truffle diversity and locating truffles during the non-fruiting phase. In this study, a total of 38 soil samples were collected from different locations. Of these, truffles had been found at three of these locations. Subsequently, a total of 1341 putative taxonomic units (OTUs) were obtained. The overall fungal community was dominated by phylum-level sequences assigned to Ascomycota (57.63%), Basidiomycota (37.26%), Blastocladiomycota (0.007%), Chytridiomycota (0.21%), Glomeromycota (0.01%), Kickxellomycota (0.01%), Mortierellomycota (2.08%), Mucoromycota (0.24%), Rozellomycota (0.01%), Zoopagomycota (0.003%), and unidentified (2.54%). The results revealed that six OTUs were determined to be representative and belonged to the genus *Tuber*. OTU162, OTU187, OTU447, and OTU530 belonged to *T*. *thailandicum*, *T*. *lannaense*, *T. bomiense*, and *T*. *magnatum*, whereas OTU105 and OTU720 were acknowledged as unrecognized *Tuber* species. From 38 locations, OTUs of truffles were found in 33 locations (including three previously known truffle locations). Thus, 30 collection sites were considered new locations for *T*. *thailandicum*, *T. bomiense*, and other unrecognized *Tuber* species. Interestingly, at 16 new locations, mature ascocarps of truffles that were undergoing the fruiting phase were located underground. All 16 truffle samples were identified as *T. thailandicum* based on morphological characteristics and molecular phylogenetic analysis. However, ascocarps of other truffle species were not found at the new OTUs representative locations. The knowledge gained from this study can be used to lead researchers to a better understanding of the occurrence of truffles using soil mycobiota diversity investigation. The outcomes of this study will be particularly beneficial with respect to the search and hunt for truffles without the need for trained animals. In addition, the findings of this study will be useful for the management and conservation of truffle habitats in northern Thailand.

## 1. Introduction

Truffles are one of the most expensive edible mushrooms in the world and belong to the genus *Tuber* within the family *Tuberaceae*, order Pezizales [1,2]. Generally, the life cycle of a truffle takes place underground by ectomycorrhizal symbiosis, where they produce hypogeous ascocarps [2,3,4]. Ascocarps of truffles are only formed during the fruiting phase. The mature state of ascocarps of truffles leads to the release of volatile compounds that are commonly detected by insects and mammals who consume these ascocarps. This process ultimately results in the dispersal of the truffle’s spores throughout the environment [5,6]. However, it can be difficult for humans to find truffles in native forests. Traditionally, the process of searching for and collecting truffles has required the use of female pigs and trained dogs. With their advanced sense of smell, these animals are able to detect the distinct odor of mature truffles underneath the surface of the soil. Therefore, the involvement of trained animals has always been considered a crucial element in the search for truffles in their natural habitat [2,4,6]. Truffles mostly grow in forests in temperate zones throughout Asia, Europe, North Africa, and North America. Generally, woody trees in five families, namely *Betulaceae*, *Cistaceae*, *Fagaceae*, and *Pinaceae*, are known to form ectomycorrhizal relationships with most truffle species [2,4,7]. The most popular truffles in the world are the Italian white truffle (*T*. *magnatum*), the garlic truffle (*T*. *macrosporum*), the Périgord black truffle (*T*. *melanosporum*), the summer truffle (*T*. *aestivum*), and the Bianchetto white truffle (*T. borchii*). All of these truffle species are predominantly found in Europe [2,4,8,9]. In America, the Oregon spring white truffle (*T*. *gibbosum*) and the Oregon winter white truffle (*T*. *oregonense*) are commercially harvested in the northern region of the state [10], while the pecan truffle (*T. lyonii*) is harvested in the southern region [11]. Additionally, the Chinese black truffle (*T*. *indicum*) is the most famous commercial truffle in Asia [12]. Interestingly, Asian truffles have been harvested for many decades, and more than 25 new *Tuber* species have been discovered in Asia since the 1980s, especially in China, Japan, and Taiwan [13,14,15,16,17,18,19,20]. Previous studies have reported that there is a high degree of diversity of *Tuber* in Asia but this has still not been fully investigated [17,20]. Thailand is proving to be a hotspot for the discovery of a range of novel fungal species [21]. Evidence of this would include the discovery of two new *Tuber* species (*T*. *lannaense* and *T*. *thailandicum*) and the Italian white truffle in Southeast Asia in just the last five years [22,23,24]. Importantly, these three *Tuber* species have been grown in mycorrhizal association with the plant family *Betulaceae*, including *Betula alnoides* and *Carpinus poilanei* [22,23,24].

Environmental DNA (eDNA) revealed that the genetic material presented in the environmental samples, e.g., air, soil, sediment, and water, included both extracellular and intracellular DNA [25,26,27]. Notably, eDNA has been employed in a range of fundamental research studies in the fields of molecular biology, ecology, environmental science, and paleontology through high-throughput next-generation sequencing [26,27,28]. Recently, eDNA metabarcoding has become an important research tool and is now being commonly used to understand the microbial (including bacteria and fungi) diversity and community structure in a variety of environments [29,30,31,32]. Thus, soil mycobiota diversity has been provided to establish the ecological groups of fungi as mycorrhizal (mutualistic symbiotic association of fungi and plants), saprotrophic (decomposers), and pathogenic (harmful or parasitic to other organisms) [33,34,35,36]. Several previous studies have been conducted to evaluate and monitor soil fungal diversity and fungal communities, as well as to detect rare fungal species in soil samples collected from various forest types during different seasons with the use of eDNA [36,37,38]. In previous decades, species-specific primers for polymerase chain reactions (PCR) were used to detect truffles in the environment [39,40,41]; however, a notable disadvantage of this method was that it requires a large amount of extracted DNA to be present in the samples. Consequently, a number of unexpected truffle species were missed [42,43]. Due to the fact that trained animals have always been critical to the process of truffle hunting, truffle collection in Thailand has been limited. In this study, the soil mycobiota diversity of truffle host plants (*B. alnoides* and *C. poilanei*) in northern Thailand were investigated using eDNA metabarcoding (ITS1 region of rDNA). This was done for the purposes of developing the capability of investigating truffle diversity and locating truffles in their natural habitats during the non-fruiting phase (the dry season). The ITS sequence (ITS1 and ITS2 regions) has been used for the fungal metabarcoding [44,45]. However, the ITS1 region has been widely used as a universal fungal barcode for the quick and general analyses of diversity and ITS1 outperforms ITS2 in terms of richness and taxonomic coverage [46,47,48]. Thus, we selected the ITS1 region for this study. According to the information gathered from the DNA of truffles in soil samples taken during the wet to cold seasons, the hunt for truffle ascocarps was conducted in those seasons. Furthermore, the hunting for ascocarps of truffles was conducted during wet to cold seasons. The collected truffle ascocarps were identified by their morphological characteristics combined with a phylogenetic analysis. The results of this study will provide valuable information to researchers and truffle hunters and enable them to gain a better understanding of the distribution of truffles using soil mycobiota diversity investigation. The results could also be used to further develop relevant strategies for indicating truffle locations and searching truffles without trained pigs or dogs.

## 2. Materials and Methods

### 2.1. Ethics Statement

The collection of soil samples was permitted by the Department of National Parks, Wildlife and Plant Conservation, Bangkok, Thailand under document number 0907.4/4769.

### 2.2. Soil Sampling and eDNA Extraction

**Figure 1:**
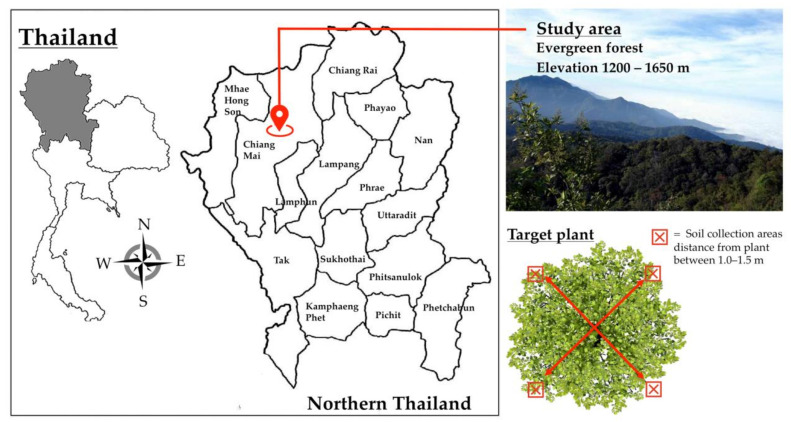
Map showing the study area and position of soil sample collection of each target plant.

This study was conducted in the native evergreen forests (elevation 1200–1650 m) of Doi Suthep Mountain, Mueang Chiang Mai, Chiang Mai Province, northern Thailand (Figure 1). The study site was located in nature reserves within Doi Suthep-Pui National Park. The dominant tree genera in the study site were *Castanopsis, Quercus*, and *Pinus*. Notably, *B. alnoides* (*n* = 36) and *C. poilanei* (*n* = 2) were found within the study site (Supplementary Table S1). Thus, this study focused on the soil samples of *B. alnoides* and *C. poilanei* as host plants of truffles in northern Thailand [22,23,24]. Soil samples (*n* = 38) were collected from each host plant during the period of February to March, 2017 (the dry season and non-fruiting phase of the truffle) (Figure 1). Soil samples were randomly collected from four positions around target plants (Figure 1). After removing leaf litter, soil samples were aseptically collected at a depth of 5–10 cm from the surface of the soil and at a distance of 1.0–1.5 m from target plants using a soil core sampling tool (8.0 cm in diameter). Soil samples obtained from four positions of each target plant were pooled and kept in well-sealed sterile plastic bags. The samples were stored in an ice box and ultimately transported to the laboratory. All soil samples were air-dried at room temperature (27 ± 2 °C) immediately after reaching the laboratory. Each dried soil sample was ground using a mortar and pestle. The samples were then passed through a 250-µm sieve. All samples were stored at −80 °C until the process of eDNA extraction could be performed. Next, eDNA in the soil samples (each approximately 250 mg) was extracted using a NucleoSpin Soil DNA Isolation Kit (Macherey-Nagel, Germany) according to the manufacturers’ guidelines. All eDNA extracts were kept at −80 °C until further processing.

### 2.3. PCR Amplification, Amplicon Processing, and Illumina Sequencing

The eDNA extracts were used as templates for polymerase chain reactions (PCR) to amplify amplicons of the fungal ITS1 region of the rDNA gene using ITS5-1737F (5′-GGAAGTAAAAGTCGTAACAAGG-3′) and ITS2-2043R primers (5′-GCTGCGTTCT TCATCGATGC-3′) [44] that were linked to Illumina adapters. The amplification program was conducted with an initial denaturation step at 94 °C for 5 min, followed by 35 cycles of denaturation at 94 °C for 50 s annealing at 55 °C for 30 s and an extension step at 72 °C for 1 min with a final extension step at 72 °C for 5 min. PCR products were purified using a Qiagen Gel Extraction Kit (Qiagen, Hilden, Germany) and then sent to a commercial service provider (Novogene Bioinformatics Technology Co., Ltd., Beijing, China) for illumina sequencing.

### 2.4. PCR Sequence Analysis and Taxonomical Assignment

FLASH version 1.2.7 was used to assemble the forward and reverse sequences by overlapping paired-end reads [49]. The low-quality sequences (threshold of q30) were filtered out. The quality-filtering consisted of discarding reads with ambiguous sequences; sequence length ranges from 100 bp to 600 bp. Next, sequences that occurred only once (singletons) were discarded. Additionally, only the sequences belonging to the kingdom Fungi were kept for further analyses. Operational taxonomic units (OTUs) with a value greater than or equal to 97% similarity cut-off were clustered using UPARSE version 7.0.1001 [50]. The similarity threshold (≥97%) is commonly used in OTU-based analyses and has been shown to be an optimal threshold when using ITS to identify fungi [51]. The chimeric sequences were identified and removed with the UCHIME algorithm [52]. QIIME 1.7 was used for the purpose of taxonomy assignment with UNITE 7.2 fungal ITS reference training data set [53] and National Center for Biotechnology Information (NCBI) Taxonomy Database (http://www.ncbi.nlm.nih.gov/taxonomy, 25 May 2017) via the BLAST algorithm.

### 2.5. Truffle Hunting and Identification

In this study, truffle hunting was performed during the fruiting phase (the wet to cold seasons beginning from mid-May and extending to September in 2017) without the use of any trained pigs or dogs. Truffle ascocarps were searched and dug in both present and absence of truffle OTUs locations. The collected truffles were identified based on the morphological and molecular criteria. Morphological characteristics were observed for fresh specimens following the established method described in previous studies [15,22,54]. Genomic DNA of fresh specimens (10 mg) was extracted using a FavorPrep™ Tissue Genomic DNA Extraction Mini Kit (FAVORGEN, Taiwan). The ITS region was amplified with ITS4/ITS1F primers, under the following thermal conditions: 94 °C for 2 min; 35 cycles of 95 °C for 30 s, 52 °C for 30 s, and 72 °C for 1 min; and 72 °C for 10 min [22]. PCR products were checked and directly purified using a PCR Clean-up Kit (Macherey-Nagel, Germany). The purified PCR products were then sent to a commercial sequencing provider (1ST BASE Company, Kembangan, Malaysia). The sequences obtained from this study and other reference sequences obtained from GenBank were aligned using MUSCLE [55] and were then manually edited. Furthermore, jModelTest version 2.1.7 [56] was used to search for the selection of the best-fit nucleotide substitution models according to the Akaike Information Criterion. The phylogenetic tree was conducted using maximum likelihood (ML) and Bayesian inference (BI) algorithms and then implemented by RAxML version 7.0.3 [57] and MrBayes version 3.2.6 [58] following the method described in previous studies [3,22]. Bootstrap support (BS) and posterior probabilities (PP) values greater than or equal to 70% and 0.95, respectively, were significantly supported [59,60].

## 3. Results

### 3.1. General Soil Fungal Composition

**Figure 2:**
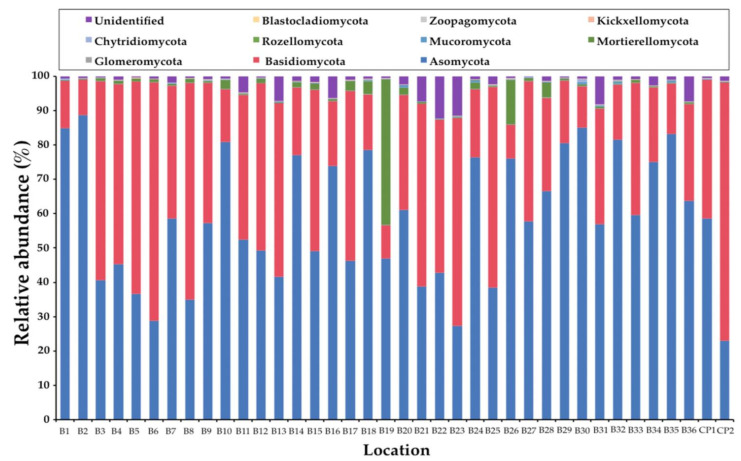
Soil fungal composition of each sample at the phylum level.

**Figure 3:**
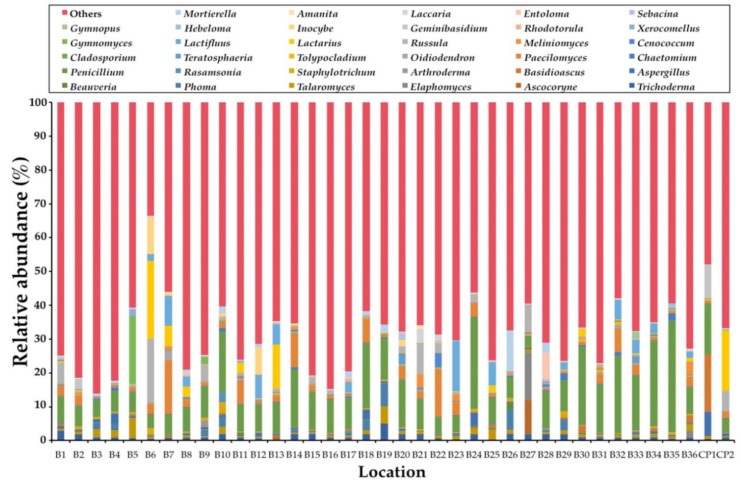
Soil fungal composition of each sample with the dominant of 35 fungal genera.

In this study, the obtained ITS1 sequences were not found to be fungal sequences within a range of 3% to 33%. This determination was dependent upon the samples (data not shown). Consequently, only the sequences belonging to the kingdom Fungi were analyzed. A total of 1,448,982 fungal amplicons from 38 soil samples were sequenced (Supplementary Table S2). The overall fungal community was assigned at the phylum-level as shown in Figure 1. The most abundant phylum was found in Ascomycota (57.63%) and followed by Basidiomycota (37.26%), unidentified (2.54%), Mortierellomycota (2.08%), Mucoromycota (0.24%), and Chytridiomycota (0.21%). Glomeromycota, Rozellomycota, and Kickxellomycota were found to be present in the same value at 0.01%. Subsequently, phyla Blastocladiomycota and Zoopagomycota were found at 0.007% and 0.003%, respectively. The abundant fungal phyla for each sample are presented in Figure 2. The relative degrees of abundance at the phylum-level varied in each soil sample. Ascomycota was represented at a range of 27.29% to 88.71% in soil samples of *B. alnoides* (*n* = 36) and 22.95% to 58.58% in soil samples of *C. poilanei* (*n* = 2). Twenty-three soil samples appear to be dominated by Ascomycota (>50%), of which the soil sample B2 revealed the highest degree of relative abundance. Soil samples of *B. alnoides* and *C. poilanei* revealed relative levels of abundance of Basidiomycota at 9.77% to 69.62% (*n* = 36) and 40.64% to 75.39% (*n* = 2), respectively. The highest relative abundance of Basidiomycota was found in sample B3. Notably, Chytridiomycota and Mortierellomycota were found in all samples. The highest relative abundance of Chytridiomycota (0.87%), Mortierellomycota (42.36%), and Mucoromycota (0.97%) was observed in soil samples B30, B19, and B20, respectively. Glomeromycota, Rozellomycota, Kickxellomycota, and Zoopagomycota were rarely found in each sample. Blastocladiomycota was only found in samples B5 and B15. However, Blastocladiomycota, Kickxellomycota, and Zoopagomycota were not observed in the soil samples of *C. poilanei*. Among the representative fungal amplicons, a final 1341 OTUs were obtained. The number of OTUs in Ascomycota, Basidiomycota, Blastocladiomycota, Chytridiomycota, Glomeromycota, Kickxellomycota, Mortierellomycota, Mucoromycota, Rozellomycota, Zoopagomycota, and unidentified were recorded at 811, 425, 2, 28, 8, 1, 19, 23, 14, 2, and 8, respectively (Supplementary Table S3). For deeper taxonomic assignments, a total of 35 classes, 99 orders, 240 families, and 549 genera were identified (Supplementary Table S4). It was found that the fungal genera varied in different soil samples. The relative abundance of the dominant 35 fungal genera among all soil samples is presented in Figure 3. Of the 35 most abundant fungal genera in the soil samples, the most abundant genera of mycorrhizal fungi were *Amanita*, *Cenococcum*, *Elaphomyces*, *Gymnomyces*, *Hebeloma*, *Inocybe*, *Lactifluus*, *Lactarius*, *Laccaria*, *Meliniomyces*, *Russula*, and *Xerocomellus*. The remaining 23 most abundant genera were identified as soil saprotrophs. Interestingly, the expected genus *Tuber* was also found in most of the soil samples. Additionally, the relative abundance of the genus *Tuber* in each soil sample is presented in Table 1. It was found that the relative abundance of this genus varied within a range of 0.01% to 4.00%. The highest relative abundance of the genus *Tuber* was found in sample B20.

### 3.2. OTUs of Truffles (Tuber Species) in Soil Samples

The results revealed that six OTUs representatively belonged to the genus *Tuber* (Table 2). OTU162, OTU187, OTU447, and OTU530 belonged to *T*. *thailandicum*, *T*. *lannaense*, *T. bomiense*, and *T*. *magnatum*, whereas OTU105 and OTU720 were unrecognized *Tuber* species. From 38 locations, truffle OTUs were not detected in five soil samples (B18, B19, B25, B30, and B35 locations) (Table 2). The remaining 33 locations were determined to be representative locations of truffle OTUs. Theses 33 locations included three previously known truffle locations (B2, B27, and CP2). *Tuber thailandicum* and *T. magnatum* were previously found in the locations B2 and CP2, respectively, while *T. lannaense* was previously found at the locations B27 and CP2 [22,23,24]. Therefore, 30 locations were identified as newly found truffle locations within our study area (Table 1). Twenty-three and three locations of *B*. *alnoides* were considered new locations for *T. thailandicum* and *T. bomiense*, respectively. Two locations of *B*. *alnoides* (B32 and B36) and one location of *C. poilanei* (CP1) indicated that they were new locations for *T. lannaense*. However, a new location for *T. magnatum* was not found. Additionally, eight new locations of *B*. *alnoides* were indicated for the unrecognized *Tuber* species by the presence of OTU105 and OTU720.

### 3.3. Truffle Hunting and Identification

**Figure 4:**
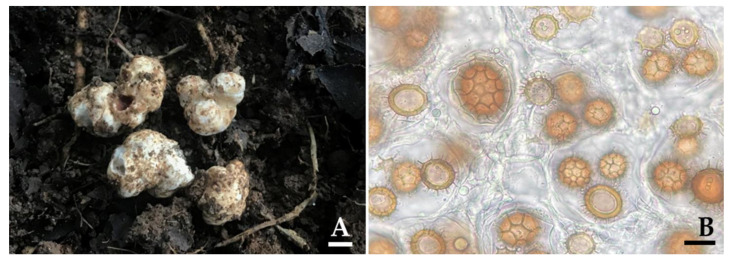
Ascocarps (**A**) and ascospores (**B**) of *Tuber thailadicum* collected from the representative locations of the truffle OTU (B14). Scale bar: A = 1 cm and B = 25 µm.

Truffle ascocarps were located following the results obtained from the eDNA data (Table 1) during the period from mid-May to September 2017. The results indicated that truffle ascocarps were found at 18 locations from a total of 30 locations, of which 16 locations were determined to be new truffle locations (Figure 4). However, truffle ascocarps were not found in the remaining 14 new locations. Truffle ascocarps were also not found in the absence of truffle OTUs locations. It was found that all 18 truffle ascocarps were obtained from the soil of *B. alnoides*. The morphological characteristics of 18 ascocarps that were collected from different locations are shown in Table 3. The results indicate that the morphological characteristics of ascocarps at each location were similar. Morphologically, all 18 ascocarps from each location were found to be quite similar to *T. thailandicum*. Ascocarps were 1.0−4.5 cm in diameter and white to light brown in color. Ascus contained one to four (rarely five) ascospores. Ascospores were globose to subglobose, sometimes broadly ellipsoid, 20–65 × 18–62 µm ornamented with a regular alveolate reticulum. Identification was confirmed by molecular phylogenetic analysis. The ITS sequence from ascocarps in B1, B2, B4, B5, B7, B10, B12, B13, B14, B15, B22, B26, B27, B29, B31, B32, B33, and B36 locations were deposited in GenBank under the numbers MW465642, MW465641, MW471002, MW471000, MW471001, MW470998, MW470999, MW470961, MW470960, MW470969, MW470965, MW470963, MW470962, MW470968, MW470964, MW470966, MW470967, and MW470970, respectively. For the phylogenetic analysis, sequences of 66 *Tuber* taxa were used, and *T. magnatum* was used as the outgroup. The alignment was deposited in TreeBASE under the study ID number 27568. A best scoring RAxML tree was established with a final ML optimization likelihood value of −8556.2711. The proportion of invariable sites and gamma distribution were 0.3890 and 0.5670, respectively. The average standard deviation of the split frequencies of the BI analysis is 0.00914.

**Figure 5:**
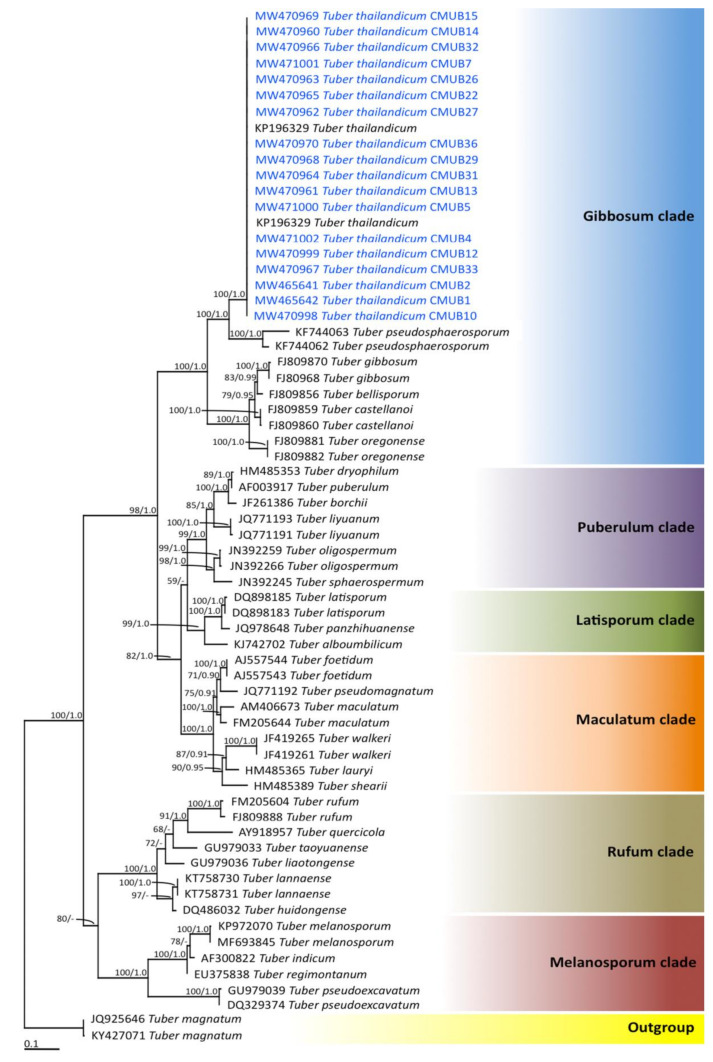
Phylogram derived from the maximum likelihood tree revealed by RAxML from an analysis of an ITS sequence of 66 taxa. *Tuber magnatum* was used as outgroup. The numbers above the branches represent Bootstrap support (BS) (**left**) and PP (**right**). Only BS values ≥ 70% and PP ≥ 0.90 are shown. “-” indicates a value of BS and PP of less than 70% and 0.90, respectively. The scale bar represents the expected number of nucleotide substitutions per site. Sequences obtained in this study are in blue.

The phylograms of the ML and BI analyses were found to be similar in topology (data not shown). Therefore, we have only presented the phylogram obtained from the ML analysis (Figure 5). Six main clades, namely Gibbosum, Puberulum, Latisporum, Maculatum, Rufum, and Melanosporum, were assigned according to the findings of previous phylogenetic studies [3,61,62]. Our phylogenetic results place all the specimens obtained from each location within the monophyletic clade of *T. thailandicum* in the Gibbosum clade with high support values (100% BS and 1.0 PP). Therefore, all truffle ascocarps obtained from each location were identified to *T. thailandicum* based on morphological and molecular data. However, ascocarps of other truffle species were not found at the new representative locations of the OTUs.

## 4. Discussion

An investigation of soil mycobiota diversity in forest soil using fungal metabarcoding analysis has provided valuable information on the variety of distinct functional groups of fungi, including mycorrhizal, saprotrophic, and pathogenic fungi [33,63,64,65]. We have hypothesized that the soil mycobiota diversity of the host plants of truffles could be used to investigate truffle diversity and to assist in locating truffles during the non-fruiting phase. In this study, soil samples obtained during the non-fruiting phase from known truffle host plants (*B. alnoides* and *C. poilanei*) of northern Thailand were collected, while soil mycobiota diversity was investigated. Our results indicate that Ascomycota had the highest overall relative abundance at the phylum level in the soil samples followed by Basidiomycota, Mortierellomycota, and Mucoromycota. Similarly, Ascomycota was found to be the dominant phylum in the soil of evergreen and deciduous forests in northeast Thailand [64]. Moreover, these results were supported by the findings of previous studies, which reported that Ascomycota was associated with high levels of distribution and abundance in global soil samples [66,67,68,69,70]. Our findings are similar to the findings of a number of previous studies [34,70,71,72,73,74,75] that found that *Inocybe, Lactarius, Meliniomyces*, and *Russula* are ectomycorrhizal-dominated fungal communities in the soil samples of tropical, temperate and boreal forests. Moreover, *Cortinarius, Piloderma*, and *Suillus* were also found to be dominant, especially in boreal forests [68,71,72]. In addition, *Meliniomyces* have been observed to be able to form both ectomycorrhiza and ericoidmycorrhiza [76,77]. Notably, certain previously published studies have reported that the geographical region, the specific season, the dominant tree species, nutrient availability, and the physical and chemical properties of the soil and soil depth all significantly affected the composition of the fungal community in the soil [37,68,71,78,79].

Metabarcoding studies on fungal diversity in natural environments have provided not only the functional groups of fungal compositions, but deeper insight into the compositions of the fungal species. In this study, six OTUs that were representative of the DNA of truffles included four known species (*T. bomiense*, *T*. *lannaense*, *T*. *magnatum*, and *T. thailandicum*). Additionally, two unrecognized truffle specimens were revealed by an investigation of the soil mycobiota diversity of *B. alnoides* and *C. poilanei*. Accordingly, several previous studies have used metagenomic analysis to monitor and detect some expected fungal genus and species in the environment [31,38,39,80,81]. Examples of these include the studies of Bai et al. [82] and Sommermann et al. [83], who used fungal metabarcoding data in agricultural soil to monitor and detect soil fungal pathogens. The distribution of truffle-like taxa (*Mesophellia*, *Hysterangium*, and *Chondrogaster*) in north-east Australian woodlands has been monitored by the mycorrhizal fungal community structure in root and soil samples [75]. Nowadays, several studies have applied the metabarcoding technique in the research and cultivation of truffles, including for the detection of *T. aestivum* and *T. melanosporum* in truffle orchards [84], the monitoring of the persistence of mycorrhization of *T. melanosporum* and *T. indicum* in the roots of their host plants [85,86], and a greater understanding of the relationship between truffles and other soil microorganisms (bacteria and fungi) for the improvement of the cultivation of several truffle species (*T*. *aestivum*, *T*. *indicum*, *T*. *pseudoexcavatum*, and *T*. *sinoaestivum*) along with their ascocarp development [85,87,88,89]. Consequently, the method of detection for the DNA of truffles in soil samples can be used in the investigation of truffle diversity and distribution in their natural habitat, as well as in the hunting of truffles during their fruiting period. As we might expect, the use of this method has resulted in the discovery of new species and new records of truffles in northern Thailand. Thus, the detection of ascocarps will help to prove and confirm the presence of each truffle species. Thirty locations were considered new locations for *T. bomiense*, *T*. *thailandicum*, while other unrecognized *Tuber* species and ascocarps of *T. thailandicum* were found that were undergoing the fruiting phase. Our results indicate that the use of the composition of fungal communities in soil samples can offer a new tool for the investigation and hunting of truffles. However, ascocarps of other truffle species were not found at the new OTUs representative locations. We found only the DNA of *T. bomiense* in the soil samples, but its ascocarps were not detected. Therefore, there is still a need to further hunt truffles in this study area. Furthermore, ascocarps of *T. lanaense* and *T. magnatum* were also not detected at previously known locations during our hunting period. Several previous studies have reported that multiple biotic and abiotic factors (e.g., temperature, humidity, soil nutrients, mating type, and soil microbial community) [2,4,9,90,91,92]. The suitable biotic and abiotic factors can influence ascocarp development in truffles. Moreover, seasonal climatic changes that occur during each year significantly affect ascocarp development in truffles [2,4,7,9]. According to the outcomes of our previous studies, ascocarps of *T. thailanicum* were recorded from mid-May to June [22] and ascocarps of both *T. lannaense* and *T. magnatum* were recorded from July to August [23,24], during which the humidity was recorded within a range of 70−75% and temperatures were reported within a range of 28−30 °C. From mid-May to June 2017, rain was observed in the study area and relative humidity was recorded within a range of 72−75%. Furthermore, temperatures were reported within a range of 28−30 °C. These conditions clearly supported the ascocarp development of truffles. However, from July to August, minimal amounts of rain were observed with low relative humidity readings within the range of 62−68% and high temperatures within the range of 29−33 °C (Northern Meteorological Center; http://www.cmmet.tmd.go.th, 30 June 2017). It is possible that during our hunting period, suitable conditions for the ascocarp development of other truffle species did not exist, with the exception of *T. thailandicum*. Notably, our study had two main limitations: (1) the absence of trained animals for truffle hunting and (2) a short-term period of observation. Consequently, future studies should focus on an evaluation of the efficiency of methods of truffle hunting using fungal community data, trained animals, and a combination of fungal community data and trained animals. Furthermore, long-term observations should be employed to provide an even greater understanding of the discovery and distribution of truffles, as well as to offer an expanded understanding of their fruiting periods within the study area.

## 5. Conclusions

Truffles form hypogeous ascocarps that are difficult to locate without the use of trained pigs and dogs. This study is the first of its kind to investigate the composition of the fungal community in soil samples of the host plants of truffles (*B. alnoides* and *C. poilanei*) in the native forests of northern Thailand. This was done with the objective of investigation of truffle diversity and locating truffles during the non-fruiting phase. From detected soil fungi, six OTUs that are representative of the DNA of truffles were found to include four known species (*T. bomiense*, *T*. *lannaense*, *T*. *magnatum*, and *T. thailandicum*) and two unrecognized truffle species. Thirty locations were considered new truffle locations, while truffle ascocarps were identified underground during the fruiting phase in 16 new locations. However, no truffle ascocarps were found in the remaining 14 new locations during the course of our investigation. All obtained truffles were identified as *T. thailandicum* based on the morphological and molecular data. There is still a need to further hunt truffles in our study area. Our study demonstrated that soil mycobiota diversity could be offered as a new tool in the investigation of truffle diversity and hunting for truffles without the use of trained animals. To the best of our knowledge, the results of this study can provide valuable information to researchers in terms of establishing a greater understanding of the distribution of truffles in their natural habitats based on soil mycobiota diversity. This would ultimately benefit the management and conservation of truffle habitats in northern Thailand as well as the future cultivation of truffles. Additionally, the outcomes of this study could facilitate a more efficient search for truffles and ultimately indicate their location. Further studies on the search for other truffle species from our describing soil mycobiota diversity are required in conjunction with long-term observations. A combination of using metabarcoding technics and trained animals in the hunt for truffles is needed in the future to evaluate the efficacy of these newly established truffle hunting methods. Moreover, soil mycobiota diversity of different plant species (e.g., chestnut, oak, and pine) should be investigated to fully understand the distribution of truffles in the natural forests of northern Thailand.

## Figures and Tables

**Table 1:** Data of DNA sequences of *Tuber* species and fruiting body obtained from each soil sample.

Sample No. *	Relative Abundant (%) ^†^	OTU Number						Observation of Fruiting Body
		105	162	187	447	530	720	
B1	0.02	–	+	–	–	–	–	+
B2 *	0.08	–	+	–	–	–	–	+
B3	0.04	–	+	–	–	–	+	–
B4	0.10	–	+	–	–	–	–	+
B5	0.05	–	+	–	–	–	–	+
B6	0.10	–	+	–	–	–	–	–
B7	0.20	+	+	–	–	–	–	+
B8	0.60	–	–	–	–	–	+	–
B9	0.07	+	–	–	–	–	–	–
B10	1.00	–	+	–	–	–	–	+
B11	0.03	–	+	–	–	–	–	–
B12	0.20	–	+	–	+	–	–	+
B13	0.06	–	+	–	–	–	–	+
B14	0.30	–	+	–	–	–	–	+
B15	0.03	–	+	–	–	–	–	+
B16	0.02	–	+	–	–	–	–	–
B17	0.02	+	–	–	–	–	–	–
B18	0.00	–	–	–	–	–	–	–
B19	0.00	–	–	–	–	–	–	–
B20	4.00	–	+	–	+	–	–	–
B21	0.02	–	–	–	–	–	+	–
B22	0.09	–	+	–	–	–	–	+
B23	0.20	–	+	–	–	–	–	–
B24	0.07	–	–	–	–	–	+	–
B25	0.00	–	–	–	–	–	–	–
B26	0.20	–	+	–	–	–	–	+
B27 *	0.90	–	+	+	–	–	–	+
B28	0.03	+	–	–	–	–	–	–
B29	0.04	–	+	–	–	–	–	+
B30	0.00	–	–	–	–	–	–	–
B31	0.03	–	+	–	–	–	–	+
B32	0.10	–	+	+	–	–	–	+
B33	0.03	–	+	–	–	–	–	+
B34	0.02	–	–	–	+	–	–	–
B35	0.00	–	–	–	–	–	–	–
B36	0.80	–	+	+	–	–	–	+
CP1	0.01	–	–	+	–	–	–	–
CP2 *	0.20	–	–	+	–	+	–	–
	Total found	4	24	5	3	1	4	18

* The previous known location. **^†^** Relative abundant of *Tuber* DNA was compared with the total fungal sequences in each sample. OTU162 = *T. thailandicum*, OTU187 = *T. lannaense*, OTU447 = *T. bomiense*, OTU530 = *T. magnatum*, OTU105 and OTU720 = unrecognized *Tuber* species. B = soil sample from *Betula alnoides* and CP = soil sample from *Carpinus poilanei*. “+” = presence/found and “–” = absence/not found.

**Table 2:** Detail of the DNA sequences of *Tuber* species obtained from soil using metabarcoding.

OTU Number	Length (bp)	GenBank Accession Number	Closeted Species/Accession Number	Similarity (%)
105	182	MW330007	*Tuber thailandicum* CMU-MTUF001/KP196331	97.52
162	238	MW326971	*Tuber thailandicum* CMU-MTUF001/KP196332	100
187	228	MW330257	*Tuber lanaense* CMU-MTUF007/KT758731	100
447	211	MW327594	*Tuber bomiense* SKM101/KC517480	100
530	164	MW326781	*Tuber magnatum* Tmag_Ca_09/MG992596	100
720	168	MW326083	*Tuber* sp. isolate JT9769/HM485407	100

**Table 3:** Morphological characteristics of truffles collected from each truffle OTU’s representative location in this study.

Location	Macromorphological Characteristics			Micromorphological Characteristics				
	Ascomata			Hair-like Structure in Peridium Surface	Number of Ascospore/Ascus	Ascospores		
	Size (cm in Diam)	Color	Gleba			Shape	Ornamentation	Size (μm)
B1	1.5–3.5	White	Brown	+	1–4	Globose to subglobose	Alveolate-reticulate	23–40 × 22–35
B2 *	2.2–4.3	White to pale yellow	Brown	+	1–4	Globose to broadly ellipsoid	Alveolate-reticulate	25–45 × 22–40
B4	2.0	White	Brown	+	1–4	Subglobose to broadly ellipsoid	Alveolate-reticulate	20–35 × 18–28
B5	1.0–3.0	Pale yellow to light brown	Brown to dark brown	+	1–4	Subglobose to ellipsoid	Alveolate-reticulate	25–55 × 22–48
B7	2.5–3.0	White to pale yellow	Brown	+	1–4	Globose to subglobose	Alveolate-reticulate	28–40 × 25–38
B10	1.5–3.5	White to pale yellow	Brown	+	1–4	Subglobose	Alveolate-reticulate	25–60 × 22–55
B12	2.5–4.5	Pale yellow to light brown	Dark brown	+	1–4	Subglobose to broadly ellipsoid	Alveolate-reticulate	25–43 × 22–35
B13	2.0	Pale yellow	Brown	+	1–4	Globose to broadly ellipsoid	Alveolate-reticulate	25–45 × 25–35
B14	1.0–3.2	White to pale yellow	Brown	+	1–4	Globose to subglobose	Alveolate-reticulate	26–42 × 25–38
B15	2.5	White	Brown	+	1–4	Subglobose	Alveolate-reticulate	20–50 × 18–47
B22	2.5–4.0	Pale yellow to light brown	Dark brown	+	1–4	Globose to subglobose	Alveolate-reticulate	20–65 × 18–62
B26	1.4–3.8	White	Brown	+	1–4	Globose to subglobose	Alveolate-reticulate	27–50 × 25–48
B27 *	1.0–3.2	White to pale yellow	Dark brown	+	1–4	Globose to broadly ellipsoid	Alveolate-reticulate	25–43 × 25–35
B29	3.5	White	Brown	+	1–4	Gubglobose to ellipsoid	Alveolate-reticulate	25–50 × 22–40
B31	2.0–4.1	White to pale yellow	Brown	+	1–5	Globose to broadly ellipsoid	Alveolate-reticulate	22–45 × 20–42
B32	2.0–3.0	Pale yellow to light brown	Brown to dark brown	+	1–4	Globose to subglobose	Alveolate-reticulate	25–45 × 20–35
B33	2.4–3.5	White to pale yellow	Brown	+	1–4	Subglobose to ellipsoid	Alveolate-reticulate	20–55 × 15–50
B36	1.5–3.0	White, pale yellow to light brown	Brown to dark brown	+	1–4	Subglobose to ellipsoid	Alveolate-reticulate	30–50 × 22–45

* The previous known location. B = soil sample from *Betula alnoides*. “+” = presence.

## Data Availability

The DNA sequence data obtained from this study have been deposited in GenBank under accession numbers; MW330007, MW326971, MW330257, MW327594, MW326781, MW326083MW465642, MW465641, MW471002, MW471000, MW471001, MW470998, MW470999, MW470961, MW470960, MW470969, MW470965, MW470963, MW470962, MW470968, MW470964, MW470966, MW470967, and MW470970.

## Supplementary Materials

The following are available online at https://www.mdpi.com/article/10.3390/jof7040293/s1, Table S1: Location of host plant in study site, Table S2: Soil fungal sequence reads from each soil sample organized by phylum-level, Table S3: The OTUs obtained in this study, Table S4: The fungal taxonomic assignments in this study.
