# Supplementary material for: Soil Metabarcoding Offers a New Tool for the Investigation and Hunting of Truffles in Northern Thailand
Source: J Fungi (Basel). 2021 Apr 13;7(4):293. doi: 10.3390/jof7040293 (PMC8069821; doi:10.3390/jof7040293)
Supplement: Supplementary file 1 [file jof-07-00293-s001.zip › Supplementary materials/jof-1176279-supp table 1.docx]

**Supplementary Table S1.** Location of host plants in study area.

| **Sample** | **Host plant** | **Location** | **Elevation (m)** |
| --- | --- | --- | --- |
| B1 | *Betula alnoides* | 18°40′30″N, 98°54′24″E | 1240 |
| B2 | *Betula alnoides* | 18°40′30″N, 98°54′25″E | 1240 |
| B3 | *Betula alnoides* | 18°47′54″N, 98°54′11″E | 1210 |
| B4 | *Betula alnoides* | 18°48′40″N, 98°54′14″E | 1335 |
| B5 | *Betula alnoides* | 18°48′43″N, 98°54′18″E | 1320 |
| B6 | *Betula alnoides* | 18°48′34″N, 98°54′17″E | 1325 |
| B7 | *Betula alnoides* | 18°48′43″N, 98°54′21″E | 1322 |
| B8 | *Betula alnoides* | 18°48′15″N, 98°54′15″E | 1273 |
| B9 | *Betula alnoides* | 18°48′13″N, 98°54′16″E | 1272 |
| B10 | *Betula alnoides* | 18°48′44″N, 98°53′46″E | 1503 |
| B11 | *Betula alnoides* | 18°48′44″N, 98°53′46″E | 1503 |
| B12 | *Betula alnoides* | 18°48′44″N, 98°53′47″E | 1498 |
| B13 | *Betula alnoides* | 18°48′49″N, 98°53′47″E | 1487 |
| B14 | *Betula alnoides* | 18°48′43″N, 98°53′46″E | 1497 |
| B15 | *Betula alnoides* | 18°48′43″N, 98°53′46″E | 1497 |
| B16 | *Betula alnoides* | 18°48′42″N, 98°53′44″E | 1508 |
| B17 | *Betula alnoides* | 18°48′41″N, 98°53′40″E | 1515 |
| B18 | *Betula alnoides* | 18°48′39″N, 98°53′41″E | 1504 |
| B19 | *Betula alnoides* | 18°48′40″N, 98°53′38″E | 1510 |
| B20 | *Betula alnoides* | 18°48′41″N, 98°53′36″E | 1512 |
| B21 | *Betula alnoides* | 18°48′40″N, 98°53′31″E | 1480 |
| B22 | *Betula alnoides* | 18°48′43″N, 98°53′32″E | 1501 |
| B23 | *Betula alnoides* | 18°48′41″N, 98°53′33″E | 1498 |
| B24 | *Betula alnoides* | 18°48′43″N, 98°53′35″E | 1517 |
| B25 | *Betula alnoides* | 18°48′44″N, 98°53′35″E | 1520 |
| B26 | *Betula alnoides* | 18°48′45″N, 98°53′36″E | 1531 |
| B27 | *Betula alnoides* | 18°50′20″N, 98°53′17″E | 1650 |
| B28 | *Betula alnoides* | 18°49′08″N, 98°53′34″E | 1508 |
| B29 | *Betula alnoides* | 18°49′10″N, 98°53′32″E | 1517 |
| B30 | *Betula alnoides* | 18°49′12″N, 98°53′32″E | 1516 |
| B31 | *Betula alnoides* | 18°49′25″N, 98°53′25″E | 1530 |
| B32 | *Betula alnoides* | 18°49′28″N, 98°53′23″E | 1550 |
| B33 | *Betula alnoides* | 18°49′29″N, 98°53′26″E | 1560 |
| B34 | *Betula alnoides* | 18°49′25″N, 98°53′19″E | 1577 |
| B35 | *Betula alnoides* | 18°49′30″N, 98°53′18″E | 1570 |
| B36 | *Betula alnoides* | 18°49′24″N, 98°53′20″E | 1564 |
| CP1 | *Carpinus poilanei* | 18°49′15″N, 98°53′30″E | 1534 |
| CP2 | *Carpinus poilanei* | 18°49′52″N, 98°54′12″E | 1580 |
