# Supplementary material for: Soil Metabarcoding Offers a New Tool for the Investigation and Hunting of Truffles in Northern Thailand
Source: J Fungi (Basel). 2021 Apr 13;7(4):293. doi: 10.3390/jof7040293 (PMC8069821; doi:10.3390/jof7040293)
Supplement: Supplementary file 1 [file jof-07-00293-s001.zip › Supplementary materials/jof-1176279-supp table 2.docx]

**Supplementary Table S2.** Soil fungal sequence reads from each soil sample organized by phylum-level.

| **Sample No.** | **Phylum-level** | | | | | | | | | | | **Total** |
| --- | --- | --- | --- | --- | --- | --- | --- | --- | --- | --- | --- | --- |
|  | **AS** | **BA** | **GL** | **MO** | **MU** | **RO** | **CHY** | **KIC** | **ZO** | **BLA** | **UN** |  |
| B1 | 19,086 | 3,134 | 5 | 23 | 31 | 0 | 51 | 0 | 0 | 0 | 162 | 22,492 |
| B2 | 35,230 | 4,056 | 0 | 165 | 22 | 0 | 37 | 5 | 0 | 0 | 198 | 39,713 |
| B3 | 16,676 | 23,830 | 4 | 328 | 22 | 2 | 33 | 2 | 2 | 0 | 157 | 41,056 |
| B4 | 18,603 | 21,555 | 6 | 362 | 31 | 2 | 35 | 6 | 0 | 0 | 443 | 41,043 |
| B5 | 12,999 | 21,958 | 0 | 282 | 33 | 0 | 39 | 8 | 0 | 2 | 146 | 35,467 |
| B6 | 11,578 | 28,009 | 1 | 293 | 22 | 1 | 51 | 8 | 0 | 0 | 146 | 40,230 |
| B7 | 24,507 | 16,159 | 1 | 230 | 86 | 0 | 23 | 0 | 0 | 0 | 784 | 41,790 |
| B8 | 14,431 | 26,102 | 3 | 415 | 53 | 1 | 23 | 0 | 0 | 1 | 239 | 41,268 |
| B9 | 22,989 | 16,397 | 7 | 164 | 34 | 3 | 216 | 0 | 0 | 1 | 332 | 40,143 |
| B10 | 23,469 | 4,523 | 4 | 706 | 33 | 4 | 81 | 15 | 1 | 0 | 203 | 29,039 |
| B11 | 21,890 | 17,646 | 2 | 64 | 63 | 3 | 161 | 4 | 0 | 0 | 1,944 | 41,777 |
| B12 | 19,486 | 19,300 | 0 | 504 | 107 | 4 | 20 | 4 | 0 | 0 | 140 | 39,565 |
| B13 | 17,481 | 21,249 | 2 | 52 | 185 | 0 | 9 | 3 | 0 | 0 | 2,981 | 41,962 |
| B14 | 31,617 | 8,096 | 1 | 552 | 138 | 4 | 66 | 2 | 0 | 0 | 541 | 41,017 |
| B15 | 19,155 | 18,410 | 6 | 670 | 57 | 10 | 54 | 16 | 0 | 3 | 667 | 39,048 |
| B16 | 30,182 | 7,780 | 4 | 176 | 57 | 0 | 101 | 4 | 0 | 0 | 2,574 | 40,878 |
| B17 | 18,857 | 20,237 | 2 | 1,152 | 54 | 1 | 52 | 37 | 0 | 0 | 422 | 40,814 |
| B18 | 30,087 | 6,226 | 7 | 1,452 | 106 | 2 | 198 | 2 | 0 | 0 | 242 | 38,322 |
| B19 | 15,922 | 3,314 | 1 | 14,362 | 33 | 9 | 65 | 14 | 0 | 0 | 188 | 33,908 |
| B20 | 20,919 | 11,503 | 10 | 696 | 321 | 4 | 28 | 3 | 0 | 0 | 791 | 34,275 |
| B21 | 16,208 | 22,256 | 0 | 161 | 92 | 3 | 26 | 0 | 0 | 0 | 3,030 | 41,776 |
| B22 | 16,038 | 16,697 | 0 | 63 | 51 | 0 | 13 | 0 | 0 | 0 | 4,631 | 37,493 |
| B23 | 11,252 | 24,953 | 0 | 75 | 187 | 3 | 19 | 2 | 0 | 2 | 4,741 | 41,234 |
| B24 | 29,183 | 7,610 | 2 | 667 | 348 | 4 | 24 | 3 | 0 | 0 | 331 | 38,172 |
| B25 | 15,659 | 23,959 | 1 | 37 | 74 | 1 | 160 | 0 | 0 | 1 | 918 | 40,810 |
| B26 | 23,964 | 3,129 | 9 | 4,068 | 15 | 7 | 124 | 1 | 2 | 0 | 182 | 31,501 |
| B27 | 23,220 | 16,431 | 5 | 339 | 30 | 0 | 84 | 0 | 0 | 0 | 70 | 40,179 |
| B28 | 13,996 | 5,703 | 15 | 932 | 21 | 0 | 60 | 3 | 0 | 0 | 283 | 21,013 |
| B29 | 26,620 | 6,078 | 1 | 141 | 35 | 0 | 86 | 8 | 0 | 0 | 97 | 33,066 |
| B30 | 33,055 | 4,774 | 2 | 182 | 272 | 0 | 338 | 8 | 0 | 0 | 271 | 38,902 |
| B31 | 22,951 | 13,579 | 14 | 237 | 98 | 0 | 168 | 5 | 0 | 0 | 3,268 | 40,320 |
| B32 | 33,199 | 6,519 | 8 | 113 | 231 | 2 | 209 | 6 | 0 | 0 | 400 | 40,687 |
| B33 | 23,672 | 15,336 | 9 | 286 | 37 | 2 | 78 | 1 | 0 | 0 | 336 | 39,757 |
| B34 | 30,374 | 8,797 | 3 | 82 | 53 | 1 | 74 | 3 | 0 | 0 | 1,073 | 40,460 |
| B35 | 33,773 | 6,036 | 5 | 40 | 318 | 0 | 69 | 2 | 0 | 0 | 382 | 40,625 |
| B36 | 22,415 | 9,910 | 5 | 112 | 43 | 4 | 79 | 20 | 2 | 0 | 2,555 | 35,145 |
| CP1 | 24,714 | 17,145 | 5 | 0 | 6 | 0 | 102 | 1 | 0 | 0 | 216 | 42,189 |
| CP2 | 9,603 | 31,546 | 4 | 25 | 55 | 0 | 21 | 1 | 0 | 0 | 591 | 41,846 |
| Total | 835,060 | 539,942 | 154 | 30,208 | 3,454 | 77 | 3,077 | 197 | 7 | 10 | 3,6796 | 1,448,982 |
| % | 57.63 | 37.26 | 0.01 | 2.08 | 0.24 | 0.01 | 0.21 | 0.01 | 0.003 | 0.007 | 2.54 | 100 |

B = soil sample from *Betula alnoides* and CP = soil sample from *Carpinus poilanei*.

AS = Ascomycota, BA = Basidiomycota, GL = Glomeromycota, MO = Mortierellomycota, MU = Mucoromycota,

RO = Rozellomycota, CHY = Chytridiomycota, KIC = Kickxellomycota, ZO = Zoopagomycota,

BLA = Blastocladiomycota and UN = Unidentified.
