# Supplementary material for: Soil Metabarcoding Offers a New Tool for the Investigation and Hunting of Truffles in Northern Thailand
Source: J Fungi (Basel). 2021 Apr 13;7(4):293. doi: 10.3390/jof7040293 (PMC8069821; doi:10.3390/jof7040293)
Supplement: Supplementary file 1 [file jof-07-00293-s001.zip › Supplementary materials/jof-1176279-supp table 4.docx]

**Supplementary Table S4.** The fungal taxonomic assignments in this study.

**Ascomycota**

| **OUT number** | **Taxonomy** |
| --- | --- |
| OTU502 | k__Fungi;p__Ascomycota;c__Archaeorhizomycetes;o__Archaeorhizomycetales;f__Archaeorhizomycetaceae;g__Archaeorhizomyces;s__unidentified |
| OTU625 | k__Fungi;p__Ascomycota;c__Arthoniomycetes;o__Arthoniales;f__Roccellaceae;g__Dendrographa;s__Dendrographa_leucophaea_f._minor |
| OTU21 | k__Fungi;p__Ascomycota;c__Dothideomycetes;o__Acrospermales;f__Acrospermales_fam_Incertae_sedis;g__Leptodiscella;s__Leptodiscella_africana |
| OTU612 | k__Fungi;p__Ascomycota;c__Dothideomycetes;o__Botryosphaeriales;f__Aplosporellaceae;g__Aplosporella;s__Aplosporella_javeedii |
| OTU60 | k__Fungi;p__Ascomycota;c__Dothideomycetes;o__Botryosphaeriales;f__Botryosphaeriaceae;g__Diplodia;s__Diplodia_intermedia |
| OTU1151 | k__Fungi;p__Ascomycota;c__Dothideomycetes;o__Botryosphaeriales;f__Botryosphaeriaceae;g__Dothiorella;s__Dothiorella_thailandica |
| OTU140 | k__Fungi;p__Ascomycota;c__Dothideomycetes;o__Botryosphaeriales;f__Botryosphaeriaceae;g__Lasiodiplodia;s__Lasiodiplodia_jatrophicola |
| OTU375 | k__Fungi;p__Ascomycota;c__Dothideomycetes;o__Botryosphaeriales;f__Botryosphaeriaceae;g__unidentified;s__unidentified |
| OTU671 | k__Fungi;p__Ascomycota;c__Dothideomycetes;o__Botryosphaeriales;f__Saccharataceae;g__Saccharata;s__Saccharata_hakeicola |
| OTU999 | k__Fungi;p__Ascomycota;c__Dothideomycetes;o__Capnodiales;f__Cladosporiaceae;g__Cladosporium;s__Cladosporium_aphidis |
| OTU836 | k__Fungi;p__Ascomycota;c__Dothideomycetes;o__Capnodiales;f__Cladosporiaceae;g__Cladosporium;s__Cladosporium_austrohemisphaericum |
| OTU218 | k__Fungi;p__Ascomycota;c__Dothideomycetes;o__Capnodiales;f__Cladosporiaceae;g__Cladosporium;s__Cladosporium_cladosporioides |
| OTU90 | k__Fungi;p__Ascomycota;c__Dothideomycetes;o__Capnodiales;f__Cladosporiaceae;g__Cladosporium;s__Cladosporium_delicatulum |
| OTU681 | k__Fungi;p__Ascomycota;c__Dothideomycetes;o__Capnodiales;f__Cladosporiaceae;g__Cladosporium;s__Cladosporium_fusiforme |
| OTU391 | k__Fungi;p__Ascomycota;c__Dothideomycetes;o__Capnodiales;f__Cladosporiaceae;g__Cladosporium;s__Cladosporium_sphaerospermum |
| OTU287 | k__Fungi;p__Ascomycota;c__Dothideomycetes;o__Capnodiales;f__Cladosporiaceae;g__Cladosporium;s__Cladosporium_velox |
| OTU369 | k__Fungi;p__Ascomycota;c__Dothideomycetes;o__Capnodiales;f__Cladosporiaceae;g__Rachicladosporium;s__Rachicladosporium_cboliae |
| OTU363 | k__Fungi;p__Ascomycota;c__Dothideomycetes;o__Capnodiales;f__Cladosporiaceae;g__Rachicladosporium;s__Rachicladosporium_eucalypti |
| OTU111 | k__Fungi;p__Ascomycota;c__Dothideomycetes;o__Capnodiales;f__Cladosporiaceae;g__Rachicladosporium;s__unidentified |
| OTU1085 | k__Fungi;p__Ascomycota;c__Dothideomycetes;o__Capnodiales;f__Mycosphaerellaceae;g__Houjia;s__Houjia_yanglingensis |
| OTU1258 | k__Fungi;p__Ascomycota;c__Dothideomycetes;o__Capnodiales;f__Mycosphaerellaceae;g__Pseudocercospora;s__Pseudocercospora_assamensis |
| OTU684 | k__Fungi;p__Ascomycota;c__Dothideomycetes;o__Capnodiales;f__Mycosphaerellaceae;g__unidentified;s__unidentified |
| OTU199 | k__Fungi;p__Ascomycota;c__Dothideomycetes;o__Capnodiales;f__Teratosphaeriaceae;g__unidentified;s__unidentified |
| OTU316 | k__Fungi;p__Ascomycota;c__Dothideomycetes;o__Capnodiales;f__unidentified;g__unidentified;s__unidentified |
| OTU646 | k__Fungi;p__Ascomycota;c__Dothideomycetes;o__Dothideales;f__Aureobasidiaceae;g__Aureobasidium;s__Aureobasidium_namibiae |
| OTU826 | k__Fungi;p__Ascomycota;c__Dothideomycetes;o__Dothideales;f__Aureobasidiaceae;g__Aureobasidium;s__Aureobasidium_thailandense |
| OTU1094 | k__Fungi;p__Ascomycota;c__Dothideomycetes;o__Dothideales;f__Aureobasidiaceae;g__Kabatiella;s__unidentified |
| OTU884 | k__Fungi;p__Ascomycota;c__Dothideomycetes;o__Dothideales;f__Dothideaceae;g__Rhizosphaera;s__Rhizosphaera_kalkhoffii |
| OTU224 | k__Fungi;p__Ascomycota;c__Dothideomycetes;o__Dothideales;f__Dothioraceae;g__Hormonema;s__Hormonema_macrosporum |
| OTU193 | k__Fungi;p__Ascomycota;c__Dothideomycetes;o__Dothideales;f__Dothioraceae;g__Hormonema;s__Hormonema_viticola |
| OTU538 | k__Fungi;p__Ascomycota;c__Dothideomycetes;o__Dothideales;f__unidentified;g__unidentified;s__unidentified |
| OTU588 | k__Fungi;p__Ascomycota;c__Dothideomycetes;o__Dothideales;f__unidentified;g__unidentified;s__unidentified |
| OTU928 | k__Fungi;p__Ascomycota;c__Dothideomycetes;o__Hysteriales;f__Hysteriaceae;g__Rhytidhysteron;s__Rhytidhysteron_rufulum |
| OTU24 | k__Fungi;p__Ascomycota;c__Dothideomycetes;o__Jahnulales;f__Jahnulales_fam_Incertae_sedis;g__Speiropsis;s__Speiropsis_pedatospora |
| OTU77 | k__Fungi;p__Ascomycota;c__Dothideomycetes;o__Minutisphaerales;f__Minutisphaeraceae;g__Minutisphaera;s__Minutisphaera_aspera |
| OTU1171 | k__Fungi;p__Ascomycota;c__Dothideomycetes;o__Myriangiales;f__Elsinoaceae;g__Elsinoe;s__Elsinoe_hederae |
| OTU766 | k__Fungi;p__Ascomycota;c__Dothideomycetes;o__Myriangiales;f__unidentified;g__unidentified;s__unidentified |
| OTU995 | k__Fungi;p__Ascomycota;c__Dothideomycetes;o__Mytilinidales;f__Gloniaceae;g__Cenococcum;s__Cenococcum_geophilum |
| OTU730 | k__Fungi;p__Ascomycota;c__Dothideomycetes;o__Mytilinidales;f__Gloniaceae;g__unidentified;s__unidentified |
| OTU1004 | k__Fungi;p__Ascomycota;c__Dothideomycetes;o__Mytilinidiales;f__Mytilinidiaceae;g__Pseudocamaropycnis;s__Pseudocamaropycnis_pini |
| OTU1327 | k__Fungi;p__Ascomycota;c__Dothideomycetes;o__Pleosporales;f__Cucurbitariaceae;g__Pyrenochaeta;s__Pyrenochaeta_inflorescentiae |
| OTU344 | k__Fungi;p__Ascomycota;c__Dothideomycetes;o__Pleosporales;f__Cucurbitariaceae;g__Pyrenochaeta;s__Pyrenochaeta_lycopersici |
| OTU23 | k__Fungi;p__Ascomycota;c__Dothideomycetes;o__Pleosporales;f__Cucurbitariaceae;g__Pyrenochaeta;s__unidentified |
| OTU882 | k__Fungi;p__Ascomycota;c__Dothideomycetes;o__Pleosporales;f__Cucurbitariaceae;g__Pyrenochaetopsis;s__Pyrenochaetopsis_tabarestanensis |
| OTU500 | k__Fungi;p__Ascomycota;c__Dothideomycetes;o__Pleosporales;f__Cucurbitariaceae;g__Pyrenochaetopsis;s__unidentified |
| OTU970 | k__Fungi;p__Ascomycota;c__Dothideomycetes;o__Pleosporales;f__Dictyosporiaceae;g__Dictyocheirospora;s__Dictyocheirospora_garethjonesii |
| OTU574 | k__Fungi;p__Ascomycota;c__Dothideomycetes;o__Pleosporales;f__Dictyosporiaceae;g__Dictyosporium;s__Dictyosporium_strelitziae |
| OTU188 | k__Fungi;p__Ascomycota;c__Dothideomycetes;o__Pleosporales;f__Dictyosporiaceae;g__Pseudocoleophoma;s__Pseudocoleophoma_bauhiniae |
| OTU264 | k__Fungi;p__Ascomycota;c__Dothideomycetes;o__Pleosporales;f__Dictyosporiaceae;g__Pseudodictyosporium;s__Pseudodictyosporium_elegans |
| OTU941 | k__Fungi;p__Ascomycota;c__Dothideomycetes;o__Pleosporales;f__Didymellaceae;g__Allophoma;s__Allophoma_tropica |
| OTU389 | k__Fungi;p__Ascomycota;c__Dothideomycetes;o__Pleosporales;f__Didymellaceae;g__Didymella;s__Didymella_curtisii |
| OTU329 | k__Fungi;p__Ascomycota;c__Dothideomycetes;o__Pleosporales;f__Didymellaceae;g__Didymella;s__Didymella_fabae |
| OTU973 | k__Fungi;p__Ascomycota;c__Dothideomycetes;o__Pleosporales;f__Didymellaceae;g__Didymella;s__Didymella_glomerata |
| OTU169 | k__Fungi;p__Ascomycota;c__Dothideomycetes;o__Pleosporales;f__Didymellaceae;g__Didymella;s__Didymella_musae |
| OTU156 | k__Fungi;p__Ascomycota;c__Dothideomycetes;o__Pleosporales;f__Didymellaceae;g__Ectophoma;s__Ectophoma_pomi |
| OTU282 | k__Fungi;p__Ascomycota;c__Dothideomycetes;o__Pleosporales;f__Didymellaceae;g__Epicoccum;s__Epicoccum_brasiliense |
| OTU1180 | k__Fungi;p__Ascomycota;c__Dothideomycetes;o__Pleosporales;f__Didymellaceae;g__Epicoccum;s__Epicoccum_thailandicum |
| OTU838 | k__Fungi;p__Ascomycota;c__Dothideomycetes;o__Pleosporales;f__Didymellaceae;g__Neodidymelliopsis;s__Neodidymelliopsis_ranunculi |
| OTU1042 | k__Fungi;p__Ascomycota;c__Dothideomycetes;o__Pleosporales;f__Didymellaceae;g__Paraboeremia;s__Paraboeremia_selaginellae |
| OTU1265 | k__Fungi;p__Ascomycota;c__Dothideomycetes;o__Pleosporales;f__Didymellaceae;g__Phoma;s__unidentified |
| OTU1001 | k__Fungi;p__Ascomycota;c__Dothideomycetes;o__Pleosporales;f__Didymellaceae;g__Stagonosporopsis;s__Stagonosporopsis_astragali |
| OTU659 | k__Fungi;p__Ascomycota;c__Dothideomycetes;o__Pleosporales;f__Didymellaceae;g__Stagonosporopsis;s__Stagonosporopsis_dorenboschii |
| OTU272 | k__Fungi;p__Ascomycota;c__Dothideomycetes;o__Pleosporales;f__Didymellaceae;g__Stagonosporopsis;s__Stagonosporopsis_sp |
| OTU916 | k__Fungi;p__Ascomycota;c__Dothideomycetes;o__Pleosporales;f__Didymellaceae;g__unidentified;s__unidentified |
| OTU614 | k__Fungi;p__Ascomycota;c__Dothideomycetes;o__Pleosporales;f__Didymosphaeriaceae;g__Alloconiothyrium;s__Alloconiothyrium_aptrootii |
| OTU1241 | k__Fungi;p__Ascomycota;c__Dothideomycetes;o__Pleosporales;f__Didymosphaeriaceae;g__Laburnicola;s__Laburnicola_dactylidis |
| OTU522 | k__Fungi;p__Ascomycota;c__Dothideomycetes;o__Pleosporales;f__Didymosphaeriaceae;g__Paraconiothyrium;s__Paraconiothyrium_archidendri |
| OTU239 | k__Fungi;p__Ascomycota;c__Dothideomycetes;o__Pleosporales;f__Didymosphaeriaceae;g__Paraconiothyrium;s__Paraconiothyrium_thysanolaenae |
| OTU779 | k__Fungi;p__Ascomycota;c__Dothideomycetes;o__Pleosporales;f__Didymosphaeriaceae;g__Paraconiothyrium;s__Paraconiothyrium_variabile |
| OTU1234 | k__Fungi;p__Ascomycota;c__Dothideomycetes;o__Pleosporales;f__Didymosphaeriaceae;g__Paraphaeosphaeria;s__Paraphaeosphaeria_neglecta |
| OTU1208 | k__Fungi;p__Ascomycota;c__Dothideomycetes;o__Pleosporales;f__Didymosphaeriaceae;g__Paraphaeosphaeria;s__Paraphaeosphaeria_parmeliae |
| OTU430 | k__Fungi;p__Ascomycota;c__Dothideomycetes;o__Pleosporales;f__Didymosphaeriaceae;g__Paraphaeosphaeria;s__unidentified |
| OTU59 | k__Fungi;p__Ascomycota;c__Dothideomycetes;o__Pleosporales;f__Didymosphaeriaceae;g__Spegazzinia;s__Spegazzinia_parkeri |
| OTU210 | k__Fungi;p__Ascomycota;c__Dothideomycetes;o__Pleosporales;f__Didymosphaeriaceae;g__unidentified;s__unidentified |
| OTU1336 | k__Fungi;p__Ascomycota;c__Dothideomycetes;o__Pleosporales;f__Didymosphaeriaceae;g__Xenocamarosporium;s__Xenocamarosporium_acaciae |
| OTU94 | k__Fungi;p__Ascomycota;c__Dothideomycetes;o__Pleosporales;f__Leptosphaeriaceae;g__Ampelomyces;s__Ampelomyces_quisqualis |
| OTU45 | k__Fungi;p__Ascomycota;c__Dothideomycetes;o__Pleosporales;f__Leptosphaeriaceae;g__Plenodomus;s__Plenodomus_biglobosus |
| OTU1340 | k__Fungi;p__Ascomycota;c__Dothideomycetes;o__Pleosporales;f__Lophiostomataceae;g__Lophiostoma;s__unidentified |
| OTU382 | k__Fungi;p__Ascomycota;c__Dothideomycetes;o__Pleosporales;f__Lophiostomataceae;g__Pseudopaucispora;s__Pseudopaucispora_brunneospora |
| OTU1157 | k__Fungi;p__Ascomycota;c__Dothideomycetes;o__Pleosporales;f__Lophiotremataceae;g__Lophiotrema;s__Lophiotrema_rubi |
| OTU831 | k__Fungi;p__Ascomycota;c__Dothideomycetes;o__Pleosporales;f__Massarinaceae;g__Helminthosporium;s__Helminthosporium_velutinum |
| OTU345 | k__Fungi;p__Ascomycota;c__Dothideomycetes;o__Pleosporales;f__Massarinaceae;g__Massarina;s__unidentified |
| OTU1118 | k__Fungi;p__Ascomycota;c__Dothideomycetes;o__Pleosporales;f__Melanommataceae;g__Farlowiella;s__Farlowiella_carmichaeliana |
| OTU981 | k__Fungi;p__Ascomycota;c__Dothideomycetes;o__Pleosporales;f__Parabambusicolaceae;g__Multiseptospora;s__Multiseptospora_thailandica |
| OTU350 | k__Fungi;p__Ascomycota;c__Dothideomycetes;o__Pleosporales;f__Periconiaceae;g__Periconia;s__Periconia_cookei |
| OTU491 | k__Fungi;p__Ascomycota;c__Dothideomycetes;o__Pleosporales;f__Periconiaceae;g__Periconia;s__Periconia_echinochloae |
| OTU524 | k__Fungi;p__Ascomycota;c__Dothideomycetes;o__Pleosporales;f__Periconiaceae;g__Periconia;s__Periconia_macrospinosa |
| OTU1119 | k__Fungi;p__Ascomycota;c__Dothideomycetes;o__Pleosporales;f__Periconiaceae;g__Periconia;s__unidentified |
| OTU1215 | k__Fungi;p__Ascomycota;c__Dothideomycetes;o__Pleosporales;f__Phaeosphaeriaceae;g__Leptospora;s__Leptospora_thailandica |
| OTU939 | k__Fungi;p__Ascomycota;c__Dothideomycetes;o__Pleosporales;f__Phaeosphaeriaceae;g__Ophiosphaerella;s__Ophiosphaerella_herpotricha |
| OTU900 | k__Fungi;p__Ascomycota;c__Dothideomycetes;o__Pleosporales;f__Phaeosphaeriaceae;g__Ophiosphaerella;s__unidentified |
| OTU564 | k__Fungi;p__Ascomycota;c__Dothideomycetes;o__Pleosporales;f__Phaeosphaeriaceae;g__Phaeosphaeria;s__Phaeosphaeria_chiangraina |
| OTU108 | k__Fungi;p__Ascomycota;c__Dothideomycetes;o__Pleosporales;f__Phaeosphaeriaceae;g__Phaeosphaeria;s__unidentified |
| OTU1164 | k__Fungi;p__Ascomycota;c__Dothideomycetes;o__Pleosporales;f__Phaeosphaeriaceae;g__Setophoma;s__Setophoma_chromolaenae |
| OTU44 | k__Fungi;p__Ascomycota;c__Dothideomycetes;o__Pleosporales;f__Phaeosphaeriaceae;g__Setophoma;s__Setophoma_endophytica |
| OTU1271 | k__Fungi;p__Ascomycota;c__Dothideomycetes;o__Pleosporales;f__Phaeosphaeriaceae;g__Setophoma;s__Setophoma_longinqua |
| OTU752 | k__Fungi;p__Ascomycota;c__Dothideomycetes;o__Pleosporales;f__Phaeosphaeriaceae;g__Setophoma;s__Setophoma_yunnanensis |
| OTU398 | k__Fungi;p__Ascomycota;c__Dothideomycetes;o__Pleosporales;f__Phaeosphaeriaceae;g__Setophoma;s__unidentified |
| OTU733 | k__Fungi;p__Ascomycota;c__Dothideomycetes;o__Pleosporales;f__Phaeosphaeriaceae;g__unidentified;s__unidentified |
| OTU1121 | k__Fungi;p__Ascomycota;c__Dothideomycetes;o__Pleosporales;f__Pleosporaceae;g__Alternaria;s__Alternaria_chlamydospora |
| OTU987 | k__Fungi;p__Ascomycota;c__Dothideomycetes;o__Pleosporales;f__Pleosporaceae;g__Alternaria;s__Alternaria_chlamydosporigena |
| OTU181 | k__Fungi;p__Ascomycota;c__Dothideomycetes;o__Pleosporales;f__Pleosporaceae;g__Alternaria;s__Alternaria_hungarica |
| OTU1178 | k__Fungi;p__Ascomycota;c__Dothideomycetes;o__Pleosporales;f__Pleosporaceae;g__Alternaria;s__Alternaria_tenuissima |
| OTU335 | k__Fungi;p__Ascomycota;c__Dothideomycetes;o__Pleosporales;f__Pleosporaceae;g__Bipolaris;s__Bipolaris_maydis |
| OTU485 | k__Fungi;p__Ascomycota;c__Dothideomycetes;o__Pleosporales;f__Pleosporaceae;g__Bipolaris;s__Bipolaris_shoemakeri |
| OTU509 | k__Fungi;p__Ascomycota;c__Dothideomycetes;o__Pleosporales;f__Pleosporaceae;g__Comoclathris;s__Comoclathris_italica |
| OTU1079 | k__Fungi;p__Ascomycota;c__Dothideomycetes;o__Pleosporales;f__Pleosporaceae;g__Curvularia;s__Curvularia_caricae-papayae |
| OTU1244 | k__Fungi;p__Ascomycota;c__Dothideomycetes;o__Pleosporales;f__Pleosporaceae;g__Curvularia;s__Curvularia_lunata |
| OTU1288 | k__Fungi;p__Ascomycota;c__Dothideomycetes;o__Pleosporales;f__Pleosporaceae;g__Curvularia;s__Curvularia_sorghina |
| OTU1240 | k__Fungi;p__Ascomycota;c__Dothideomycetes;o__Pleosporales;f__Pleosporaceae;g__Curvularia;s__Curvularia_spicifera |
| OTU124 | k__Fungi;p__Ascomycota;c__Dothideomycetes;o__Pleosporales;f__Pleosporaceae;g__Curvularia;s__Curvularia_tuberculata |
| OTU1012 | k__Fungi;p__Ascomycota;c__Dothideomycetes;o__Pleosporales;f__Pleosporaceae;g__Stemphylium;s__Stemphylium_beticola |
| OTU413 | k__Fungi;p__Ascomycota;c__Dothideomycetes;o__Pleosporales;f__Pleosporaceae;g__Stemphylium;s__Stemphylium_lycopersici |
| OTU324 | k__Fungi;p__Ascomycota;c__Dothideomycetes;o__Pleosporales;f__Pleosporaceae;g__unidentified;s__unidentified |
| OTU669 | k__Fungi;p__Ascomycota;c__Dothideomycetes;o__Pleosporales;f__Pleosporales_fam_Incertae_sedis;g__Nigrograna;s__unidentified |
| OTU851 | k__Fungi;p__Ascomycota;c__Dothideomycetes;o__Pleosporales;f__Pleosporales_fam_Incertae_sedis;g__Polyschema;s__Polyschema_sclerotigenum |
| OTU1199 | k__Fungi;p__Ascomycota;c__Dothideomycetes;o__Pleosporales;f__Pleosporales_fam_Incertae_sedis;g__Pseudochaetosphaeronema;s__Pseudochaetosphaeronema_larense |
| OTU901 | k__Fungi;p__Ascomycota;c__Dothideomycetes;o__Pleosporales;f__Sporormiaceae;g__Preussia;s__Preussia_tenerifae |
| OTU488 | k__Fungi;p__Ascomycota;c__Dothideomycetes;o__Pleosporales;f__Sporormiaceae;g__Preussia;s__unidentified |
| OTU781 | k__Fungi;p__Ascomycota;c__Dothideomycetes;o__Pleosporales;f__Sporormiaceae;g__unidentified;s__unidentified |
| OTU1298 | k__Fungi;p__Ascomycota;c__Dothideomycetes;o__Pleosporales;f__Teichosporaceae;g__Teichospora;s__Teichospora_nephelii |
| OTU624 | k__Fungi;p__Ascomycota;c__Dothideomycetes;o__Pleosporales;f__Teichosporaceae;g__Teichospora;s__unidentified |
| OTU311 | k__Fungi;p__Ascomycota;c__Dothideomycetes;o__Pleosporales;f__Teichosporaceae;g__unidentified;s__unidentified |
| OTU657 | k__Fungi;p__Ascomycota;c__Dothideomycetes;o__Pleosporales;f__Thyridariaceae;g__Roussoella;s__Roussoella_elaeicola |
| OTU770 | k__Fungi;p__Ascomycota;c__Dothideomycetes;o__Pleosporales;f__Thyridariaceae;g__Roussoella;s__Roussoella_neopustulans |
| OTU362 | k__Fungi;p__Ascomycota;c__Dothideomycetes;o__Pleosporales;f__Thyridariaceae;g__Roussoella;s__Roussoella_siamensis |
| OTU889 | k__Fungi;p__Ascomycota;c__Dothideomycetes;o__Pleosporales;f__Thyridariaceae;g__Roussoella;s__Roussoella_solani |
| OTU940 | k__Fungi;p__Ascomycota;c__Dothideomycetes;o__Pleosporales;f__Thyridariaceae;g__Roussoella;s__Roussoella_tuberculata |
| OTU386 | k__Fungi;p__Ascomycota;c__Dothideomycetes;o__Pleosporales;f__Thyridariaceae;g__Roussoella;s__unidentified |
| OTU1331 | k__Fungi;p__Ascomycota;c__Dothideomycetes;o__Pleosporales;f__Thyridariaceae;g__unidentified;s__unidentified |
| OTU644 | k__Fungi;p__Ascomycota;c__Dothideomycetes;o__Pleosporales;f__Torulaceae;g__Torula;s__unidentified |
| OTU13 | k__Fungi;p__Ascomycota;c__Dothideomycetes;o__Pleosporales;f__unidentified;g__unidentified;s__unidentified |
| OTU1257 | k__Fungi;p__Ascomycota;c__Dothideomycetes;o__Tubeufiales;f__Tubeufiaceae;g__Chlamydotubeufia;s__Chlamydotubeufia_khunkornensis |
| OTU932 | k__Fungi;p__Ascomycota;c__Dothideomycetes;o__Tubeufiales;f__Tubeufiaceae;g__Helicosporium;s__Helicosporium_gracile |
| OTU6 | k__Fungi;p__Ascomycota;c__Dothideomycetes;o__Tubeufiales;f__Tubeufiaceae;g__unidentified;s__unidentified |
| OTU103 | k__Fungi;p__Ascomycota;c__Dothideomycetes;o__unidentified;f__unidentified;g__unidentified;s__unidentified |
| OTU277 | k__Fungi;p__Ascomycota;c__Dothideomycetes;o__unidentified;f__unidentified;g__unidentified;s__unidentified |
| OTU656 | k__Fungi;p__Ascomycota;c__Dothideomycetes;o__unidentified;f__unidentified;g__unidentified;s__unidentified |
| OTU885 | k__Fungi;p__Ascomycota;c__Dothideomycetes;o__Valsariales;f__Valsariaceae;g__Valsaria;s__Valsaria_insitiva |
| OTU645 | k__Fungi;p__Ascomycota;c__Dothideomycetes;o__Venturiales;f__Sympoventuriaceae;g__Ochroconis;s__Ochroconis_cordanae |
| OTU1043 | k__Fungi;p__Ascomycota;c__Dothideomycetes;o__Venturiales;f__Sympoventuriaceae;g__Ochroconis;s__Ochroconis_tshawytschae |
| OTU418 | k__Fungi;p__Ascomycota;c__Dothideomycetes;o__Venturiales;f__Sympoventuriaceae;g__Ochroconis;s__unidentified |
| OTU151 | k__Fungi;p__Ascomycota;c__Dothideomycetes;o__Venturiales;f__Sympoventuriaceae;g__Scolecobasidium;s__Scolecobasidium_constrictum |
| OTU1300 | k__Fungi;p__Ascomycota;c__Dothideomycetes;o__Venturiales;f__Sympoventuriaceae;g__Troposporella;s__Troposporella_olivacea |
| OTU1309 | k__Fungi;p__Ascomycota;c__Dothideomycetes;o__Venturiales;f__Venturiales_fam_Incertae_sedis;g__Cylindrosympodioides;s__Cylindrosympodioides_brabejum |
| OTU837 | k__Fungi;p__Ascomycota;c__Eurotiomycetes;o__Chaetothyriales;f__Chaetothyriales_fam_Incertae_sedis;g__Strelitziana;s__Strelitziana_africana |
| OTU1316 | k__Fungi;p__Ascomycota;c__Eurotiomycetes;o__Chaetothyriales;f__Cyphellophoraceae;g__Cyphellophora;s__Cyphellophora_eucalypti |
| OTU735 | k__Fungi;p__Ascomycota;c__Eurotiomycetes;o__Chaetothyriales;f__Cyphellophoraceae;g__Cyphellophora;s__Cyphellophora_gamsii |
| OTU1066 | k__Fungi;p__Ascomycota;c__Eurotiomycetes;o__Chaetothyriales;f__Cyphellophoraceae;g__Cyphellophora;s__Cyphellophora_phyllostachydis |
| OTU1029 | k__Fungi;p__Ascomycota;c__Eurotiomycetes;o__Chaetothyriales;f__Cyphellophoraceae;g__Cyphellophora;s__Cyphellophora_pluriseptata |
| OTU869 | k__Fungi;p__Ascomycota;c__Eurotiomycetes;o__Chaetothyriales;f__Cyphellophoraceae;g__Cyphellophora;s__Cyphellophora_vermispora |
| OTU1013 | k__Fungi;p__Ascomycota;c__Eurotiomycetes;o__Chaetothyriales;f__Cyphellophoraceae;g__Cyphellophora;s__unidentified |
| OTU462 | k__Fungi;p__Ascomycota;c__Eurotiomycetes;o__Chaetothyriales;f__Herpotrichiellaceae;g__Capronia;s__Capronia_semi-immersa |
| OTU722 | k__Fungi;p__Ascomycota;c__Eurotiomycetes;o__Chaetothyriales;f__Herpotrichiellaceae;g__Capronia;s__unidentified |
| OTU493 | k__Fungi;p__Ascomycota;c__Eurotiomycetes;o__Chaetothyriales;f__Herpotrichiellaceae;g__Cladophialophora;s__Cladophialophora_immunda |
| OTU91 | k__Fungi;p__Ascomycota;c__Eurotiomycetes;o__Chaetothyriales;f__Herpotrichiellaceae;g__Cladophialophora;s__Cladophialophora_psammophila |
| OTU57 | k__Fungi;p__Ascomycota;c__Eurotiomycetes;o__Chaetothyriales;f__Herpotrichiellaceae;g__Cladophialophora;s__unidentified |
| OTU232 | k__Fungi;p__Ascomycota;c__Eurotiomycetes;o__Chaetothyriales;f__Herpotrichiellaceae;g__Cladophialophora;s__unidentified |
| OTU606 | k__Fungi;p__Ascomycota;c__Eurotiomycetes;o__Chaetothyriales;f__Herpotrichiellaceae;g__Cladophialophora;s__unidentified |
| OTU717 | k__Fungi;p__Ascomycota;c__Eurotiomycetes;o__Chaetothyriales;f__Herpotrichiellaceae;g__Coniosporium;s__unidentified |
| OTU1028 | k__Fungi;p__Ascomycota;c__Eurotiomycetes;o__Chaetothyriales;f__Herpotrichiellaceae;g__Exophiala;s__Exophiala_alcalophila |
| OTU784 | k__Fungi;p__Ascomycota;c__Eurotiomycetes;o__Chaetothyriales;f__Herpotrichiellaceae;g__Exophiala;s__Exophiala_cancerae |
| OTU155 | k__Fungi;p__Ascomycota;c__Eurotiomycetes;o__Chaetothyriales;f__Herpotrichiellaceae;g__Exophiala;s__Exophiala_equina |
| OTU163 | k__Fungi;p__Ascomycota;c__Eurotiomycetes;o__Chaetothyriales;f__Herpotrichiellaceae;g__Exophiala;s__Exophiala_halophila |
| OTU1054 | k__Fungi;p__Ascomycota;c__Eurotiomycetes;o__Chaetothyriales;f__Herpotrichiellaceae;g__Exophiala;s__Exophiala_mesophila |
| OTU1134 | k__Fungi;p__Ascomycota;c__Eurotiomycetes;o__Chaetothyriales;f__Herpotrichiellaceae;g__Exophiala;s__Exophiala_moniliae |
| OTU75 | k__Fungi;p__Ascomycota;c__Eurotiomycetes;o__Chaetothyriales;f__Herpotrichiellaceae;g__Exophiala;s__Exophiala_nishimurae |
| OTU34 | k__Fungi;p__Ascomycota;c__Eurotiomycetes;o__Chaetothyriales;f__Herpotrichiellaceae;g__Exophiala;s__Exophiala_pisciphila |
| OTU723 | k__Fungi;p__Ascomycota;c__Eurotiomycetes;o__Chaetothyriales;f__Herpotrichiellaceae;g__Exophiala;s__Exophiala_xenobiotica |
| OTU525 | k__Fungi;p__Ascomycota;c__Eurotiomycetes;o__Chaetothyriales;f__Herpotrichiellaceae;g__Exophiala;s__unidentified |
| OTU652 | k__Fungi;p__Ascomycota;c__Eurotiomycetes;o__Chaetothyriales;f__Herpotrichiellaceae;g__Fonsecaea;s__unidentified |
| OTU1139 | k__Fungi;p__Ascomycota;c__Eurotiomycetes;o__Chaetothyriales;f__Herpotrichiellaceae;g__Minimelanolocus;s__unidentified |
| OTU1027 | k__Fungi;p__Ascomycota;c__Eurotiomycetes;o__Chaetothyriales;f__Herpotrichiellaceae;g__Phialophora;s__Phialophora_geniculata |
| OTU56 | k__Fungi;p__Ascomycota;c__Eurotiomycetes;o__Chaetothyriales;f__Herpotrichiellaceae;g__unidentified;s__unidentified |
| OTU319 | k__Fungi;p__Ascomycota;c__Eurotiomycetes;o__Chaetothyriales;f__Herpotrichiellaceae;g__unidentified;s__unidentified |
| OTU41 | k__Fungi;p__Ascomycota;c__Eurotiomycetes;o__Chaetothyriales;f__Herpotrichiellaceae;g__Veronaea;s__Veronaea_compacta |
| OTU810 | k__Fungi;p__Ascomycota;c__Eurotiomycetes;o__Chaetothyriales;f__Herpotrichiellaceae;g__Veronaea;s__Veronaea_japonica |
| OTU595 | k__Fungi;p__Ascomycota;c__Eurotiomycetes;o__Chaetothyriales;f__Trichomeriaceae;g__Trichomerium;s__Trichomerium_eucalypti |
| OTU50 | k__Fungi;p__Ascomycota;c__Eurotiomycetes;o__Chaetothyriales;f__unidentified;g__unidentified;s__unidentified |
| OTU280 | k__Fungi;p__Ascomycota;c__Eurotiomycetes;o__Chaetothyriales;f__unidentified;g__unidentified;s__unidentified |
| OTU306 | k__Fungi;p__Ascomycota;c__Eurotiomycetes;o__Chaetothyriales;f__unidentified;g__unidentified;s__unidentified |
| OTU512 | k__Fungi;p__Ascomycota;c__Eurotiomycetes;o__Chaetothyriales;f__unidentified;g__unidentified;s__unidentified |
| OTU533 | k__Fungi;p__Ascomycota;c__Eurotiomycetes;o__Chaetothyriales;f__unidentified;g__unidentified;s__unidentified |
| OTU754 | k__Fungi;p__Ascomycota;c__Eurotiomycetes;o__Chaetothyriales;f__unidentified;g__unidentified;s__unidentified |
| OTU1237 | k__Fungi;p__Ascomycota;c__Eurotiomycetes;o__Eurotiales;f__Aspergillaceae;g__Aspergillus;s__Aspergillus_aculeatus |
| OTU577 | k__Fungi;p__Ascomycota;c__Eurotiomycetes;o__Eurotiales;f__Aspergillaceae;g__Aspergillus;s__Aspergillus_canadensis |
| OTU898 | k__Fungi;p__Ascomycota;c__Eurotiomycetes;o__Eurotiales;f__Aspergillaceae;g__Aspergillus;s__Aspergillus_carbonarius |
| OTU762 | k__Fungi;p__Ascomycota;c__Eurotiomycetes;o__Eurotiales;f__Aspergillaceae;g__Aspergillus;s__Aspergillus_citocrescens |
| OTU655 | k__Fungi;p__Ascomycota;c__Eurotiomycetes;o__Eurotiales;f__Aspergillaceae;g__Aspergillus;s__Aspergillus_citrisporus |
| OTU532 | k__Fungi;p__Ascomycota;c__Eurotiomycetes;o__Eurotiales;f__Aspergillaceae;g__Aspergillus;s__Aspergillus_clavatonanicus |
| OTU801 | k__Fungi;p__Ascomycota;c__Eurotiomycetes;o__Eurotiales;f__Aspergillaceae;g__Aspergillus;s__Aspergillus_conjunctus |
| OTU1123 | k__Fungi;p__Ascomycota;c__Eurotiomycetes;o__Eurotiales;f__Aspergillaceae;g__Aspergillus;s__Aspergillus_gorakhpurensis |
| OTU790 | k__Fungi;p__Ascomycota;c__Eurotiomycetes;o__Eurotiales;f__Aspergillaceae;g__Aspergillus;s__Aspergillus_granulosus |
| OTU1185 | k__Fungi;p__Ascomycota;c__Eurotiomycetes;o__Eurotiales;f__Aspergillaceae;g__Aspergillus;s__Aspergillus_halophilicus |
| OTU117 | k__Fungi;p__Ascomycota;c__Eurotiomycetes;o__Eurotiales;f__Aspergillaceae;g__Aspergillus;s__Aspergillus_inflatus |
| OTU1057 | k__Fungi;p__Ascomycota;c__Eurotiomycetes;o__Eurotiales;f__Aspergillaceae;g__Aspergillus;s__Aspergillus_insolitus |
| OTU775 | k__Fungi;p__Ascomycota;c__Eurotiomycetes;o__Eurotiales;f__Aspergillaceae;g__Aspergillus;s__Aspergillus_janus |
| OTU1261 | k__Fungi;p__Ascomycota;c__Eurotiomycetes;o__Eurotiales;f__Aspergillaceae;g__Aspergillus;s__Aspergillus_ochraceus |
| OTU231 | k__Fungi;p__Ascomycota;c__Eurotiomycetes;o__Eurotiales;f__Aspergillaceae;g__Aspergillus;s__Aspergillus_penicillioides |
| OTU949 | k__Fungi;p__Ascomycota;c__Eurotiomycetes;o__Eurotiales;f__Aspergillaceae;g__Aspergillus;s__Aspergillus_restrictus |
| OTU93 | k__Fungi;p__Ascomycota;c__Eurotiomycetes;o__Eurotiales;f__Aspergillaceae;g__Aspergillus;s__Aspergillus_robustus |
| OTU1074 | k__Fungi;p__Ascomycota;c__Eurotiomycetes;o__Eurotiales;f__Aspergillaceae;g__Aspergillus;s__Aspergillus_spelunceus |
| OTU979 | k__Fungi;p__Ascomycota;c__Eurotiomycetes;o__Eurotiales;f__Aspergillaceae;g__Aspergillus;s__Aspergillus_stromatoides |
| OTU1126 | k__Fungi;p__Ascomycota;c__Eurotiomycetes;o__Eurotiales;f__Aspergillaceae;g__Aspergillus;s__Aspergillus_subramanianii |
| OTU835 | k__Fungi;p__Ascomycota;c__Eurotiomycetes;o__Eurotiales;f__Aspergillaceae;g__Aspergillus;s__Aspergillus_tardus |
| OTU672 | k__Fungi;p__Ascomycota;c__Eurotiomycetes;o__Eurotiales;f__Aspergillaceae;g__Aspergillus;s__Aspergillus_wentii |
| OTU834 | k__Fungi;p__Ascomycota;c__Eurotiomycetes;o__Eurotiales;f__Aspergillaceae;g__Aspergillus;s__unidentified |
| OTU867 | k__Fungi;p__Ascomycota;c__Eurotiomycetes;o__Eurotiales;f__Aspergillaceae;g__Penicillium;s__Penicillium_adametzii |
| OTU782 | k__Fungi;p__Ascomycota;c__Eurotiomycetes;o__Eurotiales;f__Aspergillaceae;g__Penicillium;s__Penicillium_adametzioides |
| OTU912 | k__Fungi;p__Ascomycota;c__Eurotiomycetes;o__Eurotiales;f__Aspergillaceae;g__Penicillium;s__Penicillium_angulare |
| OTU626 | k__Fungi;p__Ascomycota;c__Eurotiomycetes;o__Eurotiales;f__Aspergillaceae;g__Penicillium;s__Penicillium_arianeae |
| OTU583 | k__Fungi;p__Ascomycota;c__Eurotiomycetes;o__Eurotiales;f__Aspergillaceae;g__Penicillium;s__Penicillium_atrofulvum |
| OTU289 | k__Fungi;p__Ascomycota;c__Eurotiomycetes;o__Eurotiales;f__Aspergillaceae;g__Penicillium;s__Penicillium_bovifimosum |
| OTU707 | k__Fungi;p__Ascomycota;c__Eurotiomycetes;o__Eurotiales;f__Aspergillaceae;g__Penicillium;s__Penicillium_brevicompactum |
| OTU892 | k__Fungi;p__Ascomycota;c__Eurotiomycetes;o__Eurotiales;f__Aspergillaceae;g__Penicillium;s__Penicillium_brunneoconidiatum |
| OTU1116 | k__Fungi;p__Ascomycota;c__Eurotiomycetes;o__Eurotiales;f__Aspergillaceae;g__Penicillium;s__Penicillium_camemberti |
| OTU351 | k__Fungi;p__Ascomycota;c__Eurotiomycetes;o__Eurotiales;f__Aspergillaceae;g__Penicillium;s__Penicillium_catalonicum |
| OTU464 | k__Fungi;p__Ascomycota;c__Eurotiomycetes;o__Eurotiales;f__Aspergillaceae;g__Penicillium;s__Penicillium_catenatum |
| OTU1275 | k__Fungi;p__Ascomycota;c__Eurotiomycetes;o__Eurotiales;f__Aspergillaceae;g__Penicillium;s__Penicillium_cinerascens |
| OTU861 | k__Fungi;p__Ascomycota;c__Eurotiomycetes;o__Eurotiales;f__Aspergillaceae;g__Penicillium;s__Penicillium_coffeae |
| OTU214 | k__Fungi;p__Ascomycota;c__Eurotiomycetes;o__Eurotiales;f__Aspergillaceae;g__Penicillium;s__Penicillium_cryptum |
| OTU1206 | k__Fungi;p__Ascomycota;c__Eurotiomycetes;o__Eurotiales;f__Aspergillaceae;g__Penicillium;s__Penicillium_daejeonium |
| OTU678 | k__Fungi;p__Ascomycota;c__Eurotiomycetes;o__Eurotiales;f__Aspergillaceae;g__Penicillium;s__Penicillium_decumbens |
| OTU443 | k__Fungi;p__Ascomycota;c__Eurotiomycetes;o__Eurotiales;f__Aspergillaceae;g__Penicillium;s__Penicillium_dodgei |
| OTU1278 | k__Fungi;p__Ascomycota;c__Eurotiomycetes;o__Eurotiales;f__Aspergillaceae;g__Penicillium;s__Penicillium_griseolum |
| OTU1315 | k__Fungi;p__Ascomycota;c__Eurotiomycetes;o__Eurotiales;f__Aspergillaceae;g__Penicillium;s__Penicillium_herquei |
| OTU921 | k__Fungi;p__Ascomycota;c__Eurotiomycetes;o__Eurotiales;f__Aspergillaceae;g__Penicillium;s__Penicillium_improvisum |
| OTU1169 | k__Fungi;p__Ascomycota;c__Eurotiomycetes;o__Eurotiales;f__Aspergillaceae;g__Penicillium;s__Penicillium_lineatum |
| OTU1062 | k__Fungi;p__Ascomycota;c__Eurotiomycetes;o__Eurotiales;f__Aspergillaceae;g__Penicillium;s__Penicillium_megasporum |
| OTU905 | k__Fungi;p__Ascomycota;c__Eurotiomycetes;o__Eurotiales;f__Aspergillaceae;g__Penicillium;s__Penicillium_miczynskii |
| OTU446 | k__Fungi;p__Ascomycota;c__Eurotiomycetes;o__Eurotiales;f__Aspergillaceae;g__Penicillium;s__Penicillium_ovatum |
| OTU823 | k__Fungi;p__Ascomycota;c__Eurotiomycetes;o__Eurotiales;f__Aspergillaceae;g__Penicillium;s__Penicillium_paxilli |
| OTU262 | k__Fungi;p__Ascomycota;c__Eurotiomycetes;o__Eurotiales;f__Aspergillaceae;g__Penicillium;s__Penicillium_porphyreum |
| OTU983 | k__Fungi;p__Ascomycota;c__Eurotiomycetes;o__Eurotiales;f__Aspergillaceae;g__Penicillium;s__Penicillium_ranomafanaense |
| OTU1339 | k__Fungi;p__Ascomycota;c__Eurotiomycetes;o__Eurotiales;f__Aspergillaceae;g__Penicillium;s__Penicillium_sacculum |
| OTU565 | k__Fungi;p__Ascomycota;c__Eurotiomycetes;o__Eurotiales;f__Aspergillaceae;g__Penicillium;s__Penicillium_salamorum |
| OTU792 | k__Fungi;p__Ascomycota;c__Eurotiomycetes;o__Eurotiales;f__Aspergillaceae;g__Penicillium;s__Penicillium_saturniforme |
| OTU449 | k__Fungi;p__Ascomycota;c__Eurotiomycetes;o__Eurotiales;f__Aspergillaceae;g__Penicillium;s__Penicillium_sclerotiorum |
| OTU1075 | k__Fungi;p__Ascomycota;c__Eurotiomycetes;o__Eurotiales;f__Aspergillaceae;g__Penicillium;s__Penicillium_vancouverense |
| OTU794 | k__Fungi;p__Ascomycota;c__Eurotiomycetes;o__Eurotiales;f__Aspergillaceae;g__Penicillium;s__Penicillium_virgatum |
| OTU147 | k__Fungi;p__Ascomycota;c__Eurotiomycetes;o__Eurotiales;f__Aspergillaceae;g__Penicillium;s__unidentified |
| OTU561 | k__Fungi;p__Ascomycota;c__Eurotiomycetes;o__Eurotiales;f__Aspergillaceae;g__Penicillium;s__unidentified |
| OTU179 | k__Fungi;p__Ascomycota;c__Eurotiomycetes;o__Eurotiales;f__Aspergillaceae;g__unidentified;s__unidentified |
| OTU708 | k__Fungi;p__Ascomycota;c__Eurotiomycetes;o__Eurotiales;f__Aspergillaceae;g__Xeromyces;s__Xeromyces_bisporus |
| OTU166 | k__Fungi;p__Ascomycota;c__Eurotiomycetes;o__Eurotiales;f__Elaphomycetaceae;g__Elaphomyces;s__Elaphomyces_guangdongensis |
| OTU685 | k__Fungi;p__Ascomycota;c__Eurotiomycetes;o__Eurotiales;f__Elaphomycetaceae;g__Elaphomyces;s__Elaphomyces_muricatus |
| OTU825 | k__Fungi;p__Ascomycota;c__Eurotiomycetes;o__Eurotiales;f__Elaphomycetaceae;g__Elaphomyces;s__unidentified |
| OTU1034 | k__Fungi;p__Ascomycota;c__Eurotiomycetes;o__Eurotiales;f__Elaphomycetaceae;g__unidentified;s__unidentified |
| OTU712 | k__Fungi;p__Ascomycota;c__Eurotiomycetes;o__Eurotiales;f__Trichocomaceae;g__Rasamsonia;s__Rasamsonia_byssochlamydoides |
| OTU965 | k__Fungi;p__Ascomycota;c__Eurotiomycetes;o__Eurotiales;f__Trichocomaceae;g__Rasamsonia;s__Rasamsonia_emersonii |
| OTU607 | k__Fungi;p__Ascomycota;c__Eurotiomycetes;o__Eurotiales;f__Trichocomaceae;g__Rasamsonia;s__unidentified |
| OTU1295 | k__Fungi;p__Ascomycota;c__Eurotiomycetes;o__Eurotiales;f__Trichocomaceae;g__Sagenomella;s__Sagenomella_diversispora |
| OTU632 | k__Fungi;p__Ascomycota;c__Eurotiomycetes;o__Eurotiales;f__Trichocomaceae;g__Sagenomella;s__Sagenomella_humicola |
| OTU71 | k__Fungi;p__Ascomycota;c__Eurotiomycetes;o__Eurotiales;f__Trichocomaceae;g__Sagenomella;s__Sagenomella_striatispora |
| OTU5 | k__Fungi;p__Ascomycota;c__Eurotiomycetes;o__Eurotiales;f__Trichocomaceae;g__Talaromyces;s__Talaromyces_aculeatus |
| OTU52 | k__Fungi;p__Ascomycota;c__Eurotiomycetes;o__Eurotiales;f__Trichocomaceae;g__Talaromyces;s__Talaromyces_albobiverticillius |
| OTU1100 | k__Fungi;p__Ascomycota;c__Eurotiomycetes;o__Eurotiales;f__Trichocomaceae;g__Talaromyces;s__Talaromyces_euchlorocarpius |
| OTU246 | k__Fungi;p__Ascomycota;c__Eurotiomycetes;o__Eurotiales;f__Trichocomaceae;g__Talaromyces;s__Talaromyces_infraolivaceus |
| OTU942 | k__Fungi;p__Ascomycota;c__Eurotiomycetes;o__Eurotiales;f__Trichocomaceae;g__Talaromyces;s__Talaromyces_luteus |
| OTU584 | k__Fungi;p__Ascomycota;c__Eurotiomycetes;o__Eurotiales;f__Trichocomaceae;g__Talaromyces;s__Talaromyces_mimosinus |
| OTU602 | k__Fungi;p__Ascomycota;c__Eurotiomycetes;o__Eurotiales;f__Trichocomaceae;g__Talaromyces;s__Talaromyces_minioluteus |
| OTU785 | k__Fungi;p__Ascomycota;c__Eurotiomycetes;o__Eurotiales;f__Trichocomaceae;g__Talaromyces;s__Talaromyces_purpureogenus |
| OTU1128 | k__Fungi;p__Ascomycota;c__Eurotiomycetes;o__Eurotiales;f__Trichocomaceae;g__Talaromyces;s__Talaromyces_stellenboschiensis |
| OTU692 | k__Fungi;p__Ascomycota;c__Eurotiomycetes;o__Eurotiales;f__Trichocomaceae;g__Talaromyces;s__Talaromyces_subinflatus |
| OTU1228 | k__Fungi;p__Ascomycota;c__Eurotiomycetes;o__Eurotiales;f__Trichocomaceae;g__Talaromyces;s__Talaromyces_verruculosus |
| OTU379 | k__Fungi;p__Ascomycota;c__Eurotiomycetes;o__Eurotiales;f__Trichocomaceae;g__Talaromyces;s__Talaromyces_wortmannii |
| OTU35 | k__Fungi;p__Ascomycota;c__Eurotiomycetes;o__Eurotiales;f__Trichocomaceae;g__Talaromyces;s__unidentified |
| OTU608 | k__Fungi;p__Ascomycota;c__Eurotiomycetes;o__Eurotiales;f__Trichocomaceae;g__Talaromyces;s__unidentified |
| OTU890 | k__Fungi;p__Ascomycota;c__Eurotiomycetes;o__Eurotiales;f__Trichocomaceae;g__Thermomyces;s__Thermomyces_lanuginosus |
| OTU918 | k__Fungi;p__Ascomycota;c__Eurotiomycetes;o__Eurotiales;f__Trichocomaceae;g__Thermomyces;s__Thermomyces_stellatus |
| OTU313 | k__Fungi;p__Ascomycota;c__Eurotiomycetes;o__Eurotiales;f__unidentified;g__unidentified;s__unidentified |
| OTU1095 | k__Fungi;p__Ascomycota;c__Eurotiomycetes;o__Mycocaliciales;f__Mycocaliciaceae;g__Chaenothecopsis;s__Chaenothecopsis_resinophila |
| OTU1172 | k__Fungi;p__Ascomycota;c__Eurotiomycetes;o__Onygenales;f__Gymnoascaceae;g__Leucothecium;s__Leucothecium_emdenii |
| OTU566 | k__Fungi;p__Ascomycota;c__Eurotiomycetes;o__Onygenales;f__Onygenaceae;g__Arachnotheca;s__Arachnotheca_glomerata |
| OTU938 | k__Fungi;p__Ascomycota;c__Eurotiomycetes;o__Onygenales;f__Onygenaceae;g__Auxarthron;s__Auxarthron_ostraviense |
| OTU1247 | k__Fungi;p__Ascomycota;c__Eurotiomycetes;o__Onygenales;f__Onygenaceae;g__Auxarthron;s__unidentified |
| OTU808 | k__Fungi;p__Ascomycota;c__Eurotiomycetes;o__Onygenales;f__Onygenaceae;g__Auxarthronopsis;s__unidentified |
| OTU133 | k__Fungi;p__Ascomycota;c__Eurotiomycetes;o__Onygenales;f__Onygenaceae;g__Myriodontium;s__Myriodontium_keratinophilum |
| OTU84 | k__Fungi;p__Ascomycota;c__Eurotiomycetes;o__Onygenales;f__Onygenaceae;g__Pectinotrichum;s__unidentified |
| OTU66 | k__Fungi;p__Ascomycota;c__Eurotiomycetes;o__Onygenales;f__Onygenales_fam_Incertae_sedis;g__Arthropsis;s__Arthropsis_hispanica |
| OTU407 | k__Fungi;p__Ascomycota;c__Eurotiomycetes;o__Onygenales;f__Onygenales_fam_Incertae_sedis;g__Chrysosporium;s__Chrysosporium_lobatum |
| OTU1304 | k__Fungi;p__Ascomycota;c__Eurotiomycetes;o__Onygenales;f__Onygenales_fam_Incertae_sedis;g__Spiromastix;s__unidentified |
| OTU260 | k__Fungi;p__Ascomycota;c__Eurotiomycetes;o__Phaeomoniellales;f__Phaeomoniellaceae;g__Aequabiliella;s__Aequabiliella_effusa |
| OTU1293 | k__Fungi;p__Ascomycota;c__Eurotiomycetes;o__Sclerococcales;f__Dactylosporaceae;g__Dactylospora;s__Dactylospora_stygia |
| OTU683 | k__Fungi;p__Ascomycota;c__Eurotiomycetes;o__Verrucariales;f__Verrucariaceae;g__Verrucaria;s__Verrucaria_muralis |
| OTU441 | k__Fungi;p__Ascomycota;c__Geoglossomycetes;o__Geoglossales;f__Geoglossaceae;g__Hemileucoglossum;s__unidentified |
| OTU339 | k__Fungi;p__Ascomycota;c__Geoglossomycetes;o__Geoglossales;f__Geoglossaceae;g__Trichoglossum;s__unidentified |
| OTU1 | k__Fungi;p__Ascomycota;c__GS35;o__GS35;f__unidentified;g__unidentified;s__unidentified |
| OTU989 | k__Fungi;p__Ascomycota;c__Laboulbeniomycetes;o__Pyxidiophorales;f__unidentified;g__unidentified;s__unidentified |
| OTU288 | k__Fungi;p__Ascomycota;c__Lecanoromycetes;o__Lecanorales;f__Cladoniaceae;g__Cladonia;s__Cladonia_rudis |
| OTU1065 | k__Fungi;p__Ascomycota;c__Lecanoromycetes;o__Lecanoromycetes_ord_Incertae_sedis;f__Lecanoromycetes_fam_Incertae_sedis;g__unidentified;s__unidentified |
| OTU173 | k__Fungi;p__Ascomycota;c__Lecanoromycetes;o__Ostropales;f__Stictidaceae;g__Fitzroyomyces;s__Fitzroyomyces_cyperacearum |
| OTU402 | k__Fungi;p__Ascomycota;c__Lecanoromycetes;o__Peltigerales;f__Koerberiaceae;g__Steinera;s__unidentified |
| OTU442 | k__Fungi;p__Ascomycota;c__Lecanoromycetes;o__Peltigerales;f__Pannariaceae;g__Pectenia;s__unidentified |
| OTU915 | k__Fungi;p__Ascomycota;c__Lecanoromycetes;o__Peltigerales;f__Placynthiaceae;g__Leciophysma;s__Leciophysma_saximontana |
| OTU190 | k__Fungi;p__Ascomycota;c__Lecanoromycetes;o__Pertusariales;f__Pertusariaceae;g__unidentified;s__unidentified |
| OTU1263 | k__Fungi;p__Ascomycota;c__Lecanoromycetes;o__Teloschistales;f__Teloschistaceae;g__Caloplaca;s__Caloplaca_ferruginea |
| OTU265 | k__Fungi;p__Ascomycota;c__Lecanoromycetes;o__Teloschistales;f__Teloschistaceae;g__Caloplaca;s__Caloplaca_raesaenenii |
| OTU1274 | k__Fungi;p__Ascomycota;c__Lecanoromycetes;o__Umbilicariales;f__Umbilicariaceae;g__Umbilicaria;s__Umbilicaria_aprina |
| OTU429 | k__Fungi;p__Ascomycota;c__Leotiomycetes;o__Erysiphales;f__Erysiphaceae;g__Podosphaera;s__Podosphaera_tridactyla |
| OTU877 | k__Fungi;p__Ascomycota;c__Leotiomycetes;o__Helotiales;f__Chaetomellaceae;g__Sphaerographium;s__unidentified |
| OTU674 | k__Fungi;p__Ascomycota;c__Leotiomycetes;o__Helotiales;f__Dermateaceae;g__Pezicula;s__unidentified |
| OTU536 | k__Fungi;p__Ascomycota;c__Leotiomycetes;o__Helotiales;f__Dermateaceae;g__Pseudofabraea;s__Pseudofabraea_citricarpa |
| OTU309 | k__Fungi;p__Ascomycota;c__Leotiomycetes;o__Helotiales;f__Helotiaceae;g__Hymenoscyphus;s__Hymenoscyphus_serotinus |
| OTU628 | k__Fungi;p__Ascomycota;c__Leotiomycetes;o__Helotiales;f__Helotiaceae;g__Scytalidium;s__Scytalidium_lignicola |
| OTU544 | k__Fungi;p__Ascomycota;c__Leotiomycetes;o__Helotiales;f__Helotiaceae;g__Scytalidium;s__unidentified |
| OTU1148 | k__Fungi;p__Ascomycota;c__Leotiomycetes;o__Helotiales;f__Helotiaceae;g__unidentified;s__unidentified |
| OTU1009 | k__Fungi;p__Ascomycota;c__Leotiomycetes;o__Helotiales;f__Helotiales_fam_Incertae_sedis;g__Cadophora;s__unidentified |
| OTU474 | k__Fungi;p__Ascomycota;c__Leotiomycetes;o__Helotiales;f__Helotiales_fam_Incertae_sedis;g__Chalara;s__unidentified |
| OTU591 | k__Fungi;p__Ascomycota;c__Leotiomycetes;o__Helotiales;f__Helotiales_fam_Incertae_sedis;g__Leohumicola;s__Leohumicola_incrustata |
| OTU1302 | k__Fungi;p__Ascomycota;c__Leotiomycetes;o__Helotiales;f__Helotiales_fam_Incertae_sedis;g__Leohumicola;s__Leohumicola_levissima |
| OTU927 | k__Fungi;p__Ascomycota;c__Leotiomycetes;o__Helotiales;f__Helotiales_fam_Incertae_sedis;g__Leohumicola;s__Leohumicola_minima |
| OTU580 | k__Fungi;p__Ascomycota;c__Leotiomycetes;o__Helotiales;f__Helotiales_fam_Incertae_sedis;g__Leptodontidium;s__unidentified |
| OTU654 | k__Fungi;p__Ascomycota;c__Leotiomycetes;o__Helotiales;f__Helotiales_fam_Incertae_sedis;g__Triposporium;s__Triposporium_cycadicola |
| OTU342 | k__Fungi;p__Ascomycota;c__Leotiomycetes;o__Helotiales;f__Helotiales_fam_Incertae_sedis;g__Xenopolyscytalum;s__Xenopolyscytalum_pinea |
| OTU1183 | k__Fungi;p__Ascomycota;c__Leotiomycetes;o__Helotiales;f__Hyaloscyphaceae;g__Glutinomyces;s__unidentified |
| OTU527 | k__Fungi;p__Ascomycota;c__Leotiomycetes;o__Helotiales;f__Hyaloscyphaceae;g__Hyaloscypha;s__Hyaloscypha_monodictys |
| OTU1156 | k__Fungi;p__Ascomycota;c__Leotiomycetes;o__Helotiales;f__Hyaloscyphaceae;g__Proliferodiscus;s__Proliferodiscus_earoleucus |
| OTU27 | k__Fungi;p__Ascomycota;c__Leotiomycetes;o__Helotiales;f__Hyaloscyphaceae;g__unidentified;s__unidentified |
| OTU747 | k__Fungi;p__Ascomycota;c__Leotiomycetes;o__Helotiales;f__Hyaloscyphaceae;g__unidentified;s__unidentified |
| OTU1179 | k__Fungi;p__Ascomycota;c__Leotiomycetes;o__Helotiales;f__Hyaloscyphaceae;g__unidentified;s__unidentified |
| OTU9 | k__Fungi;p__Ascomycota;c__Leotiomycetes;o__Helotiales;f__Leotiaceae;g__Alatospora;s__unidentified |
| OTU786 | k__Fungi;p__Ascomycota;c__Leotiomycetes;o__Helotiales;f__Leotiaceae;g__Gorgomyces;s__unidentified |
| OTU793 | k__Fungi;p__Ascomycota;c__Leotiomycetes;o__Helotiales;f__Leotiaceae;g__Leotia;s__Leotia_lubrica |
| OTU925 | k__Fungi;p__Ascomycota;c__Leotiomycetes;o__Helotiales;f__Myxotrichaceae;g__Byssoascus;s__Byssoascus_striatosporus |
| OTU55 | k__Fungi;p__Ascomycota;c__Leotiomycetes;o__Helotiales;f__Myxotrichaceae;g__Oidiodendron;s__Oidiodendron_chlamydosporicum |
| OTU1222 | k__Fungi;p__Ascomycota;c__Leotiomycetes;o__Helotiales;f__Myxotrichaceae;g__Oidiodendron;s__Oidiodendron_echinulatum |
| OTU658 | k__Fungi;p__Ascomycota;c__Leotiomycetes;o__Helotiales;f__Myxotrichaceae;g__Oidiodendron;s__Oidiodendron_maius |
| OTU1048 | k__Fungi;p__Ascomycota;c__Leotiomycetes;o__Helotiales;f__Myxotrichaceae;g__Oidiodendron;s__Oidiodendron_periconioides |
| OTU196 | k__Fungi;p__Ascomycota;c__Leotiomycetes;o__Helotiales;f__Myxotrichaceae;g__Oidiodendron;s__Oidiodendron_rhodogenum |
| OTU25 | k__Fungi;p__Ascomycota;c__Leotiomycetes;o__Helotiales;f__Myxotrichaceae;g__Oidiodendron;s__unidentified |
| OTU240 | k__Fungi;p__Ascomycota;c__Leotiomycetes;o__Helotiales;f__Myxotrichaceae;g__Oidiodendron;s__unidentified |
| OTU664 | k__Fungi;p__Ascomycota;c__Leotiomycetes;o__Helotiales;f__Pezizellaceae;g__Porodiplodia;s__Porodiplodia_vitis |
| OTU487 | k__Fungi;p__Ascomycota;c__Leotiomycetes;o__Helotiales;f__Rutstroemiaceae;g__Lambertella;s__Lambertella_pruni |
| OTU19 | k__Fungi;p__Ascomycota;c__Leotiomycetes;o__Helotiales;f__Sclerotiniaceae;g__Moellerodiscus;s__unidentified |
| OTU175 | k__Fungi;p__Ascomycota;c__Leotiomycetes;o__Helotiales;f__unidentified;g__unidentified;s__unidentified |
| OTU276 | k__Fungi;p__Ascomycota;c__Leotiomycetes;o__Helotiales;f__unidentified;g__unidentified;s__unidentified |
| OTU377 | k__Fungi;p__Ascomycota;c__Leotiomycetes;o__Helotiales;f__unidentified;g__unidentified;s__unidentified |
| OTU1124 | k__Fungi;p__Ascomycota;c__Leotiomycetes;o__Helotiales;f__Vibrisseaceae;g__Phialocephala;s__Phialocephala_fortinii |
| OTU1011 | k__Fungi;p__Ascomycota;c__Leotiomycetes;o__Helotiales;f__Vibrisseaceae;g__Phialocephala;s__Phialocephala_humicola |
| OTU1018 | k__Fungi;p__Ascomycota;c__Leotiomycetes;o__Helotiales;f__Vibrisseaceae;g__Phialocephala;s__unidentified |
| OTU1113 | k__Fungi;p__Ascomycota;c__Leotiomycetes;o__Phacidiales;f__Phacidiaceae;g__Phacidium;s__Phacidium_pseudophacidioides |
| OTU461 | k__Fungi;p__Ascomycota;c__Leotiomycetes;o__Thelebolales;f__Pseudeurotiaceae;g__Connersia;s__unidentified |
| OTU235 | k__Fungi;p__Ascomycota;c__Leotiomycetes;o__Thelebolales;f__Pseudeurotiaceae;g__Geomyces;s__Geomyces_auratus |
| OTU638 | k__Fungi;p__Ascomycota;c__Leotiomycetes;o__Thelebolales;f__Pseudeurotiaceae;g__Leuconeurospora;s__unidentified |
| OTU256 | k__Fungi;p__Ascomycota;c__Leotiomycetes;o__Thelebolales;f__Pseudeurotiaceae;g__Pseudeurotium;s__Pseudeurotium_ovale |
| OTU1163 | k__Fungi;p__Ascomycota;c__Leotiomycetes;o__Thelebolales;f__Pseudeurotiaceae;g__Pseudogymnoascus;s__Pseudogymnoascus_pannorum |
| OTU459 | k__Fungi;p__Ascomycota;c__Leotiomycetes;o__unidentified;f__unidentified;g__unidentified;s__unidentified |
| OTU436 | k__Fungi;p__Ascomycota;c__Orbiliomycetes;o__Orbiliales;f__Orbiliaceae;g__Arthrobotrys;s__Arthrobotrys_javanica |
| OTU237 | k__Fungi;p__Ascomycota;c__Orbiliomycetes;o__Orbiliales;f__Orbiliaceae;g__Arthrobotrys;s__Arthrobotrys_megalospora |
| OTU318 | k__Fungi;p__Ascomycota;c__Orbiliomycetes;o__Orbiliales;f__Orbiliaceae;g__Arthrobotrys;s__Arthrobotrys_musiformis |
| OTU415 | k__Fungi;p__Ascomycota;c__Orbiliomycetes;o__Orbiliales;f__Orbiliaceae;g__unidentified;s__unidentified |
| OTU902 | k__Fungi;p__Ascomycota;c__Orbiliomycetes;o__Orbiliales;f__Orbiliales_fam_Incertae_sedis;g__Microdochiella;s__Microdochiella_fusarioides |
| OTU1313 | k__Fungi;p__Ascomycota;c__Orbiliomycetes;o__Orbiliales;f__Orbiliales_fam_Incertae_sedis;g__Vermispora;s__Vermispora_fusarina |
| OTU1219 | k__Fungi;p__Ascomycota;c__Orbiliomycetes;o__Orbiliales;f__unidentified;g__unidentified;s__unidentified |
| OTU1039 | k__Fungi;p__Ascomycota;c__Pezizomycetes;o__Pezizales;f__Ascobolaceae;g__Ascobolus;s__Ascobolus_crenulatus |
| OTU881 | k__Fungi;p__Ascomycota;c__Pezizomycetes;o__Pezizales;f__Ascobolaceae;g__Ascobolus;s__unidentified |
| OTU653 | k__Fungi;p__Ascomycota;c__Pezizomycetes;o__Pezizales;f__Ascodesmidaceae;g__Ascodesmis;s__Ascodesmis_sphaerospora |
| OTU1031 | k__Fungi;p__Ascomycota;c__Pezizomycetes;o__Pezizales;f__Helvellaceae;g__Helvella;s__Helvella_elastica |
| OTU81 | k__Fungi;p__Ascomycota;c__Pezizomycetes;o__Pezizales;f__Helvellaceae;g__Helvella;s__unidentified |
| OTU494 | k__Fungi;p__Ascomycota;c__Pezizomycetes;o__Pezizales;f__Helvellaceae;g__Helvella;s__unidentified |
| OTU186 | k__Fungi;p__Ascomycota;c__Pezizomycetes;o__Pezizales;f__Pezizaceae;g__Hydnobolites;s__unidentified |
| OTU872 | k__Fungi;p__Ascomycota;c__Pezizomycetes;o__Pezizales;f__Pezizaceae;g__Hydnobolites;s__unidentified |
| OTU1227 | k__Fungi;p__Ascomycota;c__Pezizomycetes;o__Pezizales;f__Pezizaceae;g__Pachyphloeus;s__unidentified |
| OTU1120 | k__Fungi;p__Ascomycota;c__Pezizomycetes;o__Pezizales;f__Pezizaceae;g__Peziza;s__Peziza_succosa |
| OTU371 | k__Fungi;p__Ascomycota;c__Pezizomycetes;o__Pezizales;f__Pezizaceae;g__unidentified;s__unidentified |
| OTU796 | k__Fungi;p__Ascomycota;c__Pezizomycetes;o__Pezizales;f__Pyronemataceae;g__Anthracobia;s__Anthracobia_macrocystis |
| OTU134 | k__Fungi;p__Ascomycota;c__Pezizomycetes;o__Pezizales;f__Pyronemataceae;g__Humaria;s__unidentified |
| OTU895 | k__Fungi;p__Ascomycota;c__Pezizomycetes;o__Pezizales;f__Pyronemataceae;g__Pyronema;s__Pyronema_domesticum |
| OTU49 | k__Fungi;p__Ascomycota;c__Pezizomycetes;o__Pezizales;f__Pyronemataceae;g__Scutellinia;s__unidentified |
| OTU1254 | k__Fungi;p__Ascomycota;c__Pezizomycetes;o__Pezizales;f__Pyronemataceae;g__Sphaerosporella;s__unidentified |
| OTU14 | k__Fungi;p__Ascomycota;c__Pezizomycetes;o__Pezizales;f__Pyronemataceae;g__Trichophaea;s__unidentified |
| OTU813 | k__Fungi;p__Ascomycota;c__Pezizomycetes;o__Pezizales;f__Pyronemataceae;g__unidentified;s__unidentified |
| OTU839 | k__Fungi;p__Ascomycota;c__Pezizomycetes;o__Pezizales;f__Pyronemataceae;g__Warcupia;s__Warcupia_terrestris |
| OTU1334 | k__Fungi;p__Ascomycota;c__Pezizomycetes;o__Pezizales;f__Sarcoscyphaceae;g__Sarcoscypha;s__Sarcoscypha_occidentalis |
| OTU860 | k__Fungi;p__Ascomycota;c__Pezizomycetes;o__Pezizales;f__Sarcosomataceae;g__Trichaleurina;s__Trichaleurina_tenuispora |
| OTU447 | k__Fungi;p__Ascomycota;c__Pezizomycetes;o__Pezizales;f__Tuberaceae;g__Tuber;s__Tuber_bomiense |
| OTU187 | k__Fungi;p__Ascomycota;c__Pezizomycetes;o__Pezizales;f__Tuberaceae;g__Tuber;s__Tuber_lannaense |
| OTU530 | k__Fungi;p__Ascomycota;c__Pezizomycetes;o__Pezizales;f__Tuberaceae;g__Tuber;s__Tuber_magnatum |
| OTU162 | k__Fungi;p__Ascomycota;c__Pezizomycetes;o__Pezizales;f__Tuberaceae;g__Tuber;s__Tuber_thailandicum |
| OTU105 | k__Fungi;p__Ascomycota;c__Pezizomycetes;o__Pezizales;f__Tuberaceae;g__Tuber;s__unidentified |
| OTU720 | k__Fungi;p__Ascomycota;c__Pezizomycetes;o__Pezizales;f__Tuberaceae;g__Tuber;s__unidentified |
| OTU668 | k__Fungi;p__Ascomycota;c__Pezizomycetes;o__Pezizales;f__unidentified;g__unidentified;s__unidentified |
| OTU336 | k__Fungi;p__Ascomycota;c__Pezizomycotina_cls_Incertae_sedis;o__Pezizomycotina_ord_Incertae_sedis;f__Pezizomycotina_fam_Incertae_sedis;g__Amblyosporium;s__unidentified |
| OTU1166 | k__Fungi;p__Ascomycota;c__Saccharomycetes;o__Saccharomycetales;f__Debaryomycetaceae;g__Meyerozyma;s__Meyerozyma_elateridarum |
| OTU101 | k__Fungi;p__Ascomycota;c__Saccharomycetes;o__Saccharomycetales;f__Debaryomycetaceae;g__Yamadazyma;s__Yamadazyma_takamatsuzukensis |
| OTU907 | k__Fungi;p__Ascomycota;c__Saccharomycetes;o__Saccharomycetales;f__Saccharomycetaceae;g__Kazachstania;s__Kazachstania_africana |
| OTU634 | k__Fungi;p__Ascomycota;c__Saccharomycetes;o__Saccharomycetales;f__Saccharomycetaceae;g__Kazachstania;s__Kazachstania_humilis |
| OTU744 | k__Fungi;p__Ascomycota;c__Saccharomycetes;o__Saccharomycetales;f__Saccharomycetaceae;g__Kazachstania;s__Kazachstania_pintolopesii |
| OTU1150 | k__Fungi;p__Ascomycota;c__Saccharomycetes;o__Saccharomycetales;f__Saccharomycetaceae;g__Kazachstania;s__Kazachstania_slooffiae |
| OTU695 | k__Fungi;p__Ascomycota;c__Saccharomycetes;o__Saccharomycetales;f__Saccharomycetaceae;g__Kluyveromyces;s__Kluyveromyces_hubeiensis |
| OTU1325 | k__Fungi;p__Ascomycota;c__Saccharomycetes;o__Saccharomycetales;f__Saccharomycetales_fam_Incertae_sedis;g__Candida;s__Candida_powellii |
| OTU99 | k__Fungi;p__Ascomycota;c__Saccharomycetes;o__Saccharomycetales;f__Trichomonascaceae;g__Wickerhamiella;s__Wickerhamiella_pararugosa |
| OTU257 | k__Fungi;p__Ascomycota;c__Saccharomycetes;o__Saccharomycetales;f__unidentified;g__unidentified;s__unidentified |
| OTU698 | k__Fungi;p__Ascomycota;c__Sordariomycetes;o__Boliniales;f__Boliniaceae;g__Endoxyla;s__unidentified |
| OTU531 | k__Fungi;p__Ascomycota;c__Sordariomycetes;o__Boliniales;f__Boliniales_fam_Incertae_sedis;g__Rhexoacrodictys;s__Rhexoacrodictys_fimicola |
| OTU705 | k__Fungi;p__Ascomycota;c__Sordariomycetes;o__Calosphaeriales;f__Calosphaeriaceae;g__Jattaea;s__Jattaea_mookgoponga |
| OTU824 | k__Fungi;p__Ascomycota;c__Sordariomycetes;o__Calosphaeriales;f__Calosphaeriaceae;g__Jattaea;s__Jattaea_prunicola |
| OTU993 | k__Fungi;p__Ascomycota;c__Sordariomycetes;o__Calosphaeriales;f__Pleurostomataceae;g__Pleurostoma;s__Pleurostoma_ochraceum |
| OTU1025 | k__Fungi;p__Ascomycota;c__Sordariomycetes;o__Calosphaeriales;f__Pleurostomataceae;g__Pleurostoma;s__Pleurostoma_richardsiae |
| OTU704 | k__Fungi;p__Ascomycota;c__Sordariomycetes;o__Chaetosphaeriales;f__Chaetosphaeriaceae;g__Chaetosphaeria;s__Chaetosphaeria_jonesii |
| OTU321 | k__Fungi;p__Ascomycota;c__Sordariomycetes;o__Chaetosphaeriales;f__Chaetosphaeriaceae;g__Chaetosphaeria;s__unidentified |
| OTU475 | k__Fungi;p__Ascomycota;c__Sordariomycetes;o__Chaetosphaeriales;f__Chaetosphaeriaceae;g__Chloridium;s__Chloridium_aseptatum |
| OTU1006 | k__Fungi;p__Ascomycota;c__Sordariomycetes;o__Chaetosphaeriales;f__Chaetosphaeriaceae;g__Chloridium;s__unidentified |
| OTU484 | k__Fungi;p__Ascomycota;c__Sordariomycetes;o__Chaetosphaeriales;f__Chaetosphaeriaceae;g__Dictyochaeta;s__Dictyochaeta_siamensis |
| OTU887 | k__Fungi;p__Ascomycota;c__Sordariomycetes;o__Chaetosphaeriales;f__Chaetosphaeriaceae;g__Porosphaerella;s__Porosphaerella_cordanophora |
| OTU219 | k__Fungi;p__Ascomycota;c__Sordariomycetes;o__Chaetosphaeriales;f__Chaetosphaeriaceae;g__unidentified;s__unidentified |
| OTU317 | k__Fungi;p__Ascomycota;c__Sordariomycetes;o__Chaetosphaeriales;f__Chaetosphaeriaceae;g__unidentified;s__unidentified |
| OTU299 | k__Fungi;p__Ascomycota;c__Sordariomycetes;o__Chaetosphaeriales;f__unidentified;g__unidentified;s__unidentified |
| OTU953 | k__Fungi;p__Ascomycota;c__Sordariomycetes;o__Coniochaetales;f__Coniochaetaceae;g__Coniochaeta;s__Coniochaeta_decumbens |
| OTU924 | k__Fungi;p__Ascomycota;c__Sordariomycetes;o__Coniochaetales;f__Coniochaetaceae;g__Coniochaeta;s__Coniochaeta_dendrobiicola |
| OTU957 | k__Fungi;p__Ascomycota;c__Sordariomycetes;o__Coniochaetales;f__Coniochaetaceae;g__Coniochaeta;s__Coniochaeta_fasciculata |
| OTU1307 | k__Fungi;p__Ascomycota;c__Sordariomycetes;o__Coniochaetales;f__Coniochaetaceae;g__Coniochaeta;s__Coniochaeta_mutabilis |
| OTU510 | k__Fungi;p__Ascomycota;c__Sordariomycetes;o__Coniochaetales;f__Coniochaetaceae;g__Coniochaeta;s__unidentified |
| OTU980 | k__Fungi;p__Ascomycota;c__Sordariomycetes;o__Conioscyphales;f__Conioscyphaceae;g__Conioscypha;s__Conioscypha_minutispora |
| OTU582 | k__Fungi;p__Ascomycota;c__Sordariomycetes;o__Conioscyphales;f__Conioscyphaceae;g__Conioscypha;s__Conioscypha_pleiomorpha |
| OTU1084 | k__Fungi;p__Ascomycota;c__Sordariomycetes;o__Diaporthales;f__Cryphonectriaceae;g__Chrysofolia;s__Chrysofolia_colombiana |
| OTU409 | k__Fungi;p__Ascomycota;c__Sordariomycetes;o__Diaporthales;f__Cryphonectriaceae;g__unidentified;s__unidentified |
| OTU132 | k__Fungi;p__Ascomycota;c__Sordariomycetes;o__Diaporthales;f__Diaporthaceae;g__Diaporthe;s__Diaporthe_amygdali |
| OTU1318 | k__Fungi;p__Ascomycota;c__Sordariomycetes;o__Diaporthales;f__Diaporthaceae;g__Diaporthe;s__Diaporthe_aseana |
| OTU866 | k__Fungi;p__Ascomycota;c__Sordariomycetes;o__Diaporthales;f__Diaporthaceae;g__Diaporthe;s__Diaporthe_citrichinensis |
| OTU853 | k__Fungi;p__Ascomycota;c__Sordariomycetes;o__Diaporthales;f__Diaporthaceae;g__Diaporthe;s__Diaporthe_columnaris |
| OTU1213 | k__Fungi;p__Ascomycota;c__Sordariomycetes;o__Diaporthales;f__Diaporthaceae;g__Diaporthe;s__Diaporthe_helicis |
| OTU514 | k__Fungi;p__Ascomycota;c__Sordariomycetes;o__Diaporthales;f__Diaporthaceae;g__Diaporthe;s__unidentified |
| OTU956 | k__Fungi;p__Ascomycota;c__Sordariomycetes;o__Diaporthales;f__Melanconidaceae;g__Melanconiella;s__Melanconiella_ellisii |
| OTU247 | k__Fungi;p__Ascomycota;c__Sordariomycetes;o__Diaporthales;f__Schizoparmaceae;g__Coniella;s__Coniella_duckerae |
| OTU971 | k__Fungi;p__Ascomycota;c__Sordariomycetes;o__Diaporthales;f__Schizoparmaceae;g__Coniella;s__Coniella_koreana |
| OTU40 | k__Fungi;p__Ascomycota;c__Sordariomycetes;o__Diaporthales;f__unidentified;g__unidentified;s__unidentified |
| OTU496 | k__Fungi;p__Ascomycota;c__Sordariomycetes;o__Diaporthales;f__Valsaceae;g__Allocryptovalsa;s__Allocryptovalsa_polyspora |
| OTU676 | k__Fungi;p__Ascomycota;c__Sordariomycetes;o__Diaporthales;f__Valsaceae;g__Cytospora;s__Cytospora_acaciae |
| OTU241 | k__Fungi;p__Ascomycota;c__Sordariomycetes;o__Diaporthales;f__Valsaceae;g__Cytospora;s__Cytospora_myrtagena |
| OTU183 | k__Fungi;p__Ascomycota;c__Sordariomycetes;o__Diaporthales;f__Valsaceae;g__Cytospora;s__Cytospora_nitschkei |
| OTU815 | k__Fungi;p__Ascomycota;c__Sordariomycetes;o__Diaporthales;f__Valsaceae;g__Phomopsis;s__unidentified |
| OTU1160 | k__Fungi;p__Ascomycota;c__Sordariomycetes;o__Glomerellales;f__Glomerellaceae;g__Colletotrichum;s__Colletotrichum_gigasporum |
| OTU721 | k__Fungi;p__Ascomycota;c__Sordariomycetes;o__Glomerellales;f__Glomerellaceae;g__Colletotrichum;s__Colletotrichum_gloeosporioides |
| OTU1131 | k__Fungi;p__Ascomycota;c__Sordariomycetes;o__Glomerellales;f__Glomerellaceae;g__Colletotrichum;s__Colletotrichum_tofieldiae |
| OTU1125 | k__Fungi;p__Ascomycota;c__Sordariomycetes;o__Glomerellales;f__Plectosphaerellaceae;g__Acrostalagmus;s__Acrostalagmus_luteoalbus |
| OTU195 | k__Fungi;p__Ascomycota;c__Sordariomycetes;o__Glomerellales;f__Plectosphaerellaceae;g__Gibellulopsis;s__Gibellulopsis_chrysanthemi |
| OTU597 | k__Fungi;p__Ascomycota;c__Sordariomycetes;o__Glomerellales;f__Plectosphaerellaceae;g__Plectosphaerella;s__Plectosphaerella_oligotrophica |
| OTU1022 | k__Fungi;p__Ascomycota;c__Sordariomycetes;o__Glomerellales;f__Plectosphaerellaceae;g__Plectosphaerella;s__unidentified |
| OTU348 | k__Fungi;p__Ascomycota;c__Sordariomycetes;o__Glomerellales;f__Plectosphaerellaceae;g__Stachylidium;s__unidentified |
| OTU1030 | k__Fungi;p__Ascomycota;c__Sordariomycetes;o__Glomerellales;f__Plectosphaerellaceae;g__Verticillium;s__Verticillium_dahliae |
| OTU690 | k__Fungi;p__Ascomycota;c__Sordariomycetes;o__Glomerellales;f__Plectosphaerellaceae;g__Verticillium;s__Verticillium_tricorpus |
| OTU1091 | k__Fungi;p__Ascomycota;c__Sordariomycetes;o__Hypoceales;f__Catabotrydaceae;g__unidentified;s__unidentified |
| OTU1114 | k__Fungi;p__Ascomycota;c__Sordariomycetes;o__Hypocreales;f__Bionectriaceae;g__Clonostachys;s__Clonostachys_rosea |
| OTU1129 | k__Fungi;p__Ascomycota;c__Sordariomycetes;o__Hypocreales;f__Bionectriaceae;g__Gliomastix;s__unidentified |
| OTU758 | k__Fungi;p__Ascomycota;c__Sordariomycetes;o__Hypocreales;f__Bionectriaceae;g__Ovicillium;s__Ovicillium_attenuatum |
| OTU8 | k__Fungi;p__Ascomycota;c__Sordariomycetes;o__Hypocreales;f__Bionectriaceae;g__Ovicillium;s__Ovicillium_oosporum |
| OTU365 | k__Fungi;p__Ascomycota;c__Sordariomycetes;o__Hypocreales;f__Bionectriaceae;g__unidentified;s__unidentified |
| OTU414 | k__Fungi;p__Ascomycota;c__Sordariomycetes;o__Hypocreales;f__Clavicipitaceae;g__Metacordyceps;s__Metacordyceps_chlamydosporia |
| OTU1328 | k__Fungi;p__Ascomycota;c__Sordariomycetes;o__Hypocreales;f__Clavicipitaceae;g__Metacordyceps;s__Metacordyceps_shibinensis |
| OTU732 | k__Fungi;p__Ascomycota;c__Sordariomycetes;o__Hypocreales;f__Clavicipitaceae;g__Metapochonia;s__Metapochonia_goniodes |
| OTU791 | k__Fungi;p__Ascomycota;c__Sordariomycetes;o__Hypocreales;f__Clavicipitaceae;g__Metapochonia;s__unidentified |
| OTU226 | k__Fungi;p__Ascomycota;c__Sordariomycetes;o__Hypocreales;f__Clavicipitaceae;g__Metarhizium;s__Metarhizium_anisopliae |
| OTU727 | k__Fungi;p__Ascomycota;c__Sordariomycetes;o__Hypocreales;f__Clavicipitaceae;g__Metarhizium;s__Metarhizium_carneum |
| OTU352 | k__Fungi;p__Ascomycota;c__Sordariomycetes;o__Hypocreales;f__Clavicipitaceae;g__Metarhizium;s__Metarhizium_flavoviride |
| OTU610 | k__Fungi;p__Ascomycota;c__Sordariomycetes;o__Hypocreales;f__Clavicipitaceae;g__Metarhizium;s__Metarhizium_marquandii |
| OTU1297 | k__Fungi;p__Ascomycota;c__Sordariomycetes;o__Hypocreales;f__Clavicipitaceae;g__Metarhizium;s__Metarhizium_pemphigi |
| OTU894 | k__Fungi;p__Ascomycota;c__Sordariomycetes;o__Hypocreales;f__Clavicipitaceae;g__Metarhizium;s__Metarhizium_rileyi |
| OTU689 | k__Fungi;p__Ascomycota;c__Sordariomycetes;o__Hypocreales;f__Clavicipitaceae;g__Metarhizium;s__unidentified |
| OTU802 | k__Fungi;p__Ascomycota;c__Sordariomycetes;o__Hypocreales;f__Clavicipitaceae;g__Paecilomyces;s__Paecilomyces_penicillatus |
| OTU818 | k__Fungi;p__Ascomycota;c__Sordariomycetes;o__Hypocreales;f__Clavicipitaceae;g__Paecilomyces;s__Paecilomyces_verrucosus |
| OTU58 | k__Fungi;p__Ascomycota;c__Sordariomycetes;o__Hypocreales;f__Clavicipitaceae;g__Paecilomyces;s__unidentified |
| OTU819 | k__Fungi;p__Ascomycota;c__Sordariomycetes;o__Hypocreales;f__Clavicipitaceae;g__Pochonia;s__Pochonia_globispora |
| OTU136 | k__Fungi;p__Ascomycota;c__Sordariomycetes;o__Hypocreales;f__Clavicipitaceae;g__Pochonia;s__unidentified |
| OTU119 | k__Fungi;p__Ascomycota;c__Sordariomycetes;o__Hypocreales;f__Clavicipitaceae;g__unidentified;s__unidentified |
| OTU799 | k__Fungi;p__Ascomycota;c__Sordariomycetes;o__Hypocreales;f__Clavicipitaceae;g__unidentified;s__unidentified |
| OTU994 | k__Fungi;p__Ascomycota;c__Sordariomycetes;o__Hypocreales;f__Cordycipitaceae;g__Beauveria;s__Beauveria_caledonica |
| OTU358 | k__Fungi;p__Ascomycota;c__Sordariomycetes;o__Hypocreales;f__Cordycipitaceae;g__Beauveria;s__Beauveria_felina |
| OTU804 | k__Fungi;p__Ascomycota;c__Sordariomycetes;o__Hypocreales;f__Cordycipitaceae;g__Beauveria;s__Beauveria_malawiensis |
| OTU301 | k__Fungi;p__Ascomycota;c__Sordariomycetes;o__Hypocreales;f__Cordycipitaceae;g__Beauveria;s__Beauveria_pseudobassiana |
| OTU112 | k__Fungi;p__Ascomycota;c__Sordariomycetes;o__Hypocreales;f__Cordycipitaceae;g__Beauveria;s__Beauveria_sungii |
| OTU1020 | k__Fungi;p__Ascomycota;c__Sordariomycetes;o__Hypocreales;f__Cordycipitaceae;g__Beauveria;s__unidentified |
| OTU633 | k__Fungi;p__Ascomycota;c__Sordariomycetes;o__Hypocreales;f__Cordycipitaceae;g__Cordyceps;s__Cordyceps_brongniartii |
| OTU1167 | k__Fungi;p__Ascomycota;c__Sordariomycetes;o__Hypocreales;f__Cordycipitaceae;g__Cordyceps;s__Cordyceps_cateniannulata |
| OTU682 | k__Fungi;p__Ascomycota;c__Sordariomycetes;o__Hypocreales;f__Cordycipitaceae;g__Cordyceps;s__Cordyceps_polyarthra |
| OTU1078 | k__Fungi;p__Ascomycota;c__Sordariomycetes;o__Hypocreales;f__Cordycipitaceae;g__Isaria;s__Isaria_fumosorosea |
| OTU1173 | k__Fungi;p__Ascomycota;c__Sordariomycetes;o__Hypocreales;f__Cordycipitaceae;g__Isaria;s__Isaria_javanica |
| OTU1264 | k__Fungi;p__Ascomycota;c__Sordariomycetes;o__Hypocreales;f__Cordycipitaceae;g__Lecanicillium;s__Lecanicillium_dimorphum |
| OTU620 | k__Fungi;p__Ascomycota;c__Sordariomycetes;o__Hypocreales;f__Cordycipitaceae;g__Lecanicillium;s__Lecanicillium_fungicola |
| OTU729 | k__Fungi;p__Ascomycota;c__Sordariomycetes;o__Hypocreales;f__Cordycipitaceae;g__Lecanicillium;s__Lecanicillium_fusisporum |
| OTU367 | k__Fungi;p__Ascomycota;c__Sordariomycetes;o__Hypocreales;f__Cordycipitaceae;g__Lecanicillium;s__Lecanicillium_kalimantanense |
| OTU122 | k__Fungi;p__Ascomycota;c__Sordariomycetes;o__Hypocreales;f__Cordycipitaceae;g__Lecanicillium;s__Lecanicillium_primulinum |
| OTU359 | k__Fungi;p__Ascomycota;c__Sordariomycetes;o__Hypocreales;f__Cordycipitaceae;g__Lecanicillium;s__Lecanicillium_psalliotae |
| OTU972 | k__Fungi;p__Ascomycota;c__Sordariomycetes;o__Hypocreales;f__Cordycipitaceae;g__Lecanicillium;s__unidentified |
| OTU636 | k__Fungi;p__Ascomycota;c__Sordariomycetes;o__Hypocreales;f__Cordycipitaceae;g__Leptobacillium;s__Leptobacillium_leptobactrum |
| OTU797 | k__Fungi;p__Ascomycota;c__Sordariomycetes;o__Hypocreales;f__Cordycipitaceae;g__Simplicillium;s__Simplicillium_cylindrosporum |
| OTU809 | k__Fungi;p__Ascomycota;c__Sordariomycetes;o__Hypocreales;f__Cordycipitaceae;g__Simplicillium;s__Simplicillium_lamellicola |
| OTU1200 | k__Fungi;p__Ascomycota;c__Sordariomycetes;o__Hypocreales;f__Cordycipitaceae;g__Simplicillium;s__unidentified |
| OTU630 | k__Fungi;p__Ascomycota;c__Sordariomycetes;o__Hypocreales;f__Cordycipitaceae;g__unidentified;s__unidentified |
| OTU833 | k__Fungi;p__Ascomycota;c__Sordariomycetes;o__Hypocreales;f__Hypocreaceae;g__Cladobotryum;s__Cladobotryum_obconicum |
| OTU279 | k__Fungi;p__Ascomycota;c__Sordariomycetes;o__Hypocreales;f__Hypocreaceae;g__Cladobotryum;s__Cladobotryum_paravirescens |
| OTU693 | k__Fungi;p__Ascomycota;c__Sordariomycetes;o__Hypocreales;f__Hypocreaceae;g__Hypomyces;s__Hypomyces_cervinus |
| OTU1188 | k__Fungi;p__Ascomycota;c__Sordariomycetes;o__Hypocreales;f__Hypocreaceae;g__Hypomyces;s__Hypomyces_chlorinigenus |
| OTU323 | k__Fungi;p__Ascomycota;c__Sordariomycetes;o__Hypocreales;f__Hypocreaceae;g__Hypomyces;s__Hypomyces_ellipsosporus |
| OTU946 | k__Fungi;p__Ascomycota;c__Sordariomycetes;o__Hypocreales;f__Hypocreaceae;g__Hypomyces;s__Hypomyces_microspermus |
| OTU7 | k__Fungi;p__Ascomycota;c__Sordariomycetes;o__Hypocreales;f__Hypocreaceae;g__Hypomyces;s__Hypomyces_mycophilus |
| OTU368 | k__Fungi;p__Ascomycota;c__Sordariomycetes;o__Hypocreales;f__Hypocreaceae;g__Hypomyces;s__Hypomyces_tubariicola |
| OTU180 | k__Fungi;p__Ascomycota;c__Sordariomycetes;o__Hypocreales;f__Hypocreaceae;g__Hypomyces;s__unidentified |
| OTU850 | k__Fungi;p__Ascomycota;c__Sordariomycetes;o__Hypocreales;f__Hypocreaceae;g__Hypomyces;s__unidentified |
| OTU396 | k__Fungi;p__Ascomycota;c__Sordariomycetes;o__Hypocreales;f__Hypocreaceae;g__Monocillium;s__Monocillium_dimorphosporum |
| OTU341 | k__Fungi;p__Ascomycota;c__Sordariomycetes;o__Hypocreales;f__Hypocreaceae;g__Monocillium;s__Monocillium_griseo-ochraceum |
| OTU640 | k__Fungi;p__Ascomycota;c__Sordariomycetes;o__Hypocreales;f__Hypocreaceae;g__Monocillium;s__Monocillium_indicum |
| OTU229 | k__Fungi;p__Ascomycota;c__Sordariomycetes;o__Hypocreales;f__Hypocreaceae;g__Monocillium;s__Monocillium_mucidum |
| OTU364 | k__Fungi;p__Ascomycota;c__Sordariomycetes;o__Hypocreales;f__Hypocreaceae;g__Monocillium;s__unidentified |
| OTU714 | k__Fungi;p__Ascomycota;c__Sordariomycetes;o__Hypocreales;f__Hypocreaceae;g__Protocrea;s__Protocrea_pallida |
| OTU1069 | k__Fungi;p__Ascomycota;c__Sordariomycetes;o__Hypocreales;f__Hypocreaceae;g__Sepedonium;s__Sepedonium_ampullosporum |
| OTU1286 | k__Fungi;p__Ascomycota;c__Sordariomycetes;o__Hypocreales;f__Hypocreaceae;g__Sepedonium;s__Sepedonium_laevigatum |
| OTU302 | k__Fungi;p__Ascomycota;c__Sordariomycetes;o__Hypocreales;f__Hypocreaceae;g__Sphaerostilbella;s__unidentified |
| OTU699 | k__Fungi;p__Ascomycota;c__Sordariomycetes;o__Hypocreales;f__Hypocreaceae;g__Trichoderma;s__Trichoderma_aerugineum |
| OTU167 | k__Fungi;p__Ascomycota;c__Sordariomycetes;o__Hypocreales;f__Hypocreaceae;g__Trichoderma;s__Trichoderma_aggressivum |
| OTU764 | k__Fungi;p__Ascomycota;c__Sordariomycetes;o__Hypocreales;f__Hypocreaceae;g__Trichoderma;s__Trichoderma_britdaniae |
| OTU773 | k__Fungi;p__Ascomycota;c__Sordariomycetes;o__Hypocreales;f__Hypocreaceae;g__Trichoderma;s__Trichoderma_caerulescens |
| OTU507 | k__Fungi;p__Ascomycota;c__Sordariomycetes;o__Hypocreales;f__Hypocreaceae;g__Trichoderma;s__Trichoderma_cerinum |
| OTU788 | k__Fungi;p__Ascomycota;c__Sordariomycetes;o__Hypocreales;f__Hypocreaceae;g__Trichoderma;s__Trichoderma_compactum |
| OTU480 | k__Fungi;p__Ascomycota;c__Sordariomycetes;o__Hypocreales;f__Hypocreaceae;g__Trichoderma;s__Trichoderma_cremeum |
| OTU966 | k__Fungi;p__Ascomycota;c__Sordariomycetes;o__Hypocreales;f__Hypocreaceae;g__Trichoderma;s__Trichoderma_delicatulum |
| OTU621 | k__Fungi;p__Ascomycota;c__Sordariomycetes;o__Hypocreales;f__Hypocreaceae;g__Trichoderma;s__Trichoderma_deliquescens |
| OTU16 | k__Fungi;p__Ascomycota;c__Sordariomycetes;o__Hypocreales;f__Hypocreaceae;g__Trichoderma;s__Trichoderma_fertile |
| OTU39 | k__Fungi;p__Ascomycota;c__Sordariomycetes;o__Hypocreales;f__Hypocreaceae;g__Trichoderma;s__Trichoderma_harzianum |
| OTU741 | k__Fungi;p__Ascomycota;c__Sordariomycetes;o__Hypocreales;f__Hypocreaceae;g__Trichoderma;s__Trichoderma_longibrachiatum |
| OTU909 | k__Fungi;p__Ascomycota;c__Sordariomycetes;o__Hypocreales;f__Hypocreaceae;g__Trichoderma;s__Trichoderma_parestonicum |
| OTU200 | k__Fungi;p__Ascomycota;c__Sordariomycetes;o__Hypocreales;f__Hypocreaceae;g__Trichoderma;s__Trichoderma_paucisporum |
| OTU1143 | k__Fungi;p__Ascomycota;c__Sordariomycetes;o__Hypocreales;f__Hypocreaceae;g__Trichoderma;s__Trichoderma_phyllostachydis |
| OTU1262 | k__Fungi;p__Ascomycota;c__Sordariomycetes;o__Hypocreales;f__Hypocreaceae;g__Trichoderma;s__Trichoderma_piluliferum |
| OTU1322 | k__Fungi;p__Ascomycota;c__Sordariomycetes;o__Hypocreales;f__Hypocreaceae;g__Trichoderma;s__Trichoderma_pseudostramineum |
| OTU120 | k__Fungi;p__Ascomycota;c__Sordariomycetes;o__Hypocreales;f__Hypocreaceae;g__Trichoderma;s__Trichoderma_rifaii |
| OTU85 | k__Fungi;p__Ascomycota;c__Sordariomycetes;o__Hypocreales;f__Hypocreaceae;g__Trichoderma;s__Trichoderma_scalesiae |
| OTU1296 | k__Fungi;p__Ascomycota;c__Sordariomycetes;o__Hypocreales;f__Hypocreaceae;g__Trichoderma;s__Trichoderma_stellatum |
| OTU477 | k__Fungi;p__Ascomycota;c__Sordariomycetes;o__Hypocreales;f__Hypocreaceae;g__Trichoderma;s__Trichoderma_stilbohypoxyli |
| OTU334 | k__Fungi;p__Ascomycota;c__Sordariomycetes;o__Hypocreales;f__Hypocreaceae;g__Trichoderma;s__Trichoderma_surrotundum |
| OTU1231 | k__Fungi;p__Ascomycota;c__Sordariomycetes;o__Hypocreales;f__Hypocreaceae;g__Trichoderma;s__Trichoderma_tawa |
| OTU843 | k__Fungi;p__Ascomycota;c__Sordariomycetes;o__Hypocreales;f__Hypocreaceae;g__Trichoderma;s__Trichoderma_turrialbense |
| OTU526 | k__Fungi;p__Ascomycota;c__Sordariomycetes;o__Hypocreales;f__Hypocreaceae;g__Trichoderma;s__unidentified |
| OTU572 | k__Fungi;p__Ascomycota;c__Sordariomycetes;o__Hypocreales;f__Hypocreaceae;g__unidentified;s__unidentified |
| OTU492 | k__Fungi;p__Ascomycota;c__Sordariomycetes;o__Hypocreales;f__Hypocreales_fam_Incertae_sedis;g__Acremonium;s__Acremonium_cavaraeanum |
| OTU354 | k__Fungi;p__Ascomycota;c__Sordariomycetes;o__Hypocreales;f__Hypocreales_fam_Incertae_sedis;g__Acremonium;s__Acremonium_charticola |
| OTU982 | k__Fungi;p__Ascomycota;c__Sordariomycetes;o__Hypocreales;f__Hypocreales_fam_Incertae_sedis;g__Acremonium;s__Acremonium_curvulum |
| OTU1310 | k__Fungi;p__Ascomycota;c__Sordariomycetes;o__Hypocreales;f__Hypocreales_fam_Incertae_sedis;g__Acremonium;s__Acremonium_exuviarum |
| OTU106 | k__Fungi;p__Ascomycota;c__Sordariomycetes;o__Hypocreales;f__Hypocreales_fam_Incertae_sedis;g__Acremonium;s__Acremonium_furcatum |
| OTU144 | k__Fungi;p__Ascomycota;c__Sordariomycetes;o__Hypocreales;f__Hypocreales_fam_Incertae_sedis;g__Acremonium;s__Acremonium_fusidioides |
| OTU864 | k__Fungi;p__Ascomycota;c__Sordariomycetes;o__Hypocreales;f__Hypocreales_fam_Incertae_sedis;g__Acremonium;s__Acremonium_lichenicola |
| OTU726 | k__Fungi;p__Ascomycota;c__Sordariomycetes;o__Hypocreales;f__Hypocreales_fam_Incertae_sedis;g__Acremonium;s__Acremonium_masseei |
| OTU1073 | k__Fungi;p__Ascomycota;c__Sordariomycetes;o__Hypocreales;f__Hypocreales_fam_Incertae_sedis;g__Acremonium;s__Acremonium_nepalense |
| OTU1007 | k__Fungi;p__Ascomycota;c__Sordariomycetes;o__Hypocreales;f__Hypocreales_fam_Incertae_sedis;g__Acremonium;s__Acremonium_persicinum |
| OTU234 | k__Fungi;p__Ascomycota;c__Sordariomycetes;o__Hypocreales;f__Hypocreales_fam_Incertae_sedis;g__Acremonium;s__Acremonium_pilosum |
| OTU829 | k__Fungi;p__Ascomycota;c__Sordariomycetes;o__Hypocreales;f__Hypocreales_fam_Incertae_sedis;g__Acremonium;s__Acremonium_polychromum |
| OTU778 | k__Fungi;p__Ascomycota;c__Sordariomycetes;o__Hypocreales;f__Hypocreales_fam_Incertae_sedis;g__Acremonium;s__Acremonium_tubakii |
| OTU109 | k__Fungi;p__Ascomycota;c__Sordariomycetes;o__Hypocreales;f__Hypocreales_fam_Incertae_sedis;g__Acremonium;s__unidentified |
| OTU694 | k__Fungi;p__Ascomycota;c__Sordariomycetes;o__Hypocreales;f__Hypocreales_fam_Incertae_sedis;g__Acremonium;s__unidentified |
| OTU1253 | k__Fungi;p__Ascomycota;c__Sordariomycetes;o__Hypocreales;f__Hypocreales_fam_Incertae_sedis;g__Fusariella;s__unidentified |
| OTU466 | k__Fungi;p__Ascomycota;c__Sordariomycetes;o__Hypocreales;f__Hypocreales_fam_Incertae_sedis;g__Gliocladium;s__unidentified |
| OTU715 | k__Fungi;p__Ascomycota;c__Sordariomycetes;o__Hypocreales;f__Hypocreales_fam_Incertae_sedis;g__Sarocladium;s__Sarocladium_kiliense |
| OTU875 | k__Fungi;p__Ascomycota;c__Sordariomycetes;o__Hypocreales;f__Hypocreales_fam_Incertae_sedis;g__unidentified;s__unidentified |
| OTU177 | k__Fungi;p__Ascomycota;c__Sordariomycetes;o__Hypocreales;f__Hypocreales_fam_Incertae_sedis;g__Ustilaginoidea;s__Ustilaginoidea_virens |
| OTU1053 | k__Fungi;p__Ascomycota;c__Sordariomycetes;o__Hypocreales;f__Nectriaceae;g__Campylocarpon;s__Campylocarpon_fasciculare |
| OTU1024 | k__Fungi;p__Ascomycota;c__Sordariomycetes;o__Hypocreales;f__Nectriaceae;g__Campylospora;s__Campylospora_leptosoma |
| OTU893 | k__Fungi;p__Ascomycota;c__Sordariomycetes;o__Hypocreales;f__Nectriaceae;g__Chaetopsina;s__Chaetopsina_fulva |
| OTU523 | k__Fungi;p__Ascomycota;c__Sordariomycetes;o__Hypocreales;f__Nectriaceae;g__Chaetopsina;s__Chaetopsina_pini |
| OTU387 | k__Fungi;p__Ascomycota;c__Sordariomycetes;o__Hypocreales;f__Nectriaceae;g__Cylindrocarpon;s__Cylindrocarpon_theobromicola |
| OTU648 | k__Fungi;p__Ascomycota;c__Sordariomycetes;o__Hypocreales;f__Nectriaceae;g__Cylindrocarpon;s__unidentified |
| OTU248 | k__Fungi;p__Ascomycota;c__Sordariomycetes;o__Hypocreales;f__Nectriaceae;g__Cylindrocladium;s__Cylindrocladium_clavatum |
| OTU728 | k__Fungi;p__Ascomycota;c__Sordariomycetes;o__Hypocreales;f__Nectriaceae;g__Cylindrocladium;s__Cylindrocladium_peruvianum |
| OTU405 | k__Fungi;p__Ascomycota;c__Sordariomycetes;o__Hypocreales;f__Nectriaceae;g__Dactylonectria;s__Dactylonectria_amazonica |
| OTU479 | k__Fungi;p__Ascomycota;c__Sordariomycetes;o__Hypocreales;f__Nectriaceae;g__Dactylonectria;s__Dactylonectria_anthuriicola |
| OTU28 | k__Fungi;p__Ascomycota;c__Sordariomycetes;o__Hypocreales;f__Nectriaceae;g__Fusarium;s__Fusarium_acutatum |
| OTU518 | k__Fungi;p__Ascomycota;c__Sordariomycetes;o__Hypocreales;f__Nectriaceae;g__Fusarium;s__Fusarium_nematophilum |
| OTU471 | k__Fungi;p__Ascomycota;c__Sordariomycetes;o__Hypocreales;f__Nectriaceae;g__Fusarium;s__Fusarium_oxysporum |
| OTU42 | k__Fungi;p__Ascomycota;c__Sordariomycetes;o__Hypocreales;f__Nectriaceae;g__Fusarium;s__Fusarium_solani |
| OTU709 | k__Fungi;p__Ascomycota;c__Sordariomycetes;o__Hypocreales;f__Nectriaceae;g__Fusarium;s__unidentified |
| OTU811 | k__Fungi;p__Ascomycota;c__Sordariomycetes;o__Hypocreales;f__Nectriaceae;g__Gliocephalotrichum;s__Gliocephalotrichum_bulbilium |
| OTU795 | k__Fungi;p__Ascomycota;c__Sordariomycetes;o__Hypocreales;f__Nectriaceae;g__Gliocladiopsis;s__Gliocladiopsis_curvata |
| OTU404 | k__Fungi;p__Ascomycota;c__Sordariomycetes;o__Hypocreales;f__Nectriaceae;g__Gliocladiopsis;s__unidentified |
| OTU592 | k__Fungi;p__Ascomycota;c__Sordariomycetes;o__Hypocreales;f__Nectriaceae;g__Ilyonectria;s__Ilyonectria_lusitanica |
| OTU33 | k__Fungi;p__Ascomycota;c__Sordariomycetes;o__Hypocreales;f__Nectriaceae;g__Ilyonectria;s__Ilyonectria_pseudodestructans |
| OTU891 | k__Fungi;p__Ascomycota;c__Sordariomycetes;o__Hypocreales;f__Nectriaceae;g__Ilyonectria;s__Ilyonectria_radicicola |
| OTU771 | k__Fungi;p__Ascomycota;c__Sordariomycetes;o__Hypocreales;f__Nectriaceae;g__Lasionectria;s__Lasionectria_marigotensis |
| OTU1294 | k__Fungi;p__Ascomycota;c__Sordariomycetes;o__Hypocreales;f__Nectriaceae;g__Microcera;s__Microcera_coccophila |
| OTU629 | k__Fungi;p__Ascomycota;c__Sordariomycetes;o__Hypocreales;f__Nectriaceae;g__Nectria;s__Nectria_balansae |
| OTU408 | k__Fungi;p__Ascomycota;c__Sordariomycetes;o__Hypocreales;f__Nectriaceae;g__Nectria;s__unidentified |
| OTU1047 | k__Fungi;p__Ascomycota;c__Sordariomycetes;o__Hypocreales;f__Nectriaceae;g__Neonectria;s__Neonectria_lugdunensis |
| OTU331 | k__Fungi;p__Ascomycota;c__Sordariomycetes;o__Hypocreales;f__Nectriaceae;g__Paracremonium;s__Paracremonium_binnewijzendii |
| OTU1277 | k__Fungi;p__Ascomycota;c__Sordariomycetes;o__Hypocreales;f__Nectriaceae;g__Paracremonium;s__Paracremonium_inflatum |
| OTU333 | k__Fungi;p__Ascomycota;c__Sordariomycetes;o__Hypocreales;f__Nectriaceae;g__Paracremonium;s__unidentified |
| OTU696 | k__Fungi;p__Ascomycota;c__Sordariomycetes;o__Hypocreales;f__Nectriaceae;g__Penicillifer;s__Penicillifer_diparietisporus |
| OTU1291 | k__Fungi;p__Ascomycota;c__Sordariomycetes;o__Hypocreales;f__Nectriaceae;g__Sarcopodium;s__Sarcopodium_circinosetiferum |
| OTU148 | k__Fungi;p__Ascomycota;c__Sordariomycetes;o__Hypocreales;f__Nectriaceae;g__Stephanonectria;s__Stephanonectria_keithii |
| OTU1109 | k__Fungi;p__Ascomycota;c__Sordariomycetes;o__Hypocreales;f__Nectriaceae;g__Stephanonectria;s__Stephanonectria_keithii |
| OTU1312 | k__Fungi;p__Ascomycota;c__Sordariomycetes;o__Hypocreales;f__Nectriaceae;g__Thelonectria;s__Thelonectria |
| OTU1203 | k__Fungi;p__Ascomycota;c__Sordariomycetes;o__Hypocreales;f__Nectriaceae;g__Thelonectria;s__Thelonectria_coronata |
| OTU1135 | k__Fungi;p__Ascomycota;c__Sordariomycetes;o__Hypocreales;f__Nectriaceae;g__Thelonectria;s__Thelonectria_diademata |
| OTU1311 | k__Fungi;p__Ascomycota;c__Sordariomycetes;o__Hypocreales;f__Nectriaceae;g__Thelonectria;s__Thelonectria_discophora |
| OTU542 | k__Fungi;p__Ascomycota;c__Sordariomycetes;o__Hypocreales;f__Nectriaceae;g__Thelonectria;s__Thelonectria_lucida |
| OTU456 | k__Fungi;p__Ascomycota;c__Sordariomycetes;o__Hypocreales;f__Nectriaceae;g__Thelonectria;s__Thelonectria_mammoidea |
| OTU563 | k__Fungi;p__Ascomycota;c__Sordariomycetes;o__Hypocreales;f__Nectriaceae;g__Thelonectria;s__Thelonectria_stemmata |
| OTU115 | k__Fungi;p__Ascomycota;c__Sordariomycetes;o__Hypocreales;f__Nectriaceae;g__unidentified;s__unidentified |
| OTU298 | k__Fungi;p__Ascomycota;c__Sordariomycetes;o__Hypocreales;f__Nectriaceae;g__unidentified;s__unidentified |
| OTU92 | k__Fungi;p__Ascomycota;c__Sordariomycetes;o__Hypocreales;f__Nectriaceae;g__Volutella;s__unidentified |
| OTU731 | k__Fungi;p__Ascomycota;c__Sordariomycetes;o__Hypocreales;f__Nectriaceae;g__Volutella;s__Volutella_consors |
| OTU448 | k__Fungi;p__Ascomycota;c__Sordariomycetes;o__Hypocreales;f__Niessliaceae;g__Eucasphaeria;s__unidentified |
| OTU361 | k__Fungi;p__Ascomycota;c__Sordariomycetes;o__Hypocreales;f__Ophiocordycipitaceae;g__Hirsutella;s__Hirsutella_guyana |
| OTU943 | k__Fungi;p__Ascomycota;c__Sordariomycetes;o__Hypocreales;f__Ophiocordycipitaceae;g__Hirsutella;s__Hirsutella_liberiana |
| OTU1330 | k__Fungi;p__Ascomycota;c__Sordariomycetes;o__Hypocreales;f__Ophiocordycipitaceae;g__Hirsutella;s__Hirsutella_rostrata |
| OTU874 | k__Fungi;p__Ascomycota;c__Sordariomycetes;o__Hypocreales;f__Ophiocordycipitaceae;g__Ophiocordyceps;s__Ophiocordyceps_arborescens |
| OTU776 | k__Fungi;p__Ascomycota;c__Sordariomycetes;o__Hypocreales;f__Ophiocordycipitaceae;g__Polycephalomyces;s__Polycephalomyces_formosus |
| OTU1174 | k__Fungi;p__Ascomycota;c__Sordariomycetes;o__Hypocreales;f__Ophiocordycipitaceae;g__Polycephalomyces;s__Polycephalomyces_nipponicus |
| OTU798 | k__Fungi;p__Ascomycota;c__Sordariomycetes;o__Hypocreales;f__Ophiocordycipitaceae;g__Purpureocillium;s__unidentified |
| OTU716 | k__Fungi;p__Ascomycota;c__Sordariomycetes;o__Hypocreales;f__Ophiocordycipitaceae;g__Tolypocladium;s__Tolypocladium_album |
| OTU1195 | k__Fungi;p__Ascomycota;c__Sordariomycetes;o__Hypocreales;f__Ophiocordycipitaceae;g__Tolypocladium;s__Tolypocladium_capitatum |
| OTU711 | k__Fungi;p__Ascomycota;c__Sordariomycetes;o__Hypocreales;f__Ophiocordycipitaceae;g__Tolypocladium;s__Tolypocladium_inegoense |
| OTU1093 | k__Fungi;p__Ascomycota;c__Sordariomycetes;o__Hypocreales;f__Ophiocordycipitaceae;g__Tolypocladium;s__Tolypocladium_pustulatum |
| OTU1337 | k__Fungi;p__Ascomycota;c__Sordariomycetes;o__Hypocreales;f__Ophiocordycipitaceae;g__Tolypocladium;s__Tolypocladium_tropicale |
| OTU960 | k__Fungi;p__Ascomycota;c__Sordariomycetes;o__Hypocreales;f__Ophiocordycipitaceae;g__Tolypocladium;s__unidentified |
| OTU501 | k__Fungi;p__Ascomycota;c__Sordariomycetes;o__Hypocreales;f__Ophiocordycipitaceae;g__unidentified;s__unidentified |
| OTU102 | k__Fungi;p__Ascomycota;c__Sordariomycetes;o__Hypocreales;f__Stachybotryaceae;g__Achroiostachys;s__Achroiostachys_humicola |
| OTU1155 | k__Fungi;p__Ascomycota;c__Sordariomycetes;o__Hypocreales;f__Stachybotryaceae;g__Alfaria;s__Alfaria_acaciae |
| OTU1137 | k__Fungi;p__Ascomycota;c__Sordariomycetes;o__Hypocreales;f__Stachybotryaceae;g__Alfaria;s__Alfaria_dandenongensis |
| OTU384 | k__Fungi;p__Ascomycota;c__Sordariomycetes;o__Hypocreales;f__Stachybotryaceae;g__Memnoniella;s__Memnoniella_longistipitata |
| OTU605 | k__Fungi;p__Ascomycota;c__Sordariomycetes;o__Hypocreales;f__Stachybotryaceae;g__Myrothecium;s__Myrothecium_cinctum |
| OTU1187 | k__Fungi;p__Ascomycota;c__Sordariomycetes;o__Hypocreales;f__Stachybotryaceae;g__Myrothecium;s__unidentified |
| OTU1122 | k__Fungi;p__Ascomycota;c__Sordariomycetes;o__Hypocreales;f__Stachybotryaceae;g__Myxospora;s__Myxospora_crassiseta |
| OTU381 | k__Fungi;p__Ascomycota;c__Sordariomycetes;o__Hypocreales;f__Stachybotryaceae;g__Myxospora;s__Myxospora_musae |
| OTU962 | k__Fungi;p__Ascomycota;c__Sordariomycetes;o__Hypocreales;f__Stachybotryaceae;g__Sirastachys;s__Sirastachys_cyperacearum |
| OTU1141 | k__Fungi;p__Ascomycota;c__Sordariomycetes;o__Hypocreales;f__Stachybotryaceae;g__Sirastachys;s__Sirastachys_phyllophila |
| OTU920 | k__Fungi;p__Ascomycota;c__Sordariomycetes;o__Hypocreales;f__Stachybotryaceae;g__Stachybotrys;s__Stachybotrys_aloeticola |
| OTU879 | k__Fungi;p__Ascomycota;c__Sordariomycetes;o__Hypocreales;f__Stachybotryaceae;g__Stachybotrys;s__Stachybotrys_chartarum |
| OTU159 | k__Fungi;p__Ascomycota;c__Sordariomycetes;o__Hypocreales;f__Stachybotryaceae;g__Stachybotrys;s__Stachybotrys_limonispora |
| OTU78 | k__Fungi;p__Ascomycota;c__Sordariomycetes;o__Hypocreales;f__Stachybotryaceae;g__Stachybotrys;s__Stachybotrys_microspora |
| OTU61 | k__Fungi;p__Ascomycota;c__Sordariomycetes;o__Hypocreales;f__Stachybotryaceae;g__Stachybotrys;s__unidentified |
| OTU1243 | k__Fungi;p__Ascomycota;c__Sordariomycetes;o__Hypocreales;f__Stachybotryaceae;g__Striaticonidium;s__Striaticonidium_humicola |
| OTU295 | k__Fungi;p__Ascomycota;c__Sordariomycetes;o__Hypocreales;f__Stachybotryaceae;g__unidentified;s__unidentified |
| OTU598 | k__Fungi;p__Ascomycota;c__Sordariomycetes;o__Hypocreales;f__Stachybotryaceae;g__unidentified;s__unidentified |
| OTU38 | k__Fungi;p__Ascomycota;c__Sordariomycetes;o__Hypocreales;f__unidentified;g__unidentified;s__unidentified |
| OTU67 | k__Fungi;p__Ascomycota;c__Sordariomycetes;o__Hypocreales;f__unidentified;g__unidentified;s__unidentified |
| OTU332 | k__Fungi;p__Ascomycota;c__Sordariomycetes;o__Hypocreales;f__unidentified;g__unidentified;s__unidentified |
| OTU390 | k__Fungi;p__Ascomycota;c__Sordariomycetes;o__Hypocreales;f__unidentified;g__unidentified;s__unidentified |
| OTU724 | k__Fungi;p__Ascomycota;c__Sordariomycetes;o__Magnaporthales;f__Magnaporthaceae;g__Gaeumannomyces;s__Gaeumannomyces_hyphopodioides |
| OTU697 | k__Fungi;p__Ascomycota;c__Sordariomycetes;o__Magnaporthales;f__Magnaporthaceae;g__Mycoleptodiscus;s__unidentified |
| OTU1014 | k__Fungi;p__Ascomycota;c__Sordariomycetes;o__Magnaporthales;f__Magnaporthaceae;g__Mycoleptodiscus;s__unidentified |
| OTU433 | k__Fungi;p__Ascomycota;c__Sordariomycetes;o__Melanosporales;f__Melanosporaceae;g__Melanospora;s__Melanospora_verrucispora |
| OTU691 | k__Fungi;p__Ascomycota;c__Sordariomycetes;o__Microascales;f__Graphiaceae;g__Graphium;s__Graphium_basitruncatum |
| OTU208 | k__Fungi;p__Ascomycota;c__Sordariomycetes;o__Microascales;f__Microascaceae;g__Cephalotrichum;s__Cephalotrichum_stemonitis |
| OTU252 | k__Fungi;p__Ascomycota;c__Sordariomycetes;o__Microascales;f__Microascaceae;g__Cephalotrichum;s__Cephalotrichum_telluricum |
| OTU975 | k__Fungi;p__Ascomycota;c__Sordariomycetes;o__Microascales;f__Microascaceae;g__Cephalotrichum;s__Cephalotrichum_tenuissimum |
| OTU206 | k__Fungi;p__Ascomycota;c__Sordariomycetes;o__Microascales;f__Microascaceae;g__Fairmania;s__Fairmania_singularis |
| OTU1117 | k__Fungi;p__Ascomycota;c__Sordariomycetes;o__Microascales;f__Microascaceae;g__Gamsia;s__Gamsia_aggregata |
| OTU897 | k__Fungi;p__Ascomycota;c__Sordariomycetes;o__Microascales;f__Microascaceae;g__Microascus;s__Microascus_brevicaulis |
| OTU401 | k__Fungi;p__Ascomycota;c__Sordariomycetes;o__Microascales;f__Microascaceae;g__Microascus;s__Microascus_hollandicus |
| OTU855 | k__Fungi;p__Ascomycota;c__Sordariomycetes;o__Microascales;f__Microascaceae;g__Microascus;s__Microascus_intricatus |
| OTU821 | k__Fungi;p__Ascomycota;c__Sordariomycetes;o__Microascales;f__Microascaceae;g__Microascus;s__Microascus_longirostris |
| OTU876 | k__Fungi;p__Ascomycota;c__Sordariomycetes;o__Microascales;f__Microascaceae;g__Pseudallescheria;s__Pseudallescheria_angusta |
| OTU673 | k__Fungi;p__Ascomycota;c__Sordariomycetes;o__Microascales;f__Microascaceae;g__unidentified;s__unidentified |
| OTU366 | k__Fungi;p__Ascomycota;c__Sordariomycetes;o__Microascales;f__unidentified;g__unidentified;s__unidentified |
| OTU327 | k__Fungi;p__Ascomycota;c__Sordariomycetes;o__Myrmecridiales;f__Myrmecridiaceae;g__Myrmecridium;s__Myrmecridium_pulvericola |
| OTU508 | k__Fungi;p__Ascomycota;c__Sordariomycetes;o__Ophiostomatales;f__Ophiostomataceae;g__Ophiostoma;s__Ophiostoma_ssiori |
| OTU131 | k__Fungi;p__Ascomycota;c__Sordariomycetes;o__Ophiostomatales;f__Ophiostomataceae;g__Ophiostoma;s__unidentified |
| OTU1090 | k__Fungi;p__Ascomycota;c__Sordariomycetes;o__Ophiostomatales;f__Ophiostomataceae;g__Raffaelea;s__Raffaelea_scolytodis |
| OTU266 | k__Fungi;p__Ascomycota;c__Sordariomycetes;o__Ophiostomatales;f__Ophiostomataceae;g__Sporothrix;s__Sporothrix_brunneoviolacea |
| OTU585 | k__Fungi;p__Ascomycota;c__Sordariomycetes;o__Ophiostomatales;f__Ophiostomataceae;g__Sporothrix;s__Sporothrix_narcissi |
| OTU1249 | k__Fungi;p__Ascomycota;c__Sordariomycetes;o__Phomatosporales;f__Phomatosporaceae;g__Phomatospora;s__Phomatospora_striatigera |
| OTU1209 | k__Fungi;p__Ascomycota;c__Sordariomycetes;o__Pisorisporiales;f__Pisorisporiaceae;g__Achroceratosphaeria;s__Achroceratosphaeria_potamia |
| OTU886 | k__Fungi;p__Ascomycota;c__Sordariomycetes;o__Pleurotheciales;f__Pleurotheciaceae;g__Phaeoisaria;s__unidentified |
| OTU1162 | k__Fungi;p__Ascomycota;c__Sordariomycetes;o__Pleurotheciales;f__Pleurotheciaceae;g__Pleurothecium;s__Pleurothecium_recurvatum |
| OTU1102 | k__Fungi;p__Ascomycota;c__Sordariomycetes;o__Pleurotheciales;f__Pleurotheciaceae;g__unidentified;s__unidentified |
| OTU284 | k__Fungi;p__Ascomycota;c__Sordariomycetes;o__Savoryellales;f__Savoryellaceae;g__Ascotaiwania;s__Ascotaiwania_fusiformis |
| OTU589 | k__Fungi;p__Ascomycota;c__Sordariomycetes;o__Savoryellales;f__Savoryellaceae;g__Savoryella;s__Savoryella_aquatica |
| OTU88 | k__Fungi;p__Ascomycota;c__Sordariomycetes;o__Sordariales;f__Cephalothecaceae;g__unidentified;s__unidentified |
| OTU453 | k__Fungi;p__Ascomycota;c__Sordariomycetes;o__Sordariales;f__Chaetomiaceae;g__Arcopilus;s__Arcopilus_cupreus |
| OTU1159 | k__Fungi;p__Ascomycota;c__Sordariomycetes;o__Sordariales;f__Chaetomiaceae;g__Arcopilus;s__Arcopilus_fusiformis |
| OTU519 | k__Fungi;p__Ascomycota;c__Sordariomycetes;o__Sordariales;f__Chaetomiaceae;g__Botryotrichum;s__Botryotrichum_atrogriseum |
| OTU297 | k__Fungi;p__Ascomycota;c__Sordariomycetes;o__Sordariales;f__Chaetomiaceae;g__Chaetomium;s__Chaetomium_homopilatum |
| OTU911 | k__Fungi;p__Ascomycota;c__Sordariomycetes;o__Sordariales;f__Chaetomiaceae;g__Chaetomium;s__Chaetomium_subspirilliferum |
| OTU820 | k__Fungi;p__Ascomycota;c__Sordariomycetes;o__Sordariales;f__Chaetomiaceae;g__Chrysanthotrichum;s__Chrysanthotrichum_peruvianum |
| OTU878 | k__Fungi;p__Ascomycota;c__Sordariomycetes;o__Sordariales;f__Chaetomiaceae;g__Dichotomopilus;s__Dichotomopilus_subfunicola |
| OTU308 | k__Fungi;p__Ascomycota;c__Sordariomycetes;o__Sordariales;f__Chaetomiaceae;g__Humicola;s__Humicola_fuscoatra |
| OTU590 | k__Fungi;p__Ascomycota;c__Sordariomycetes;o__Sordariales;f__Chaetomiaceae;g__Humicola;s__Humicola_nigrescens |
| OTU48 | k__Fungi;p__Ascomycota;c__Sordariomycetes;o__Sordariales;f__Chaetomiaceae;g__Humicola;s__Humicola_olivacea |
| OTU157 | k__Fungi;p__Ascomycota;c__Sordariomycetes;o__Sordariales;f__Chaetomiaceae;g__Humicola;s__Humicola_olivacea |
| OTU914 | k__Fungi;p__Ascomycota;c__Sordariomycetes;o__Sordariales;f__Chaetomiaceae;g__Humicola;s__Humicola_phialophoroides |
| OTU742 | k__Fungi;p__Ascomycota;c__Sordariomycetes;o__Sordariales;f__Chaetomiaceae;g__Humicola;s__unidentified |
| OTU1005 | k__Fungi;p__Ascomycota;c__Sordariomycetes;o__Sordariales;f__Chaetomiaceae;g__Melanocarpus;s__Melanocarpus_albomyces |
| OTU862 | k__Fungi;p__Ascomycota;c__Sordariomycetes;o__Sordariales;f__Chaetomiaceae;g__Myceliophthora;s__Myceliophthora_similis |
| OTU1097 | k__Fungi;p__Ascomycota;c__Sordariomycetes;o__Sordariales;f__Chaetomiaceae;g__Mycothermus;s__Mycothermus_thermophilus |
| OTU515 | k__Fungi;p__Ascomycota;c__Sordariomycetes;o__Sordariales;f__Chaetomiaceae;g__Ovatospora;s__Ovatospora_pseudomollicella |
| OTU1111 | k__Fungi;p__Ascomycota;c__Sordariomycetes;o__Sordariales;f__Chaetomiaceae;g__Trichocladium;s__Trichocladium_pyriforme |
| OTU513 | k__Fungi;p__Ascomycota;c__Sordariomycetes;o__Sordariales;f__Chaetomiaceae;g__unidentified;s__unidentified |
| OTU749 | k__Fungi;p__Ascomycota;c__Sordariomycetes;o__Sordariales;f__Chaetomiaceae;g__unidentified;s__unidentified |
| OTU201 | k__Fungi;p__Ascomycota;c__Sordariomycetes;o__Sordariales;f__Chaetomiaceae;g__Zopfiella;s__Zopfiella_longicaudata |
| OTU1299 | k__Fungi;p__Ascomycota;c__Sordariomycetes;o__Sordariales;f__Chaetomiaceae;g__Zopfiella;s__Zopfiella_pleuropora |
| OTU440 | k__Fungi;p__Ascomycota;c__Sordariomycetes;o__Sordariales;f__Chaetomiaceae;g__Zopfiella;s__Zopfiella_tardifaciens |
| OTU686 | k__Fungi;p__Ascomycota;c__Sordariomycetes;o__Sordariales;f__Jobellisiaceae;g__Jobellisia;s__Jobellisia_guangdongensis |
| OTU660 | k__Fungi;p__Ascomycota;c__Sordariomycetes;o__Sordariales;f__Lasiosphaeriaceae;g__Apiosordaria;s__Apiosordaria_microcarpa |
| OTU935 | k__Fungi;p__Ascomycota;c__Sordariomycetes;o__Sordariales;f__Lasiosphaeriaceae;g__Cercophora;s__Cercophora_thailandica |
| OTU934 | k__Fungi;p__Ascomycota;c__Sordariomycetes;o__Sordariales;f__Lasiosphaeriaceae;g__Cercophora;s__unidentified |
| OTU73 | k__Fungi;p__Ascomycota;c__Sordariomycetes;o__Sordariales;f__Lasiosphaeriaceae;g__Cladorrhinum;s__unidentified |
| OTU1110 | k__Fungi;p__Ascomycota;c__Sordariomycetes;o__Sordariales;f__Lasiosphaeriaceae;g__Podospora;s__Podospora_petrogale |
| OTU757 | k__Fungi;p__Ascomycota;c__Sordariomycetes;o__Sordariales;f__Lasiosphaeriaceae;g__Podospora;s__Podospora_serotina |
| OTU1175 | k__Fungi;p__Ascomycota;c__Sordariomycetes;o__Sordariales;f__Lasiosphaeriaceae;g__Strattonia;s__Strattonia_minor |
| OTU296 | k__Fungi;p__Ascomycota;c__Sordariomycetes;o__Sordariales;f__Lasiosphaeriaceae;g__unidentified;s__unidentified |
| OTU403 | k__Fungi;p__Ascomycota;c__Sordariomycetes;o__Sordariales;f__Sordariaceae;g__Diplogelasinospora;s__Diplogelasinospora_grovesii |
| OTU356 | k__Fungi;p__Ascomycota;c__Sordariomycetes;o__Sordariales;f__Sordariaceae;g__Diplogelasinospora;s__Diplogelasinospora_inaequalis |
| OTU158 | k__Fungi;p__Ascomycota;c__Sordariomycetes;o__Sordariales;f__Sordariaceae;g__Neurospora;s__Neurospora_terricola |
| OTU1256 | k__Fungi;p__Ascomycota;c__Sordariomycetes;o__Sordariales;f__Sordariales_fam_Incertae_sedis;g__Cordana;s__Cordana_ellipsoidea |
| OTU305 | k__Fungi;p__Ascomycota;c__Sordariomycetes;o__Sordariales;f__Sordariales_fam_Incertae_sedis;g__Cordana;s__Cordana_terrestris |
| OTU11 | k__Fungi;p__Ascomycota;c__Sordariomycetes;o__Sordariales;f__Sordariales_fam_Incertae_sedis;g__Dendrosporium;s__Dendrosporium_lobatum |
| OTU198 | k__Fungi;p__Ascomycota;c__Sordariomycetes;o__Sordariales;f__Sordariales_fam_Incertae_sedis;g__Papulaspora;s__Papulaspora_funabasensis |
| OTU985 | k__Fungi;p__Ascomycota;c__Sordariomycetes;o__Sordariales;f__Sordariales_fam_Incertae_sedis;g__Ramophialophora;s__unidentified |
| OTU1338 | k__Fungi;p__Ascomycota;c__Sordariomycetes;o__Sordariales;f__Sordariales_fam_Incertae_sedis;g__Remersonia;s__Remersonia_thermophila |
| OTU432 | k__Fungi;p__Ascomycota;c__Sordariomycetes;o__Sordariales;f__Sordariales_fam_Incertae_sedis;g__Staphylotrichum;s__Staphylotrichum_boninense |
| OTU554 | k__Fungi;p__Ascomycota;c__Sordariomycetes;o__Sordariales;f__unidentified;g__unidentified;s__unidentified |
| OTU849 | k__Fungi;p__Ascomycota;c__Sordariomycetes;o__Sordariomycetes_ord_Incertae_sedis;f__Sordariomycetes_fam_Incertae_sedis;g__Distoseptispora;s__Distoseptispora_fluminicola |
| OTU355 | k__Fungi;p__Ascomycota;c__Sordariomycetes;o__Sordariomycetes_ord_Incertae_sedis;f__Sordariomycetes_fam_Incertae_sedis;g__Pleurophragmium;s__unidentified |
| OTU511 | k__Fungi;p__Ascomycota;c__Sordariomycetes;o__Sordariomycetes_ord_Incertae_sedis;f__Sordariomycetes_fam_Incertae_sedis;g__Xylomelasma;s__unidentified |
| OTU745 | k__Fungi;p__Ascomycota;c__Sordariomycetes;o__Sordariomycetidae_ord_Incertae_sedis;f__Rhamphoriaceae;g__Xylolentia;s__Xylolentia_brunneola |
| OTU1248 | k__Fungi;p__Ascomycota;c__Sordariomycetes;o__Togniniales;f__Togniniaceae;g__Phaeoacremonium;s__Phaeoacremonium_amstelodamense |
| OTU22 | k__Fungi;p__Ascomycota;c__Sordariomycetes;o__Togniniales;f__Togniniaceae;g__Phaeoacremonium;s__Phaeoacremonium_canadense |
| OTU718 | k__Fungi;p__Ascomycota;c__Sordariomycetes;o__Togniniales;f__Togniniaceae;g__Phaeoacremonium;s__Phaeoacremonium_fuscum |
| OTU631 | k__Fungi;p__Ascomycota;c__Sordariomycetes;o__Togniniales;f__Togniniaceae;g__Phaeoacremonium;s__Phaeoacremonium_novae-zelandiae |
| OTU123 | k__Fungi;p__Ascomycota;c__Sordariomycetes;o__unidentified;f__unidentified;g__unidentified;s__unidentified |
| OTU130 | k__Fungi;p__Ascomycota;c__Sordariomycetes;o__unidentified;f__unidentified;g__unidentified;s__unidentified |
| OTU191 | k__Fungi;p__Ascomycota;c__Sordariomycetes;o__unidentified;f__unidentified;g__unidentified;s__unidentified |
| OTU204 | k__Fungi;p__Ascomycota;c__Sordariomycetes;o__unidentified;f__unidentified;g__unidentified;s__unidentified |
| OTU328 | k__Fungi;p__Ascomycota;c__Sordariomycetes;o__unidentified;f__unidentified;g__unidentified;s__unidentified |
| OTU579 | k__Fungi;p__Ascomycota;c__Sordariomycetes;o__unidentified;f__unidentified;g__unidentified;s__unidentified |
| OTU919 | k__Fungi;p__Ascomycota;c__Sordariomycetes;o__unidentified;f__unidentified;g__unidentified;s__unidentified |
| OTU706 | k__Fungi;p__Ascomycota;c__Sordariomycetes;o__Xylariales;f__Apiosporaceae;g__Arthrinium;s__Arthrinium_guizhouense |
| OTU1301 | k__Fungi;p__Ascomycota;c__Sordariomycetes;o__Xylariales;f__Apiosporaceae;g__Arthrinium;s__Arthrinium_gutiae |
| OTU1226 | k__Fungi;p__Ascomycota;c__Sordariomycetes;o__Xylariales;f__Apiosporaceae;g__Arthrinium;s__Arthrinium_obovatum |
| OTU516 | k__Fungi;p__Ascomycota;c__Sordariomycetes;o__Xylariales;f__Apiosporaceae;g__Arthrinium;s__Arthrinium_rasikravindrae |
| OTU417 | k__Fungi;p__Ascomycota;c__Sordariomycetes;o__Xylariales;f__Apiosporaceae;g__Arthrinium;s__unidentified |
| OTU822 | k__Fungi;p__Ascomycota;c__Sordariomycetes;o__Xylariales;f__Apiosporaceae;g__unidentified;s__unidentified |
| OTU611 | k__Fungi;p__Ascomycota;c__Sordariomycetes;o__Xylariales;f__Bartaliniaceae;g__Bartalinia;s__Bartalinia_pondoensis |
| OTU984 | k__Fungi;p__Ascomycota;c__Sordariomycetes;o__Xylariales;f__Beltraniaceae;g__Beltraniella;s__Beltraniella_acaciae |
| OTU215 | k__Fungi;p__Ascomycota;c__Sordariomycetes;o__Xylariales;f__Beltraniaceae;g__Beltraniella;s__Beltraniella_endiandrae |
| OTU667 | k__Fungi;p__Ascomycota;c__Sordariomycetes;o__Xylariales;f__Diatrypaceae;g__Diatrypella;s__Diatrypella_heveae |
| OTU1279 | k__Fungi;p__Ascomycota;c__Sordariomycetes;o__Xylariales;f__Diatrypaceae;g__Peroneutypa;s__Peroneutypa_diminutiasca |
| OTU1210 | k__Fungi;p__Ascomycota;c__Sordariomycetes;o__Xylariales;f__Diatrypaceae;g__Peroneutypa;s__Peroneutypa_scoparia |
| OTU238 | k__Fungi;p__Ascomycota;c__Sordariomycetes;o__Xylariales;f__Hyponectriaceae;g__Physalospora;s__Physalospora_vaccinii |
| OTU1252 | k__Fungi;p__Ascomycota;c__Sordariomycetes;o__Xylariales;f__Microdochiaceae;g__Microdochium;s__Microdochium_lycopodinum |
| OTU1080 | k__Fungi;p__Ascomycota;c__Sordariomycetes;o__Xylariales;f__Sporocadaceae;g__Discosia;s__Discosia_pseudoartocreas |
| OTU2 | k__Fungi;p__Ascomycota;c__Sordariomycetes;o__Xylariales;f__Sporocadaceae;g__Discosia;s__unidentified |
| OTU529 | k__Fungi;p__Ascomycota;c__Sordariomycetes;o__Xylariales;f__Sporocadaceae;g__Pestalotiopsis;s__Pestalotiopsis_anacardiacearum |
| OTU1272 | k__Fungi;p__Ascomycota;c__Sordariomycetes;o__Xylariales;f__Sporocadaceae;g__Pestalotiopsis;s__Pestalotiopsis_jesteri |
| OTU340 | k__Fungi;p__Ascomycota;c__Sordariomycetes;o__Xylariales;f__Sporocadaceae;g__Pestalotiopsis;s__Pestalotiopsis_mangifolia |
| OTU743 | k__Fungi;p__Ascomycota;c__Sordariomycetes;o__Xylariales;f__Sporocadaceae;g__Pestalotiopsis;s__Pestalotiopsis_unicolor |
| OTU1216 | k__Fungi;p__Ascomycota;c__Sordariomycetes;o__Xylariales;f__Sporocadaceae;g__Seiridium;s__Seiridium_camelliae |
| OTU977 | k__Fungi;p__Ascomycota;c__Sordariomycetes;o__Xylariales;f__Sporocadaceae;g__Seiridium;s__Seiridium_phylicae |
| OTU1064 | k__Fungi;p__Ascomycota;c__Sordariomycetes;o__Xylariales;f__Sporocadaceae;g__Seiridium;s__unidentified |
| OTU490 | k__Fungi;p__Ascomycota;c__Sordariomycetes;o__Xylariales;f__unidentified;g__unidentified;s__unidentified |
| OTU596 | k__Fungi;p__Ascomycota;c__Sordariomycetes;o__Xylariales;f__Xylariaceae;g__Amphirosellinia;s__Amphirosellinia_fushanensis |
| OTU460 | k__Fungi;p__Ascomycota;c__Sordariomycetes;o__Xylariales;f__Xylariaceae;g__Annulohypoxylon;s__Annulohypoxylon_archeri |
| OTU827 | k__Fungi;p__Ascomycota;c__Sordariomycetes;o__Xylariales;f__Xylariaceae;g__Annulohypoxylon;s__Annulohypoxylon_bovei |
| OTU374 | k__Fungi;p__Ascomycota;c__Sordariomycetes;o__Xylariales;f__Xylariaceae;g__Annulohypoxylon;s__Annulohypoxylon_multiforme |
| OTU1238 | k__Fungi;p__Ascomycota;c__Sordariomycetes;o__Xylariales;f__Xylariaceae;g__Annulohypoxylon;s__Annulohypoxylon_stygium |
| OTU495 | k__Fungi;p__Ascomycota;c__Sordariomycetes;o__Xylariales;f__Xylariaceae;g__Anthostomella;s__unidentified |
| OTU1059 | k__Fungi;p__Ascomycota;c__Sordariomycetes;o__Xylariales;f__Xylariaceae;g__Astrocystis;s__Astrocystis_bambusae |
| OTU1333 | k__Fungi;p__Ascomycota;c__Sordariomycetes;o__Xylariales;f__Xylariaceae;g__Astrocystis;s__Astrocystis_mirabilis |
| OTU1104 | k__Fungi;p__Ascomycota;c__Sordariomycetes;o__Xylariales;f__Xylariaceae;g__Biscogniauxia;s__unidentified |
| OTU571 | k__Fungi;p__Ascomycota;c__Sordariomycetes;o__Xylariales;f__Xylariaceae;g__Daldinia;s__Daldinia_starbaeckii |
| OTU171 | k__Fungi;p__Ascomycota;c__Sordariomycetes;o__Xylariales;f__Xylariaceae;g__Daldinia;s__unidentified |
| OTU197 | k__Fungi;p__Ascomycota;c__Sordariomycetes;o__Xylariales;f__Xylariaceae;g__Emarcea;s__Emarcea_eucalyptigena |
| OTU1181 | k__Fungi;p__Ascomycota;c__Sordariomycetes;o__Xylariales;f__Xylariaceae;g__Geniculisynnema;s__Geniculisynnema_termiticola |
| OTU207 | k__Fungi;p__Ascomycota;c__Sordariomycetes;o__Xylariales;f__Xylariaceae;g__Hypoxylon;s__Hypoxylon_anthochroum |
| OTU1202 | k__Fungi;p__Ascomycota;c__Sordariomycetes;o__Xylariales;f__Xylariaceae;g__Hypoxylon;s__Hypoxylon_begae |
| OTU1038 | k__Fungi;p__Ascomycota;c__Sordariomycetes;o__Xylariales;f__Xylariaceae;g__Hypoxylon;s__Hypoxylon_carneum |
| OTU1158 | k__Fungi;p__Ascomycota;c__Sordariomycetes;o__Xylariales;f__Xylariaceae;g__Hypoxylon;s__Hypoxylon_cercidicola |
| OTU570 | k__Fungi;p__Ascomycota;c__Sordariomycetes;o__Xylariales;f__Xylariaceae;g__Hypoxylon;s__Hypoxylon_commutatum |
| OTU840 | k__Fungi;p__Ascomycota;c__Sordariomycetes;o__Xylariales;f__Xylariaceae;g__Hypoxylon;s__Hypoxylon_diatrypeoides |
| OTU1259 | k__Fungi;p__Ascomycota;c__Sordariomycetes;o__Xylariales;f__Xylariaceae;g__Hypoxylon;s__Hypoxylon_duranii |
| OTU1087 | k__Fungi;p__Ascomycota;c__Sordariomycetes;o__Xylariales;f__Xylariaceae;g__Hypoxylon;s__Hypoxylon_fendleri |
| OTU1287 | k__Fungi;p__Ascomycota;c__Sordariomycetes;o__Xylariales;f__Xylariaceae;g__Hypoxylon;s__Hypoxylon_haematostroma |
| OTU990 | k__Fungi;p__Ascomycota;c__Sordariomycetes;o__Xylariales;f__Xylariaceae;g__Hypoxylon;s__Hypoxylon_jaklitschii |
| OTU551 | k__Fungi;p__Ascomycota;c__Sordariomycetes;o__Xylariales;f__Xylariaceae;g__Hypoxylon;s__Hypoxylon_kanchanapisekii |
| OTU1099 | k__Fungi;p__Ascomycota;c__Sordariomycetes;o__Xylariales;f__Xylariaceae;g__Hypoxylon;s__Hypoxylon_monticulosum |
| OTU251 | k__Fungi;p__Ascomycota;c__Sordariomycetes;o__Xylariales;f__Xylariaceae;g__Hypoxylon;s__Hypoxylon_notatum |
| OTU1224 | k__Fungi;p__Ascomycota;c__Sordariomycetes;o__Xylariales;f__Xylariaceae;g__Hypoxylon;s__Hypoxylon_pseudefendleri |
| OTU481 | k__Fungi;p__Ascomycota;c__Sordariomycetes;o__Xylariales;f__Xylariaceae;g__Hypoxylon;s__Hypoxylon_pulicicidum |
| OTU803 | k__Fungi;p__Ascomycota;c__Sordariomycetes;o__Xylariales;f__Xylariaceae;g__Hypoxylon;s__Hypoxylon_rubiginosum |
| OTU550 | k__Fungi;p__Ascomycota;c__Sordariomycetes;o__Xylariales;f__Xylariaceae;g__Hypoxylon;s__Hypoxylon_sublenormandii |
| OTU3 | k__Fungi;p__Ascomycota;c__Sordariomycetes;o__Xylariales;f__Xylariaceae;g__Hypoxylon;s__unidentified |
| OTU476 | k__Fungi;p__Ascomycota;c__Sordariomycetes;o__Xylariales;f__Xylariaceae;g__Kretzschmaria;s__Kretzschmaria_iranica |
| OTU543 | k__Fungi;p__Ascomycota;c__Sordariomycetes;o__Xylariales;f__Xylariaceae;g__Nemania;s__Nemania_bipapillata |
| OTU933 | k__Fungi;p__Ascomycota;c__Sordariomycetes;o__Xylariales;f__Xylariaceae;g__Nemania;s__Nemania_plumbea |
| OTU497 | k__Fungi;p__Ascomycota;c__Sordariomycetes;o__Xylariales;f__Xylariaceae;g__Nemania;s__unidentified |
| OTU702 | k__Fungi;p__Ascomycota;c__Sordariomycetes;o__Xylariales;f__Xylariaceae;g__Pyrenopolyporus;s__Pyrenopolyporus_laminosus |
| OTU639 | k__Fungi;p__Ascomycota;c__Sordariomycetes;o__Xylariales;f__Xylariaceae;g__Rosellinia;s__Rosellinia_necatrix |
| OTU64 | k__Fungi;p__Ascomycota;c__Sordariomycetes;o__Xylariales;f__Xylariaceae;g__unidentified;s__unidentified |
| OTU209 | k__Fungi;p__Ascomycota;c__Sordariomycetes;o__Xylariales;f__Xylariaceae;g__unidentified;s__unidentified |
| OTU271 | k__Fungi;p__Ascomycota;c__Sordariomycetes;o__Xylariales;f__Xylariaceae;g__unidentified;s__unidentified |
| OTU504 | k__Fungi;p__Ascomycota;c__Sordariomycetes;o__Xylariales;f__Xylariaceae;g__unidentified;s__unidentified |
| OTU535 | k__Fungi;p__Ascomycota;c__Sordariomycetes;o__Xylariales;f__Xylariaceae;g__unidentified;s__unidentified |
| OTU1036 | k__Fungi;p__Ascomycota;c__Sordariomycetes;o__Xylariales;f__Xylariaceae;g__unidentified;s__unidentified |
| OTU1086 | k__Fungi;p__Ascomycota;c__Sordariomycetes;o__Xylariales;f__Xylariaceae;g__unidentified;s__unidentified |
| OTU832 | k__Fungi;p__Ascomycota;c__Sordariomycetes;o__Xylariales;f__Xylariaceae;g__Virgaria;s__Virgaria_nigra |
| OTU469 | k__Fungi;p__Ascomycota;c__Sordariomycetes;o__Xylariales;f__Xylariaceae;g__Xylaria;s__unidentified |
| OTU599 | k__Fungi;p__Ascomycota;c__Sordariomycetes;o__Xylariales;f__Xylariaceae;g__Xylaria;s__Xylaria_badia |
| OTU1149 | k__Fungi;p__Ascomycota;c__Sordariomycetes;o__Xylariales;f__Xylariaceae;g__Xylaria;s__Xylaria_bambusicola |
| OTU937 | k__Fungi;p__Ascomycota;c__Sordariomycetes;o__Xylariales;f__Xylariaceae;g__Xylaria;s__Xylaria_brevipes |
| OTU1281 | k__Fungi;p__Ascomycota;c__Sordariomycetes;o__Xylariales;f__Xylariaceae;g__Xylaria;s__Xylaria_coccophora |
| OTU1144 | k__Fungi;p__Ascomycota;c__Sordariomycetes;o__Xylariales;f__Xylariaceae;g__Xylaria;s__Xylaria_hongkongensis |
| OTU817 | k__Fungi;p__Ascomycota;c__Sordariomycetes;o__Xylariales;f__Xylariaceae;g__Xylaria;s__Xylaria_hypoxylon |
| OTU947 | k__Fungi;p__Ascomycota;c__Sordariomycetes;o__Xylariales;f__Xylariaceae;g__Xylaria;s__Xylaria_intracolorata |
| OTU1239 | k__Fungi;p__Ascomycota;c__Sordariomycetes;o__Xylariales;f__Xylariaceae;g__Xylaria;s__Xylaria_ochraceostroma |
| OTU1283 | k__Fungi;p__Ascomycota;c__Sordariomycetes;o__Xylariales;f__Xylariaceae;g__Zygosporium;s__unidentified |
| OTU165 | k__Fungi;p__Ascomycota;c__Sordariomycetes;o__Xylariales;f__Xylariales_fam_Incertae_sedis;g__Castanediella;s__Castanediella_couratarii |
| OTU104 | k__Fungi;p__Ascomycota;c__Sordariomycetes;o__Xylariales;f__Xylariales_fam_Incertae_sedis;g__Castanediella;s__Castanediella_eucalypti |
| OTU380 | k__Fungi;p__Ascomycota;c__Sordariomycetes;o__Xylariales;f__Xylariales_fam_Incertae_sedis;g__Castanediella;s__unidentified |
| OTU431 | k__Fungi;p__Ascomycota;c__Sordariomycetes;o__Xylariales;f__Xylariales_fam_Incertae_sedis;g__Liberomyces;s__unidentified |
| OTU454 | k__Fungi;p__Ascomycota;c__Sordariomycetes;o__Xylariales;f__Xylariales_fam_Incertae_sedis;g__Phialemoniopsis;s__Phialemoniopsis_cornearis |
| OTU473 | k__Fungi;p__Ascomycota;c__Sordariomycetes;o__Xylariales;f__Xylariales_fam_Incertae_sedis;g__Robillarda;s__Robillarda_sessilis |
| OTU4 | k__Fungi;p__Ascomycota;c__unidentified;o__unidentified;f__unidentified;g__unidentified;s__unidentified |

**Basidiomycita**

| **OUT number** | **Taxonomy** |
| --- | --- |
| OTU950 | k__Fungi;p__Basidiomycota;c__Agaricomycetes;o__Agaricales;f__Agaricaceae;g__Agaricus;s__Agaricus_atrodiscus |
| OTU143 | k__Fungi;p__Basidiomycota;c__Agaricomycetes;o__Agaricales;f__Agaricaceae;g__Agaricus;s__Agaricus_brasiliensis |
| OTU1050 | k__Fungi;p__Basidiomycota;c__Agaricomycetes;o__Agaricales;f__Agaricaceae;g__Agaricus;s__Agaricus_brunneogracilis |
| OTU1105 | k__Fungi;p__Basidiomycota;c__Agaricomycetes;o__Agaricales;f__Agaricaceae;g__Agaricus;s__Agaricus_flocculosipes |
| OTU1280 | k__Fungi;p__Basidiomycota;c__Agaricomycetes;o__Agaricales;f__Agaricaceae;g__Agaricus;s__Agaricus_microvolvatulus |
| OTU250 | k__Fungi;p__Basidiomycota;c__Agaricomycetes;o__Agaricales;f__Agaricaceae;g__Agaricus;s__Agaricus_subrutilescens |
| OTU559 | k__Fungi;p__Basidiomycota;c__Agaricomycetes;o__Agaricales;f__Agaricaceae;g__Agaricus;s__Agaricus_variicystis |
| OTU225 | k__Fungi;p__Basidiomycota;c__Agaricomycetes;o__Agaricales;f__Agaricaceae;g__Agaricus;s__Agaricus_xanthosarcus |
| OTU725 | k__Fungi;p__Basidiomycota;c__Agaricomycetes;o__Agaricales;f__Agaricaceae;g__Agaricus;s__unidentified |
| OTU397 | k__Fungi;p__Basidiomycota;c__Agaricomycetes;o__Agaricales;f__Agaricaceae;g__Chlorophyllum;s__Chlorophyllum_hortense |
| OTU761 | k__Fungi;p__Basidiomycota;c__Agaricomycetes;o__Agaricales;f__Agaricaceae;g__Lepiota;s__Lepiota_himalayensis |
| OTU1133 | k__Fungi;p__Basidiomycota;c__Agaricomycetes;o__Agaricales;f__Agaricaceae;g__Lepiota;s__Lepiota_psalion |
| OTU95 | k__Fungi;p__Basidiomycota;c__Agaricomycetes;o__Agaricales;f__Agaricaceae;g__Lepiota;s__Lepiota_thrombophora |
| OTU521 | k__Fungi;p__Basidiomycota;c__Agaricomycetes;o__Agaricales;f__Agaricaceae;g__Lepiota;s__unidentified |
| OTU871 | k__Fungi;p__Basidiomycota;c__Agaricomycetes;o__Agaricales;f__Agaricaceae;g__Leucoagaricus;s__Leucoagaricus_flavovirens |
| OTU751 | k__Fungi;p__Basidiomycota;c__Agaricomycetes;o__Agaricales;f__Agaricaceae;g__Leucoagaricus;s__Leucoagaricus_rubrotinctus |
| OTU587 | k__Fungi;p__Basidiomycota;c__Agaricomycetes;o__Agaricales;f__Agaricaceae;g__Leucoagaricus;s__unidentified |
| OTU463 | k__Fungi;p__Basidiomycota;c__Agaricomycetes;o__Agaricales;f__Agaricaceae;g__Macrolepiota;s__Macrolepiota_velosa |
| OTU737 | k__Fungi;p__Basidiomycota;c__Agaricomycetes;o__Agaricales;f__Agaricaceae;g__Melanophyllum;s__Melanophyllum_haematospermum |
| OTU69 | k__Fungi;p__Basidiomycota;c__Agaricomycetes;o__Agaricales;f__Agaricaceae;g__Micropsalliota;s__Micropsalliota_globocystis |
| OTU267 | k__Fungi;p__Basidiomycota;c__Agaricomycetes;o__Agaricales;f__Agaricaceae;g__Micropsalliota;s__Micropsalliota_megaspora |
| OTU1136 | k__Fungi;p__Basidiomycota;c__Agaricomycetes;o__Agaricales;f__Agaricaceae;g__Micropsalliota;s__Micropsalliota_pusillissima |
| OTU43 | k__Fungi;p__Basidiomycota;c__Agaricomycetes;o__Agaricales;f__Agaricaceae;g__Micropsalliota;s__unidentified |
| OTU211 | k__Fungi;p__Basidiomycota;c__Agaricomycetes;o__Agaricales;f__Agaricaceae;g__Tulostoma;s__unidentified |
| OTU314 | k__Fungi;p__Basidiomycota;c__Agaricomycetes;o__Agaricales;f__Agaricaceae;g__unidentified;s__unidentified |
| OTU353 | k__Fungi;p__Basidiomycota;c__Agaricomycetes;o__Agaricales;f__Amanitaceae;g__Amanita;s__Amanita_esculenta |
| OTU904 | k__Fungi;p__Basidiomycota;c__Agaricomycetes;o__Agaricales;f__Amanitaceae;g__Amanita;s__Amanita_fritillaria |
| OTU675 | k__Fungi;p__Basidiomycota;c__Agaricomycetes;o__Agaricales;f__Amanitaceae;g__Amanita;s__Amanita_glarea |
| OTU70 | k__Fungi;p__Basidiomycota;c__Agaricomycetes;o__Agaricales;f__Amanitaceae;g__Amanita;s__Amanita_hemibapha |
| OTU520 | k__Fungi;p__Basidiomycota;c__Agaricomycetes;o__Agaricales;f__Amanitaceae;g__Amanita;s__Amanita_japonica |
| OTU1058 | k__Fungi;p__Basidiomycota;c__Agaricomycetes;o__Agaricales;f__Amanitaceae;g__Amanita;s__Amanita_olivaceofusca |
| OTU1067 | k__Fungi;p__Basidiomycota;c__Agaricomycetes;o__Agaricales;f__Amanitaceae;g__Amanita;s__Amanita_parvicurta |
| OTU845 | k__Fungi;p__Basidiomycota;c__Agaricomycetes;o__Agaricales;f__Amanitaceae;g__Amanita;s__Amanita_parvipantherina |
| OTU385 | k__Fungi;p__Basidiomycota;c__Agaricomycetes;o__Agaricales;f__Amanitaceae;g__Amanita;s__Amanita_rubiginosa |
| OTU800 | k__Fungi;p__Basidiomycota;c__Agaricomycetes;o__Agaricales;f__Amanitaceae;g__Amanita;s__Amanita_rubrovolvata |
| OTU170 | k__Fungi;p__Basidiomycota;c__Agaricomycetes;o__Agaricales;f__Amanitaceae;g__Amanita;s__Amanita_velosa |
| OTU220 | k__Fungi;p__Basidiomycota;c__Agaricomycetes;o__Agaricales;f__Amanitaceae;g__Amanita;s__unidentified |
| OTU427 | k__Fungi;p__Basidiomycota;c__Agaricomycetes;o__Agaricales;f__Bolbitiaceae;g__Conocybe;s__unidentified |
| OTU154 | k__Fungi;p__Basidiomycota;c__Agaricomycetes;o__Agaricales;f__Bolbitiaceae;g__unidentified;s__unidentified |
| OTU1308 | k__Fungi;p__Basidiomycota;c__Agaricomycetes;o__Agaricales;f__Catathelasmataceae;g__Pseudolaccaria;s__Pseudolaccaria_pachyphylla |
| OTU616 | k__Fungi;p__Basidiomycota;c__Agaricomycetes;o__Agaricales;f__Clavariaceae;g__Clavaria;s__Clavaria_acuta |
| OTU472 | k__Fungi;p__Basidiomycota;c__Agaricomycetes;o__Agaricales;f__Clavariaceae;g__Clavaria;s__Clavaria_falcata |
| OTU1153 | k__Fungi;p__Basidiomycota;c__Agaricomycetes;o__Agaricales;f__Clavariaceae;g__Clavaria;s__Clavaria_pullei |
| OTU772 | k__Fungi;p__Basidiomycota;c__Agaricomycetes;o__Agaricales;f__Clavariaceae;g__Clavaria;s__Clavaria_zollingeri |
| OTU929 | k__Fungi;p__Basidiomycota;c__Agaricomycetes;o__Agaricales;f__Clavariaceae;g__Clavaria;s__unidentified |
| OTU748 | k__Fungi;p__Basidiomycota;c__Agaricomycetes;o__Agaricales;f__Clavariaceae;g__Clavulinopsis;s__Clavulinopsis_luteonana |
| OTU1033 | k__Fungi;p__Basidiomycota;c__Agaricomycetes;o__Agaricales;f__Clavariaceae;g__Hodophilus;s__unidentified |
| OTU1284 | k__Fungi;p__Basidiomycota;c__Agaricomycetes;o__Agaricales;f__Clavariaceae;g__Mucronella;s__unidentified |
| OTU952 | k__Fungi;p__Basidiomycota;c__Agaricomycetes;o__Agaricales;f__Clavariaceae;g__unidentified;s__unidentified |
| OTU1217 | k__Fungi;p__Basidiomycota;c__Agaricomycetes;o__Agaricales;f__Cortinariaceae;g__Cortinarius;s__Cortinarius_barbaricus |
| OTU1204 | k__Fungi;p__Basidiomycota;c__Agaricomycetes;o__Agaricales;f__Cortinariaceae;g__Cortinarius;s__Cortinarius_biriensis |
| OTU325 | k__Fungi;p__Basidiomycota;c__Agaricomycetes;o__Agaricales;f__Cortinariaceae;g__Cortinarius;s__Cortinarius_saturatus |
| OTU444 | k__Fungi;p__Basidiomycota;c__Agaricomycetes;o__Agaricales;f__Cortinariaceae;g__Cortinarius;s__unidentified |
| OTU1197 | k__Fungi;p__Basidiomycota;c__Agaricomycetes;o__Agaricales;f__Cortinariaceae;g__Gymnopilus;s__Gymnopilus_decipiens |
| OTU1225 | k__Fungi;p__Basidiomycota;c__Agaricomycetes;o__Agaricales;f__Cortinariaceae;g__Gymnopilus;s__Gymnopilus_liquiritiae |
| OTU1032 | k__Fungi;p__Basidiomycota;c__Agaricomycetes;o__Agaricales;f__Cortinariaceae;g__Gymnopilus;s__Gymnopilus_odini |
| OTU1268 | k__Fungi;p__Basidiomycota;c__Agaricomycetes;o__Agaricales;f__Cortinariaceae;g__Gymnopilus;s__Gymnopilus_penetrans |
| OTU1154 | k__Fungi;p__Basidiomycota;c__Agaricomycetes;o__Agaricales;f__Cortinariaceae;g__Gymnopilus;s__Gymnopilus_tyallus |
| OTU478 | k__Fungi;p__Basidiomycota;c__Agaricomycetes;o__Agaricales;f__Crepidotaceae;g__Crepidotus;s__unidentified |
| OTU964 | k__Fungi;p__Basidiomycota;c__Agaricomycetes;o__Agaricales;f__Crepidotaceae;g__Simocybe;s__unidentified |
| OTU948 | k__Fungi;p__Basidiomycota;c__Agaricomycetes;o__Agaricales;f__Cyphellaceae;g__unidentified;s__unidentified |
| OTU780 | k__Fungi;p__Basidiomycota;c__Agaricomycetes;o__Agaricales;f__Entolomataceae;g__Clitopilus;s__Clitopilus_hobsonii |
| OTU976 | k__Fungi;p__Basidiomycota;c__Agaricomycetes;o__Agaricales;f__Entolomataceae;g__Clitopilus;s__Clitopilus_prunulus |
| OTU1205 | k__Fungi;p__Basidiomycota;c__Agaricomycetes;o__Agaricales;f__Entolomataceae;g__Clitopilus;s__unidentified |
| OTU858 | k__Fungi;p__Basidiomycota;c__Agaricomycetes;o__Agaricales;f__Entolomataceae;g__Entoloma;s__Entoloma_bloxamii |
| OTU135 | k__Fungi;p__Basidiomycota;c__Agaricomycetes;o__Agaricales;f__Entolomataceae;g__Entoloma;s__Entoloma_brunneoumbonatum |
| OTU868 | k__Fungi;p__Basidiomycota;c__Agaricomycetes;o__Agaricales;f__Entolomataceae;g__Entoloma;s__Entoloma_conferendum |
| OTU609 | k__Fungi;p__Basidiomycota;c__Agaricomycetes;o__Agaricales;f__Entolomataceae;g__Entoloma;s__Entoloma_llimonae |
| OTU434 | k__Fungi;p__Basidiomycota;c__Agaricomycetes;o__Agaricales;f__Entolomataceae;g__Entoloma;s__Entoloma_percoelestinum |
| OTU604 | k__Fungi;p__Basidiomycota;c__Agaricomycetes;o__Agaricales;f__Entolomataceae;g__Entoloma;s__Entoloma_rhodopolium |
| OTU36 | k__Fungi;p__Basidiomycota;c__Agaricomycetes;o__Agaricales;f__Entolomataceae;g__Entoloma;s__Entoloma_rivulare |
| OTU468 | k__Fungi;p__Basidiomycota;c__Agaricomycetes;o__Agaricales;f__Entolomataceae;g__Entoloma;s__unidentified |
| OTU1037 | k__Fungi;p__Basidiomycota;c__Agaricomycetes;o__Agaricales;f__Entolomataceae;g__Rhodocybe;s__Rhodocybe_asyae |
| OTU82 | k__Fungi;p__Basidiomycota;c__Agaricomycetes;o__Agaricales;f__Entolomataceae;g__unidentified;s__unidentified |
| OTU1017 | k__Fungi;p__Basidiomycota;c__Agaricomycetes;o__Agaricales;f__Fistulinaceae;g__Porodisculus;s__unidentified |
| OTU738 | k__Fungi;p__Basidiomycota;c__Agaricomycetes;o__Agaricales;f__Hydnangiaceae;g__Laccaria;s__Laccaria_fulvogrisea |
| OTU661 | k__Fungi;p__Basidiomycota;c__Agaricomycetes;o__Agaricales;f__Hydnangiaceae;g__Laccaria;s__Laccaria_japonica |
| OTU1081 | k__Fungi;p__Basidiomycota;c__Agaricomycetes;o__Agaricales;f__Hydnangiaceae;g__Laccaria;s__Laccaria_vinaceoavellanea |
| OTU988 | k__Fungi;p__Basidiomycota;c__Agaricomycetes;o__Agaricales;f__Hydnangiaceae;g__Laccaria;s__Laccaria_yunnanensis |
| OTU750 | k__Fungi;p__Basidiomycota;c__Agaricomycetes;o__Agaricales;f__Hydnangiaceae;g__Laccaria;s__unidentified |
| OTU1092 | k__Fungi;p__Basidiomycota;c__Agaricomycetes;o__Agaricales;f__Hygrophoraceae;g__Hygrocybe;s__Hygrocybe_coccinea |
| OTU593 | k__Fungi;p__Basidiomycota;c__Agaricomycetes;o__Agaricales;f__Hygrophoraceae;g__Hygrocybe;s__Hygrocybe_intermedia |
| OTU1303 | k__Fungi;p__Basidiomycota;c__Agaricomycetes;o__Agaricales;f__Hygrophoraceae;g__Hygrocybe;s__Hygrocybe_miniata |
| OTU149 | k__Fungi;p__Basidiomycota;c__Agaricomycetes;o__Agaricales;f__Hygrophoraceae;g__Hygrocybe;s__Hygrocybe_mucronella |
| OTU1332 | k__Fungi;p__Basidiomycota;c__Agaricomycetes;o__Agaricales;f__Hygrophoraceae;g__Hygrocybe;s__Hygrocybe_subpapillata |
| OTU556 | k__Fungi;p__Basidiomycota;c__Agaricomycetes;o__Agaricales;f__Hygrophoraceae;g__Hygrocybe;s__unidentified |
| OTU1267 | k__Fungi;p__Basidiomycota;c__Agaricomycetes;o__Agaricales;f__Hymenogastraceae;g__Anamika;s__Anamika_lactariolens |
| OTU53 | k__Fungi;p__Basidiomycota;c__Agaricomycetes;o__Agaricales;f__Hymenogastraceae;g__Hebeloma;s__Hebeloma_crustuliniforme |
| OTU603 | k__Fungi;p__Basidiomycota;c__Agaricomycetes;o__Agaricales;f__Hymenogastraceae;g__Hebeloma;s__Hebeloma_youngii |
| OTU1103 | k__Fungi;p__Basidiomycota;c__Agaricomycetes;o__Agaricales;f__Hymenogastraceae;g__Hebeloma;s__unidentified |
| OTU1266 | k__Fungi;p__Basidiomycota;c__Agaricomycetes;o__Agaricales;f__Inocybaceae;g__Inocybe;s__Inocybe_caroticolor |
| OTU96 | k__Fungi;p__Basidiomycota;c__Agaricomycetes;o__Agaricales;f__Inocybaceae;g__Inocybe;s__Inocybe_changbaiensis |
| OTU968 | k__Fungi;p__Basidiomycota;c__Agaricomycetes;o__Agaricales;f__Inocybaceae;g__Inocybe;s__Inocybe_furfurea |
| OTU945 | k__Fungi;p__Basidiomycota;c__Agaricomycetes;o__Agaricales;f__Inocybaceae;g__Inocybe;s__Inocybe_phaeodisca |
| OTU146 | k__Fungi;p__Basidiomycota;c__Agaricomycetes;o__Agaricales;f__Inocybaceae;g__Inocybe;s__Inocybe_rhodella |
| OTU178 | k__Fungi;p__Basidiomycota;c__Agaricomycetes;o__Agaricales;f__Inocybaceae;g__Inocybe;s__Inocybe_stellata |
| OTU54 | k__Fungi;p__Basidiomycota;c__Agaricomycetes;o__Agaricales;f__Inocybaceae;g__Inocybe;s__unidentified |
| OTU127 | k__Fungi;p__Basidiomycota;c__Agaricomycetes;o__Agaricales;f__Inocybaceae;g__Inocybe;s__unidentified |
| OTU202 | k__Fungi;p__Basidiomycota;c__Agaricomycetes;o__Agaricales;f__Inocybaceae;g__Inocybe;s__unidentified |
| OTU360 | k__Fungi;p__Basidiomycota;c__Agaricomycetes;o__Agaricales;f__Inocybaceae;g__Inocybe;s__unidentified |
| OTU768 | k__Fungi;p__Basidiomycota;c__Agaricomycetes;o__Agaricales;f__Inocybaceae;g__Inocybe;s__unidentified |
| OTU1233 | k__Fungi;p__Basidiomycota;c__Agaricomycetes;o__Agaricales;f__Lycoperdaceae;g__Lycoperdon;s__Lycoperdon_molle |
| OTU740 | k__Fungi;p__Basidiomycota;c__Agaricomycetes;o__Agaricales;f__Lyophyllaceae;g__Fibulochlamys;s__Fibulochlamys_chilensis |
| OTU1008 | k__Fungi;p__Basidiomycota;c__Agaricomycetes;o__Agaricales;f__Lyophyllaceae;g__Lyophyllum;s__unidentified |
| OTU1019 | k__Fungi;p__Basidiomycota;c__Agaricomycetes;o__Agaricales;f__Lyophyllaceae;g__Tephrocybe;s__Tephrocybe_rancida |
| OTU617 | k__Fungi;p__Basidiomycota;c__Agaricomycetes;o__Agaricales;f__Lyophyllaceae;g__Tephrocybe;s__unidentified |
| OTU29 | k__Fungi;p__Basidiomycota;c__Agaricomycetes;o__Agaricales;f__Lyophyllaceae;g__Termitomyces;s__Termitomyces_eurrhizus |
| OTU141 | k__Fungi;p__Basidiomycota;c__Agaricomycetes;o__Agaricales;f__Lyophyllaceae;g__Termitomyces;s__Termitomyces_microcarpus |
| OTU419 | k__Fungi;p__Basidiomycota;c__Agaricomycetes;o__Agaricales;f__Lyophyllaceae;g__Termitomyces;s__unidentified |
| OTU1168 | k__Fungi;p__Basidiomycota;c__Agaricomycetes;o__Agaricales;f__Lyophyllaceae;g__unidentified;s__unidentified |
| OTU767 | k__Fungi;p__Basidiomycota;c__Agaricomycetes;o__Agaricales;f__Omphalotaceae;g__Gymnopus;s__Gymnopus_dryophilus |
| OTU113 | k__Fungi;p__Basidiomycota;c__Agaricomycetes;o__Agaricales;f__Omphalotaceae;g__Gymnopus;s__Gymnopus_melanopus |
| OTU922 | k__Fungi;p__Basidiomycota;c__Agaricomycetes;o__Agaricales;f__Physalacriaceae;g__Mucidula;s__Mucidula_mucida |
| OTU1223 | k__Fungi;p__Basidiomycota;c__Agaricomycetes;o__Agaricales;f__Physalacriaceae;g__Mycotribulus;s__Mycotribulus_indonesiae |
| OTU1072 | k__Fungi;p__Basidiomycota;c__Agaricomycetes;o__Agaricales;f__Physalacriaceae;g__unidentified;s__unidentified |
| OTU293 | k__Fungi;p__Basidiomycota;c__Agaricomycetes;o__Agaricales;f__Pleurotaceae;g__Pleurotus;s__Pleurotus_cystidiosus |
| OTU65 | k__Fungi;p__Basidiomycota;c__Agaricomycetes;o__Agaricales;f__Pleurotaceae;g__unidentified;s__unidentified |
| OTU506 | k__Fungi;p__Basidiomycota;c__Agaricomycetes;o__Agaricales;f__Pluteaceae;g__Pluteus;s__Pluteus_diettrichii |
| OTU1089 | k__Fungi;p__Basidiomycota;c__Agaricomycetes;o__Agaricales;f__Pluteaceae;g__Pluteus;s__Pluteus_multiformis |
| OTU1306 | k__Fungi;p__Basidiomycota;c__Agaricomycetes;o__Agaricales;f__Pluteaceae;g__Pluteus;s__Pluteus_septocystidiatus |
| OTU1015 | k__Fungi;p__Basidiomycota;c__Agaricomycetes;o__Agaricales;f__Porotheleaceae;g__Porotheleum;s__Porotheleum_fimbriatum |
| OTU562 | k__Fungi;p__Basidiomycota;c__Agaricomycetes;o__Agaricales;f__Psathyrellaceae;g__Coprinellus;s__Coprinellus_aureogranulatus |
| OTU236 | k__Fungi;p__Basidiomycota;c__Agaricomycetes;o__Agaricales;f__Psathyrellaceae;g__Coprinellus;s__Coprinellus_disseminatus |
| OTU1021 | k__Fungi;p__Basidiomycota;c__Agaricomycetes;o__Agaricales;f__Psathyrellaceae;g__Coprinellus;s__Coprinellus_heptemerus |
| OTU1323 | k__Fungi;p__Basidiomycota;c__Agaricomycetes;o__Agaricales;f__Psathyrellaceae;g__Coprinellus;s__Coprinellus_micaceus |
| OTU1211 | k__Fungi;p__Basidiomycota;c__Agaricomycetes;o__Agaricales;f__Psathyrellaceae;g__Coprinellus;s__Coprinellus_velatopruinatus |
| OTU841 | k__Fungi;p__Basidiomycota;c__Agaricomycetes;o__Agaricales;f__Psathyrellaceae;g__Coprinellus;s__Coprinellus_verrucispermus |
| OTU268 | k__Fungi;p__Basidiomycota;c__Agaricomycetes;o__Agaricales;f__Psathyrellaceae;g__Coprinopsis;s__Coprinopsis_phlyctidospora |
| OTU72 | k__Fungi;p__Basidiomycota;c__Agaricomycetes;o__Agaricales;f__Psathyrellaceae;g__Coprinopsis;s__Coprinopsis_spelaiophila |
| OTU445 | k__Fungi;p__Basidiomycota;c__Agaricomycetes;o__Agaricales;f__Psathyrellaceae;g__Coprinopsis;s__unidentified |
| OTU548 | k__Fungi;p__Basidiomycota;c__Agaricomycetes;o__Agaricales;f__Psathyrellaceae;g__Lacrymaria;s__Lacrymaria_lacrymabunda |
| OTU62 | k__Fungi;p__Basidiomycota;c__Agaricomycetes;o__Agaricales;f__Psathyrellaceae;g__Parasola;s__Parasola_setulosa |
| OTU578 | k__Fungi;p__Basidiomycota;c__Agaricomycetes;o__Agaricales;f__Psathyrellaceae;g__Psathyrella;s__Psathyrella_candolleana |
| OTU846 | k__Fungi;p__Basidiomycota;c__Agaricomycetes;o__Agaricales;f__Psathyrellaceae;g__Psathyrella;s__Psathyrella_efflorescens |
| OTU992 | k__Fungi;p__Basidiomycota;c__Agaricomycetes;o__Agaricales;f__Psathyrellaceae;g__Psathyrella;s__Psathyrella_fagetophila |
| OTU160 | k__Fungi;p__Basidiomycota;c__Agaricomycetes;o__Agaricales;f__Psathyrellaceae;g__Psathyrella;s__Psathyrella_maculata |
| OTU1170 | k__Fungi;p__Basidiomycota;c__Agaricomycetes;o__Agaricales;f__Psathyrellaceae;g__Psathyrella;s__Psathyrella_madida |
| OTU1282 | k__Fungi;p__Basidiomycota;c__Agaricomycetes;o__Agaricales;f__Psathyrellaceae;g__Psathyrella;s__Psathyrella_magnispora |
| OTU557 | k__Fungi;p__Basidiomycota;c__Agaricomycetes;o__Agaricales;f__Psathyrellaceae;g__unidentified;s__unidentified |
| OTU125 | k__Fungi;p__Basidiomycota;c__Agaricomycetes;o__Agaricales;f__Strophariaceae;g__Galerina;s__Galerina_marginata |
| OTU1250 | k__Fungi;p__Basidiomycota;c__Agaricomycetes;o__Agaricales;f__Strophariaceae;g__Hypholoma;s__Hypholoma_fasciculare |
| OTU959 | k__Fungi;p__Basidiomycota;c__Agaricomycetes;o__Agaricales;f__Strophariaceae;g__Pholiota;s__unidentified |
| OTU688 | k__Fungi;p__Basidiomycota;c__Agaricomycetes;o__Agaricales;f__Strophariaceae;g__Psilocybe;s__Psilocybe_coprophila |
| OTU677 | k__Fungi;p__Basidiomycota;c__Agaricomycetes;o__Agaricales;f__Strophariaceae;g__unidentified;s__unidentified |
| OTU107 | k__Fungi;p__Basidiomycota;c__Agaricomycetes;o__Agaricales;f__Tricholomataceae;g__Atractosporocybe;s__Atractosporocybe_inornata |
| OTU854 | k__Fungi;p__Basidiomycota;c__Agaricomycetes;o__Agaricales;f__Tricholomataceae;g__Delicatula;s__Delicatula_integrella |
| OTU899 | k__Fungi;p__Basidiomycota;c__Agaricomycetes;o__Agaricales;f__Tricholomataceae;g__Hemimycena;s__Hemimycena_angustispora |
| OTU326 | k__Fungi;p__Basidiomycota;c__Agaricomycetes;o__Agaricales;f__Tricholomataceae;g__Mycena;s__Mycena_adscendens |
| OTU1285 | k__Fungi;p__Basidiomycota;c__Agaricomycetes;o__Agaricales;f__Tricholomataceae;g__Mycena;s__Mycena_pearsoniana |
| OTU753 | k__Fungi;p__Basidiomycota;c__Agaricomycetes;o__Agaricales;f__Tricholomataceae;g__Mycena;s__unidentified |
| OTU139 | k__Fungi;p__Basidiomycota;c__Agaricomycetes;o__Agaricales;f__Tricholomataceae;g__Singerocybe;s__Singerocybe_alboinfundibuliformis |
| OTU1184 | k__Fungi;p__Basidiomycota;c__Agaricomycetes;o__Agaricales;f__Tricholomataceae;g__Tricholoma;s__Tricholoma_equestre |
| OTU283 | k__Fungi;p__Basidiomycota;c__Agaricomycetes;o__Agaricales;f__Tricholomataceae;g__Tricholomopsis;s__unidentified |
| OTU30 | k__Fungi;p__Basidiomycota;c__Agaricomycetes;o__Agaricales;f__unidentified;g__unidentified;s__unidentified |
| OTU263 | k__Fungi;p__Basidiomycota;c__Agaricomycetes;o__Agaricales;f__unidentified;g__unidentified;s__unidentified |
| OTU378 | k__Fungi;p__Basidiomycota;c__Agaricomycetes;o__Agaricales;f__unidentified;g__unidentified;s__unidentified |
| OTU613 | k__Fungi;p__Basidiomycota;c__Agaricomycetes;o__Atheliales;f__Atheliaceae;g__Amphinema;s__unidentified |
| OTU1108 | k__Fungi;p__Basidiomycota;c__Agaricomycetes;o__Atheliales;f__Atheliaceae;g__Byssocorticium;s__unidentified |
| OTU1251 | k__Fungi;p__Basidiomycota;c__Agaricomycetes;o__Atheliales;f__unidentified;g__unidentified;s__unidentified |
| OTU424 | k__Fungi;p__Basidiomycota;c__Agaricomycetes;o__Auriculariales;f__Auriculariaceae;g__Auricularia;s__unidentified |
| OTU558 | k__Fungi;p__Basidiomycota;c__Agaricomycetes;o__Auriculariales;f__Exidiaceae;g__Exidia;s__Exidia_pithya |
| OTU221 | k__Fungi;p__Basidiomycota;c__Agaricomycetes;o__Auriculariales;f__Exidiaceae;g__unidentified;s__unidentified |
| OTU1189 | k__Fungi;p__Basidiomycota;c__Agaricomycetes;o__Auriculariales;f__Hyaloriaceae;g__Protodontia;s__Protodontia_piceicola |
| OTU372 | k__Fungi;p__Basidiomycota;c__Agaricomycetes;o__Auriculariales;f__unidentified;g__unidentified;s__unidentified |
| OTU1269 | k__Fungi;p__Basidiomycota;c__Agaricomycetes;o__Boletales;f__Boletaceae;g__Boletus;s__Boletus_monilifer |
| OTU719 | k__Fungi;p__Basidiomycota;c__Agaricomycetes;o__Boletales;f__Boletaceae;g__Boletus;s__Boletus_violaceofuscus |
| OTU254 | k__Fungi;p__Basidiomycota;c__Agaricomycetes;o__Boletales;f__Boletaceae;g__Heimioporus;s__Heimioporus_japonicus |
| OTU486 | k__Fungi;p__Basidiomycota;c__Agaricomycetes;o__Boletales;f__Boletaceae;g__Mycoamaranthus;s__Mycoamaranthus_cambodgensis |
| OTU1242 | k__Fungi;p__Basidiomycota;c__Agaricomycetes;o__Boletales;f__Boletaceae;g__Phylloporus;s__unidentified |
| OTU1049 | k__Fungi;p__Basidiomycota;c__Agaricomycetes;o__Boletales;f__Boletaceae;g__Strobilomyces;s__Strobilomyces_echinocephalus |
| OTU857 | k__Fungi;p__Basidiomycota;c__Agaricomycetes;o__Boletales;f__Boletaceae;g__Tylopilus;s__Tylopilus_vinosobrunneus |
| OTU411 | k__Fungi;p__Basidiomycota;c__Agaricomycetes;o__Boletales;f__Boletaceae;g__unidentified;s__unidentified |
| OTU1232 | k__Fungi;p__Basidiomycota;c__Agaricomycetes;o__Boletales;f__Coniophoraceae;g__Coniophora;s__Coniophora_hanoiensis |
| OTU245 | k__Fungi;p__Basidiomycota;c__Agaricomycetes;o__Boletales;f__Coniophoraceae;g__Coniophora;s__Coniophora_puteana |
| OTU865 | k__Fungi;p__Basidiomycota;c__Agaricomycetes;o__Boletales;f__Coniophoraceae;g__Gyrodontium;s__Gyrodontium_sacchari |
| OTU420 | k__Fungi;p__Basidiomycota;c__Agaricomycetes;o__Boletales;f__Gyrodontaceae;g__Gyrodon;s__unidentified |
| OTU388 | k__Fungi;p__Basidiomycota;c__Agaricomycetes;o__Boletales;f__Gyroporaceae;g__Gyroporus;s__Gyroporus_lacteus |
| OTU1186 | k__Fungi;p__Basidiomycota;c__Agaricomycetes;o__Boletales;f__Gyroporaceae;g__Gyroporus;s__unidentified |
| OTU1055 | k__Fungi;p__Basidiomycota;c__Agaricomycetes;o__Boletales;f__Pisolithaceae;g__Pisolithus;s__Pisolithus_orientalis |
| OTU1101 | k__Fungi;p__Basidiomycota;c__Agaricomycetes;o__Boletales;f__Rhizopogonaceae;g__Rhizopogon;s__Rhizopogon_flavidus |
| OTU1165 | k__Fungi;p__Basidiomycota;c__Agaricomycetes;o__Boletales;f__Rhizopogonaceae;g__Rhizopogon;s__Rhizopogon_jiyaozi |
| OTU997 | k__Fungi;p__Basidiomycota;c__Agaricomycetes;o__Boletales;f__Rhizopogonaceae;g__Rhizopogon;s__unidentified |
| OTU1194 | k__Fungi;p__Basidiomycota;c__Agaricomycetes;o__Boletales;f__Sclerodermataceae;g__Scleroderma;s__Scleroderma_capeverdeanum |
| OTU1056 | k__Fungi;p__Basidiomycota;c__Agaricomycetes;o__Boletales;f__Sclerodermataceae;g__Scleroderma;s__Scleroderma_columnare |
| OTU1177 | k__Fungi;p__Basidiomycota;c__Agaricomycetes;o__Boletales;f__Sclerodermataceae;g__Scleroderma;s__Scleroderma_laeve |
| OTU1182 | k__Fungi;p__Basidiomycota;c__Agaricomycetes;o__Boletales;f__Sclerodermataceae;g__Scleroderma;s__Scleroderma_sinnamariense |
| OTU1138 | k__Fungi;p__Basidiomycota;c__Agaricomycetes;o__Boletales;f__Sclerodermataceae;g__Scleroderma;s__Scleroderma_yunnanense |
| OTU503 | k__Fungi;p__Basidiomycota;c__Agaricomycetes;o__Boletales;f__Sclerodermataceae;g__Scleroderma;s__unidentified |
| OTU600 | k__Fungi;p__Basidiomycota;c__Agaricomycetes;o__Boletales;f__Serpulaceae;g__Serpula;s__Serpula_dendrocalami |
| OTU406 | k__Fungi;p__Basidiomycota;c__Agaricomycetes;o__Boletales;f__Suillaceae;g__Suillus;s__Suillus_cavipes |
| OTU1319 | k__Fungi;p__Basidiomycota;c__Agaricomycetes;o__Boletales;f__Tapinellaceae;g__Pseudomerulius;s__Pseudomerulius_curtisii |
| OTU650 | k__Fungi;p__Basidiomycota;c__Agaricomycetes;o__Boletales;f__Tapinellaceae;g__Tapinella;s__Tapinella_panuoides |
| OTU498 | k__Fungi;p__Basidiomycota;c__Agaricomycetes;o__Cantharellales;f__Botryobasidiaceae;g__Botryobasidium;s__Botryobasidium_obtusisporum |
| OTU547 | k__Fungi;p__Basidiomycota;c__Agaricomycetes;o__Cantharellales;f__Botryobasidiaceae;g__Botryobasidium;s__unidentified |
| OTU991 | k__Fungi;p__Basidiomycota;c__Agaricomycetes;o__Cantharellales;f__Botryobasidiaceae;g__unidentified;s__unidentified |
| OTU1191 | k__Fungi;p__Basidiomycota;c__Agaricomycetes;o__Cantharellales;f__Cantharellales_fam_Incertae_sedis;g__Minimedusa;s__Minimedusa_polyspora |
| OTU534 | k__Fungi;p__Basidiomycota;c__Agaricomycetes;o__Cantharellales;f__Cantharellales_fam_Incertae_sedis;g__Sistotrema;s__unidentified |
| OTU807 | k__Fungi;p__Basidiomycota;c__Agaricomycetes;o__Cantharellales;f__Ceratobasidiaceae;g__Ceratobasidium;s__unidentified |
| OTU243 | k__Fungi;p__Basidiomycota;c__Agaricomycetes;o__Cantharellales;f__Ceratobasidiaceae;g__unidentified;s__unidentified |
| OTU926 | k__Fungi;p__Basidiomycota;c__Agaricomycetes;o__Cantharellales;f__Clavulinaceae;g__Clavulina;s__Clavulina_reae |
| OTU315 | k__Fungi;p__Basidiomycota;c__Agaricomycetes;o__Cantharellales;f__Clavulinaceae;g__Clavulina;s__Clavulina_rugosa |
| OTU641 | k__Fungi;p__Basidiomycota;c__Agaricomycetes;o__Cantharellales;f__Clavulinaceae;g__Clavulina;s__unidentified |
| OTU304 | k__Fungi;p__Basidiomycota;c__Agaricomycetes;o__Cantharellales;f__Hydnaceae;g__Hydnum;s__Hydnum_albomagnum |
| OTU222 | k__Fungi;p__Basidiomycota;c__Agaricomycetes;o__Corticiales;f__Corticiaceae;g__Corticium;s__unidentified |
| OTU765 | k__Fungi;p__Basidiomycota;c__Agaricomycetes;o__Corticiales;f__Punctulariaceae;g__Punctularia;s__Punctularia_atropurpurascens |
| OTU457 | k__Fungi;p__Basidiomycota;c__Agaricomycetes;o__Corticiales;f__Vuilleminiaceae;g__Vuilleminia;s__Vuilleminia_comedens |
| OTU1063 | k__Fungi;p__Basidiomycota;c__Agaricomycetes;o__Geastrales;f__Geastraceae;g__Geastrum;s__Geastrum_albonigrum |
| OTU1096 | k__Fungi;p__Basidiomycota;c__Agaricomycetes;o__Geastrales;f__Geastraceae;g__Geastrum;s__Geastrum_mirabile |
| OTU138 | k__Fungi;p__Basidiomycota;c__Agaricomycetes;o__Geastrales;f__Geastraceae;g__Geastrum;s__unidentified |
| OTU258 | k__Fungi;p__Basidiomycota;c__Agaricomycetes;o__Geastrales;f__Geastraceae;g__Geastrum;s__unidentified |
| OTU286 | k__Fungi;p__Basidiomycota;c__Agaricomycetes;o__Geastrales;f__Sphaerobolaceae;g__Sphaerobolus;s__unidentified |
| OTU395 | k__Fungi;p__Basidiomycota;c__Agaricomycetes;o__Gomphales;f__Gomphaceae;g__Ramaria;s__unidentified |
| OTU373 | k__Fungi;p__Basidiomycota;c__Agaricomycetes;o__Hymenochaetales;f__Hymenochaetaceae;g__Fomitiporella;s__unidentified |
| OTU312 | k__Fungi;p__Basidiomycota;c__Agaricomycetes;o__Hymenochaetales;f__Hymenochaetaceae;g__Hydnochaete;s__Hydnochaete_japonica |
| OTU152 | k__Fungi;p__Basidiomycota;c__Agaricomycetes;o__Hymenochaetales;f__Hymenochaetaceae;g__Hymenochaete;s__Hymenochaete_innexa |
| OTU1052 | k__Fungi;p__Basidiomycota;c__Agaricomycetes;o__Hymenochaetales;f__Hymenochaetaceae;g__Hymenochaete;s__Hymenochaete_sphaerospora |
| OTU273 | k__Fungi;p__Basidiomycota;c__Agaricomycetes;o__Hymenochaetales;f__Hymenochaetaceae;g__Phellinus;s__Phellinus_gilvus |
| OTU150 | k__Fungi;p__Basidiomycota;c__Agaricomycetes;o__Hymenochaetales;f__Hymenochaetaceae;g__unidentified;s__unidentified |
| OTU517 | k__Fungi;p__Basidiomycota;c__Agaricomycetes;o__Hymenochaetales;f__Hymenochaetales_fam_Incertae_sedis;g__Peniophorella;s__Peniophorella_praetermissa |
| OTU1193 | k__Fungi;p__Basidiomycota;c__Agaricomycetes;o__Hymenochaetales;f__Schizoporaceae;g__Hyphodontia;s__Hyphodontia_crustosa |
| OTU483 | k__Fungi;p__Basidiomycota;c__Agaricomycetes;o__Hymenochaetales;f__Schizoporaceae;g__Hyphodontia;s__Hyphodontia_heterocystidiata |
| OTU1329 | k__Fungi;p__Basidiomycota;c__Agaricomycetes;o__Hymenochaetales;f__Schizoporaceae;g__Hyphodontia;s__Hyphodontia_rhizomorpha |
| OTU560 | k__Fungi;p__Basidiomycota;c__Agaricomycetes;o__Hymenochaetales;f__Schizoporaceae;g__unidentified;s__unidentified |
| OTU618 | k__Fungi;p__Basidiomycota;c__Agaricomycetes;o__Hysterangiales;f__Hysterangiaceae;g__Chondrogaster;s__unidentified |
| OTU153 | k__Fungi;p__Basidiomycota;c__Agaricomycetes;o__Phallales;f__Phallaceae;g__Phallus;s__Phallus_haitangensis |
| OTU1098 | k__Fungi;p__Basidiomycota;c__Agaricomycetes;o__Polyporales;f__Cerrenaceae;g__Pseudolagarobasidium;s__Pseudolagarobasidium_acaciicola |
| OTU118 | k__Fungi;p__Basidiomycota;c__Agaricomycetes;o__Polyporales;f__Cerrenaceae;g__Radulodon;s__Radulodon_casearius |
| OTU1320 | k__Fungi;p__Basidiomycota;c__Agaricomycetes;o__Polyporales;f__Fomitopsidaceae;g__Antrodia;s__Antrodia_neotropica |
| OTU1045 | k__Fungi;p__Basidiomycota;c__Agaricomycetes;o__Polyporales;f__Fomitopsidaceae;g__Fibroporia;s__Fibroporia_albicans |
| OTU1321 | k__Fungi;p__Basidiomycota;c__Agaricomycetes;o__Polyporales;f__Fomitopsidaceae;g__Fibroporia;s__Fibroporia_citrina |
| OTU1145 | k__Fungi;p__Basidiomycota;c__Agaricomycetes;o__Polyporales;f__Fomitopsidaceae;g__Postia;s__Postia_gloeopora |
| OTU1292 | k__Fungi;p__Basidiomycota;c__Agaricomycetes;o__Polyporales;f__Fomitopsidaceae;g__Skeletocutis;s__unidentified |
| OTU1051 | k__Fungi;p__Basidiomycota;c__Agaricomycetes;o__Polyporales;f__Fomitopsidaceae;g__unidentified;s__unidentified |
| OTU1026 | k__Fungi;p__Basidiomycota;c__Agaricomycetes;o__Polyporales;f__Ganodermataceae;g__Ganoderma;s__Ganoderma_angustisporum |
| OTU310 | k__Fungi;p__Basidiomycota;c__Agaricomycetes;o__Polyporales;f__Ganodermataceae;g__Ganoderma;s__Ganoderma_applanatum |
| OTU936 | k__Fungi;p__Basidiomycota;c__Agaricomycetes;o__Polyporales;f__Ganodermataceae;g__Ganoderma;s__Ganoderma_australe |
| OTU1198 | k__Fungi;p__Basidiomycota;c__Agaricomycetes;o__Polyporales;f__Ganodermataceae;g__Ganoderma;s__Ganoderma_calidophilum |
| OTU852 | k__Fungi;p__Basidiomycota;c__Agaricomycetes;o__Polyporales;f__Ganodermataceae;g__Ganoderma;s__Ganoderma_enigmaticum |
| OTU789 | k__Fungi;p__Basidiomycota;c__Agaricomycetes;o__Polyporales;f__Ganodermataceae;g__Ganoderma;s__Ganoderma_flexipes |
| OTU1221 | k__Fungi;p__Basidiomycota;c__Agaricomycetes;o__Polyporales;f__Ganodermataceae;g__Ganoderma;s__Ganoderma_hoehnelianum |
| OTU816 | k__Fungi;p__Basidiomycota;c__Agaricomycetes;o__Polyporales;f__Ganodermataceae;g__Ganoderma;s__Ganoderma_lucidum |
| OTU679 | k__Fungi;p__Basidiomycota;c__Agaricomycetes;o__Polyporales;f__Ganodermataceae;g__Ganoderma;s__Ganoderma_mbrekobenum |
| OTU967 | k__Fungi;p__Basidiomycota;c__Agaricomycetes;o__Polyporales;f__Ganodermataceae;g__Ganoderma;s__Ganoderma_multiplicatum |
| OTU842 | k__Fungi;p__Basidiomycota;c__Agaricomycetes;o__Polyporales;f__Ganodermataceae;g__Ganoderma;s__Ganoderma_orbiforme |
| OTU172 | k__Fungi;p__Basidiomycota;c__Agaricomycetes;o__Polyporales;f__Ganodermataceae;g__Ganoderma;s__Ganoderma_sichuanense |
| OTU880 | k__Fungi;p__Basidiomycota;c__Agaricomycetes;o__Polyporales;f__Ganodermataceae;g__Ganoderma;s__Ganoderma_tropicum |
| OTU541 | k__Fungi;p__Basidiomycota;c__Agaricomycetes;o__Polyporales;f__Ganodermataceae;g__Ganoderma;s__unidentified |
| OTU337 | k__Fungi;p__Basidiomycota;c__Agaricomycetes;o__Polyporales;f__Ganodermataceae;g__Perenniporia;s__Perenniporia_martia |
| OTU392 | k__Fungi;p__Basidiomycota;c__Agaricomycetes;o__Polyporales;f__Ganodermataceae;g__unidentified;s__unidentified |
| OTU455 | k__Fungi;p__Basidiomycota;c__Agaricomycetes;o__Polyporales;f__Hyphodermataceae;g__Hyphoderma;s__unidentified |
| OTU343 | k__Fungi;p__Basidiomycota;c__Agaricomycetes;o__Polyporales;f__Irpicaceae;g__Ceriporia;s__Ceriporia_lacerata |
| OTU1273 | k__Fungi;p__Basidiomycota;c__Agaricomycetes;o__Polyporales;f__Irpicaceae;g__Gloeoporus;s__unidentified |
| OTU322 | k__Fungi;p__Basidiomycota;c__Agaricomycetes;o__Polyporales;f__Meruliaceae;g__Luteoporia;s__Luteoporia_albomarginata |
| OTU164 | k__Fungi;p__Basidiomycota;c__Agaricomycetes;o__Polyporales;f__Meruliaceae;g__Phlebia;s__Phlebia_sp |
| OTU870 | k__Fungi;p__Basidiomycota;c__Agaricomycetes;o__Polyporales;f__Meruliaceae;g__Phlebia;s__Phlebia_tuberculata |
| OTU844 | k__Fungi;p__Basidiomycota;c__Agaricomycetes;o__Polyporales;f__Phanerochaetaceae;g__Hyphodermella;s__Hyphodermella_rosae |
| OTU1289 | k__Fungi;p__Basidiomycota;c__Agaricomycetes;o__Polyporales;f__Phanerochaetaceae;g__Phanerochaete;s__Phanerochaete_bambusicola |
| OTU1290 | k__Fungi;p__Basidiomycota;c__Agaricomycetes;o__Polyporales;f__Phanerochaetaceae;g__Phanerochaete;s__Phanerochaete_citri |
| OTU1235 | k__Fungi;p__Basidiomycota;c__Agaricomycetes;o__Polyporales;f__Phanerochaetaceae;g__Phanerochaete;s__Phanerochaete_concrescens |
| OTU1207 | k__Fungi;p__Basidiomycota;c__Agaricomycetes;o__Polyporales;f__Phanerochaetaceae;g__Phanerochaete;s__Phanerochaete_cumulodentata |
| OTU549 | k__Fungi;p__Basidiomycota;c__Agaricomycetes;o__Polyporales;f__Phanerochaetaceae;g__Phanerochaete;s__Phanerochaete_incarnata |
| OTU1000 | k__Fungi;p__Basidiomycota;c__Agaricomycetes;o__Polyporales;f__Phanerochaetaceae;g__Phanerochaete;s__Phanerochaete_sordida |
| OTU1147 | k__Fungi;p__Basidiomycota;c__Agaricomycetes;o__Polyporales;f__Phanerochaetaceae;g__Phanerochaete;s__unidentified |
| OTU1041 | k__Fungi;p__Basidiomycota;c__Agaricomycetes;o__Polyporales;f__Phanerochaetaceae;g__Phlebiopsis;s__unidentified |
| OTU974 | k__Fungi;p__Basidiomycota;c__Agaricomycetes;o__Polyporales;f__Phanerochaetaceae;g__Porostereum;s__Porostereum_spadiceum |
| OTU883 | k__Fungi;p__Basidiomycota;c__Agaricomycetes;o__Polyporales;f__Polyporaceae;g__Coriolopsis;s__Coriolopsis_caperata |
| OTU539 | k__Fungi;p__Basidiomycota;c__Agaricomycetes;o__Polyporales;f__Polyporaceae;g__Daedaleopsis;s__Daedaleopsis_confragosa |
| OTU575 | k__Fungi;p__Basidiomycota;c__Agaricomycetes;o__Polyporales;f__Polyporaceae;g__Dichomitus;s__Dichomitus_squalens |
| OTU1236 | k__Fungi;p__Basidiomycota;c__Agaricomycetes;o__Polyporales;f__Polyporaceae;g__Dichomitus;s__unidentified |
| OTU184 | k__Fungi;p__Basidiomycota;c__Agaricomycetes;o__Polyporales;f__Polyporaceae;g__Earliella;s__Earliella_scabrosa |
| OTU555 | k__Fungi;p__Basidiomycota;c__Agaricomycetes;o__Polyporales;f__Polyporaceae;g__Epithele;s__Epithele_typhae |
| OTU528 | k__Fungi;p__Basidiomycota;c__Agaricomycetes;o__Polyporales;f__Polyporaceae;g__Favolus;s__Favolus_pseudoemerici |
| OTU281 | k__Fungi;p__Basidiomycota;c__Agaricomycetes;o__Polyporales;f__Polyporaceae;g__Favolus;s__Favolus_tenuiculus |
| OTU1305 | k__Fungi;p__Basidiomycota;c__Agaricomycetes;o__Polyporales;f__Polyporaceae;g__Grammothele;s__Grammothele_lineata |
| OTU79 | k__Fungi;p__Basidiomycota;c__Agaricomycetes;o__Polyporales;f__Polyporaceae;g__Lentinus;s__Lentinus_squarrosulus |
| OTU1176 | k__Fungi;p__Basidiomycota;c__Agaricomycetes;o__Polyporales;f__Polyporaceae;g__Lenzites;s__Lenzites_warnieri |
| OTU1317 | k__Fungi;p__Basidiomycota;c__Agaricomycetes;o__Polyporales;f__Polyporaceae;g__Microporus;s__Microporus_affinis |
| OTU1082 | k__Fungi;p__Basidiomycota;c__Agaricomycetes;o__Polyporales;f__Polyporaceae;g__Trametes;s__Trametes_hirsuta |
| OTU285 | k__Fungi;p__Basidiomycota;c__Agaricomycetes;o__Polyporales;f__Polyporaceae;g__unidentified;s__unidentified |
| OTU349 | k__Fungi;p__Basidiomycota;c__Agaricomycetes;o__Polyporales;f__Polyporaceae;g__unidentified;s__unidentified |
| OTU917 | k__Fungi;p__Basidiomycota;c__Agaricomycetes;o__Polyporales;f__Steccherinaceae;g__Ceriporiopsis;s__Ceriporiopsis_carnegieae |
| OTU1246 | k__Fungi;p__Basidiomycota;c__Agaricomycetes;o__Polyporales;f__Steccherinaceae;g__Ceriporiopsis;s__Ceriporiopsis_semisupina |
| OTU1341 | k__Fungi;p__Basidiomycota;c__Agaricomycetes;o__Polyporales;f__Steccherinaceae;g__Steccherinum;s__Steccherinum_fimbriatum |
| OTU465 | k__Fungi;p__Basidiomycota;c__Agaricomycetes;o__Polyporales;f__Steccherinaceae;g__Steccherinum;s__Steccherinum_murashkinskyi |
| OTU627 | k__Fungi;p__Basidiomycota;c__Agaricomycetes;o__Polyporales;f__Steccherinaceae;g__Steccherinum;s__Steccherinum_neonitidum |
| OTU161 | k__Fungi;p__Basidiomycota;c__Agaricomycetes;o__Polyporales;f__Steccherinaceae;g__Steccherinum;s__Steccherinum_ochraceum |
| OTU12 | k__Fungi;p__Basidiomycota;c__Agaricomycetes;o__Polyporales;f__unidentified;g__unidentified;s__unidentified |
| OTU537 | k__Fungi;p__Basidiomycota;c__Agaricomycetes;o__Russulales;f__Albatrellaceae;g__Albatrellus;s__Albatrellus_caeruleoporus |
| OTU422 | k__Fungi;p__Basidiomycota;c__Agaricomycetes;o__Russulales;f__Albatrellaceae;g__Byssoporia;s__Byssoporia_terrestris |
| OTU783 | k__Fungi;p__Basidiomycota;c__Agaricomycetes;o__Russulales;f__Bondarzewiaceae;g__Heterobasidion;s__Heterobasidion_araucariae |
| OTU830 | k__Fungi;p__Basidiomycota;c__Agaricomycetes;o__Russulales;f__Lachnocladiaceae;g__Dichostereum;s__Dichostereum_boidinii |
| OTU121 | k__Fungi;p__Basidiomycota;c__Agaricomycetes;o__Russulales;f__Peniophoraceae;g__Peniophora;s__Peniophora_borbonica |
| OTU76 | k__Fungi;p__Basidiomycota;c__Agaricomycetes;o__Russulales;f__Russulaceae;g__Lactarius;s__Lactarius_furfuraceus |
| OTU665 | k__Fungi;p__Basidiomycota;c__Agaricomycetes;o__Russulales;f__Russulaceae;g__Lactarius;s__Lactarius_horakii |
| OTU1220 | k__Fungi;p__Basidiomycota;c__Agaricomycetes;o__Russulales;f__Russulaceae;g__Lactarius;s__Lactarius_laccarioides |
| OTU567 | k__Fungi;p__Basidiomycota;c__Agaricomycetes;o__Russulales;f__Russulaceae;g__Lactarius;s__Lactarius_perparvus |
| OTU1161 | k__Fungi;p__Basidiomycota;c__Agaricomycetes;o__Russulales;f__Russulaceae;g__Lactarius;s__Lactarius_pseudodelicatus |
| OTU1107 | k__Fungi;p__Basidiomycota;c__Agaricomycetes;o__Russulales;f__Russulaceae;g__Lactarius;s__unidentified |
| OTU412 | k__Fungi;p__Basidiomycota;c__Agaricomycetes;o__Russulales;f__Russulaceae;g__Lactifluus;s__Lactifluus_bicapillus |
| OTU307 | k__Fungi;p__Basidiomycota;c__Agaricomycetes;o__Russulales;f__Russulaceae;g__Lactifluus;s__Lactifluus_longipilus |
| OTU969 | k__Fungi;p__Basidiomycota;c__Agaricomycetes;o__Russulales;f__Russulaceae;g__Lactifluus;s__Lactifluus_pseudoluteopus |
| OTU467 | k__Fungi;p__Basidiomycota;c__Agaricomycetes;o__Russulales;f__Russulaceae;g__Lactifluus;s__Lactifluus_volemus |
| OTU416 | k__Fungi;p__Basidiomycota;c__Agaricomycetes;o__Russulales;f__Russulaceae;g__Lactifluus;s__unidentified |
| OTU216 | k__Fungi;p__Basidiomycota;c__Agaricomycetes;o__Russulales;f__Russulaceae;g__Russula;s__Russula_buyckii |
| OTU505 | k__Fungi;p__Basidiomycota;c__Agaricomycetes;o__Russulales;f__Russulaceae;g__Russula;s__Russula_catillus |
| OTU670 | k__Fungi;p__Basidiomycota;c__Agaricomycetes;o__Russulales;f__Russulaceae;g__Russula;s__Russula_compacta |
| OTU452 | k__Fungi;p__Basidiomycota;c__Agaricomycetes;o__Russulales;f__Russulaceae;g__Russula;s__Russula_cyanoxantha |
| OTU1255 | k__Fungi;p__Basidiomycota;c__Agaricomycetes;o__Russulales;f__Russulaceae;g__Russula;s__Russula_delica |
| OTU903 | k__Fungi;p__Basidiomycota;c__Agaricomycetes;o__Russulales;f__Russulaceae;g__Russula;s__Russula_densifolia |
| OTU1326 | k__Fungi;p__Basidiomycota;c__Agaricomycetes;o__Russulales;f__Russulaceae;g__Russula;s__Russula_earlei |
| OTU168 | k__Fungi;p__Basidiomycota;c__Agaricomycetes;o__Russulales;f__Russulaceae;g__Russula;s__Russula_emeticicolor |
| OTU777 | k__Fungi;p__Basidiomycota;c__Agaricomycetes;o__Russulales;f__Russulaceae;g__Russula;s__Russula_foetens |
| OTU1218 | k__Fungi;p__Basidiomycota;c__Agaricomycetes;o__Russulales;f__Russulaceae;g__Russula;s__Russula_livescens |
| OTU128 | k__Fungi;p__Basidiomycota;c__Agaricomycetes;o__Russulales;f__Russulaceae;g__Russula;s__Russula_mariae |
| OTU15 | k__Fungi;p__Basidiomycota;c__Agaricomycetes;o__Russulales;f__Russulaceae;g__Russula;s__Russula_pectinatoides |
| OTU421 | k__Fungi;p__Basidiomycota;c__Agaricomycetes;o__Russulales;f__Russulaceae;g__Russula;s__Russula_recondita |
| OTU1324 | k__Fungi;p__Basidiomycota;c__Agaricomycetes;o__Russulales;f__Russulaceae;g__Russula;s__Russula_saliceticola |
| OTU1142 | k__Fungi;p__Basidiomycota;c__Agaricomycetes;o__Russulales;f__Russulaceae;g__Russula;s__Russula_senecis |
| OTU1140 | k__Fungi;p__Basidiomycota;c__Agaricomycetes;o__Russulales;f__Russulaceae;g__Russula;s__Russula_virescens |
| OTU400 | k__Fungi;p__Basidiomycota;c__Agaricomycetes;o__Russulales;f__Russulaceae;g__Russula;s__unidentified |
| OTU576 | k__Fungi;p__Basidiomycota;c__Agaricomycetes;o__Russulales;f__Russulaceae;g__Russula;s__unidentified |
| OTU426 | k__Fungi;p__Basidiomycota;c__Agaricomycetes;o__Russulales;f__Russulaceae;g__unidentified;s__unidentified |
| OTU291 | k__Fungi;p__Basidiomycota;c__Agaricomycetes;o__Russulales;f__Stereaceae;g__Stereum;s__Stereum_sanguinolentum |
| OTU205 | k__Fungi;p__Basidiomycota;c__Agaricomycetes;o__Russulales;f__Wrightoporiaceae;g__Wrightoporia;s__Wrightoporia_biennis |
| OTU1260 | k__Fungi;p__Basidiomycota;c__Agaricomycetes;o__Sebacinales;f__Sebacinaceae;g__Chaetospermum;s__Chaetospermum_artocarpi |
| OTU300 | k__Fungi;p__Basidiomycota;c__Agaricomycetes;o__Sebacinales;f__Sebacinaceae;g__Chaetospermum;s__Chaetospermum_camelliae |
| OTU1044 | k__Fungi;p__Basidiomycota;c__Agaricomycetes;o__Sebacinales;f__Sebacinaceae;g__Helvellosebacina;s__unidentified |
| OTU17 | k__Fungi;p__Basidiomycota;c__Agaricomycetes;o__Sebacinales;f__Sebacinaceae;g__Sebacina;s__Sebacina_incrustans |
| OTU114 | k__Fungi;p__Basidiomycota;c__Agaricomycetes;o__Sebacinales;f__Sebacinaceae;g__Sebacina;s__Sebacina_sp |
| OTU346 | k__Fungi;p__Basidiomycota;c__Agaricomycetes;o__Sebacinales;f__Sebacinaceae;g__Sebacina;s__Sebacina_sp |
| OTU370 | k__Fungi;p__Basidiomycota;c__Agaricomycetes;o__Sebacinales;f__Sebacinaceae;g__unidentified;s__unidentified |
| OTU31 | k__Fungi;p__Basidiomycota;c__Agaricomycetes;o__Sebacinales;f__Serendipitaceae;g__Serendipita;s__unidentified |
| OTU203 | k__Fungi;p__Basidiomycota;c__Agaricomycetes;o__Sebacinales;f__Serendipitaceae;g__Serendipita;s__unidentified |
| OTU978 | k__Fungi;p__Basidiomycota;c__Agaricomycetes;o__Sebacinales;f__Serendipitaceae;g__Serendipita;s__unidentified |
| OTU450 | k__Fungi;p__Basidiomycota;c__Agaricomycetes;o__Sebacinales;f__Serendipitaceae;g__unidentified;s__Serendipitaceae_sp |
| OTU228 | k__Fungi;p__Basidiomycota;c__Agaricomycetes;o__Sebacinales;f__unidentified;g__unidentified;s__unidentified |
| OTU274 | k__Fungi;p__Basidiomycota;c__Agaricomycetes;o__Sebacinales;f__unidentified;g__unidentified;s__unidentified |
| OTU666 | k__Fungi;p__Basidiomycota;c__Agaricomycetes;o__Thelephorales;f__Thelephoraceae;g__Amaurodon;s__unidentified |
| OTU643 | k__Fungi;p__Basidiomycota;c__Agaricomycetes;o__Thelephorales;f__Thelephoraceae;g__Pseudotomentella;s__unidentified |
| OTU540 | k__Fungi;p__Basidiomycota;c__Agaricomycetes;o__Thelephorales;f__Thelephoraceae;g__Thelephora;s__unidentified |
| OTU663 | k__Fungi;p__Basidiomycota;c__Agaricomycetes;o__Thelephorales;f__Thelephoraceae;g__Tomentella;s__Tomentella_cinerascens |
| OTU906 | k__Fungi;p__Basidiomycota;c__Agaricomycetes;o__Thelephorales;f__Thelephoraceae;g__Tomentella;s__Tomentella_coerulea |
| OTU1152 | k__Fungi;p__Basidiomycota;c__Agaricomycetes;o__Thelephorales;f__Thelephoraceae;g__Tomentella;s__Tomentella_fuscocinerea |
| OTU410 | k__Fungi;p__Basidiomycota;c__Agaricomycetes;o__Thelephorales;f__Thelephoraceae;g__Tomentella;s__Tomentella_lapida |
| OTU828 | k__Fungi;p__Basidiomycota;c__Agaricomycetes;o__Thelephorales;f__Thelephoraceae;g__Tomentella;s__Tomentella_papuae |
| OTU1190 | k__Fungi;p__Basidiomycota;c__Agaricomycetes;o__Thelephorales;f__Thelephoraceae;g__Tomentella;s__Tomentella_pilosa |
| OTU573 | k__Fungi;p__Basidiomycota;c__Agaricomycetes;o__Thelephorales;f__Thelephoraceae;g__Tomentella;s__Tomentella_sp |
| OTU739 | k__Fungi;p__Basidiomycota;c__Agaricomycetes;o__Thelephorales;f__Thelephoraceae;g__Tomentella;s__Tomentella_subclavigera |
| OTU10 | k__Fungi;p__Basidiomycota;c__Agaricomycetes;o__Thelephorales;f__Thelephoraceae;g__Tomentella;s__unidentified |
| OTU701 | k__Fungi;p__Basidiomycota;c__Agaricomycetes;o__Thelephorales;f__Thelephoraceae;g__Tomentellopsis;s__unidentified |
| OTU97 | k__Fungi;p__Basidiomycota;c__Agaricomycetes;o__Thelephorales;f__Thelephoraceae;g__unidentified;s__unidentified |
| OTU244 | k__Fungi;p__Basidiomycota;c__Agaricomycetes;o__Thelephorales;f__Thelephoraceae;g__unidentified;s__unidentified |
| OTU1229 | k__Fungi;p__Basidiomycota;c__Agaricomycetes;o__Trechisporales;f__Hydnodontaceae;g__Subulicystidium;s__Subulicystidium_perlongisporum |
| OTU888 | k__Fungi;p__Basidiomycota;c__Agaricomycetes;o__Trechisporales;f__Hydnodontaceae;g__Subulicystidium;s__unidentified |
| OTU856 | k__Fungi;p__Basidiomycota;c__Agaricomycetes;o__Trechisporales;f__Hydnodontaceae;g__Trechispora;s__Trechispora_kavinioides |
| OTU930 | k__Fungi;p__Basidiomycota;c__Agaricomycetes;o__Trechisporales;f__Hydnodontaceae;g__Trechispora;s__Trechispora_nivea |
| OTU552 | k__Fungi;p__Basidiomycota;c__Agaricomycetes;o__Trechisporales;f__Hydnodontaceae;g__Trechispora;s__Trechispora_sp |
| OTU774 | k__Fungi;p__Basidiomycota;c__Agaricomycetes;o__Trechisporales;f__Hydnodontaceae;g__Trechispora;s__unidentified |
| OTU848 | k__Fungi;p__Basidiomycota;c__Agaricomycetes;o__Trechisporales;f__Hydnodontaceae;g__Trechispora;s__unidentified |
| OTU812 | k__Fungi;p__Basidiomycota;c__Agaricomycetes;o__Trechisporales;f__Trechisporales_fam_Incertae_sedis;g__Sistotremastrum;s__Sistotremastrum_guttuliferum |
| OTU651 | k__Fungi;p__Basidiomycota;c__Agaricomycetes;o__Trechisporales;f__unidentified;g__unidentified;s__unidentified |
| OTU954 | k__Fungi;p__Basidiomycota;c__Agaricomycetes;o__Trechisporales;f__unidentified;g__unidentified;s__unidentified |
| OTU253 | k__Fungi;p__Basidiomycota;c__Agaricomycetes;o__unidentified;f__unidentified;g__unidentified;s__unidentified |
| OTU958 | k__Fungi;p__Basidiomycota;c__Cystobasidiomycetes;o__Cyphobasidiales;f__unidentified;g__unidentified;s__unidentified |
| OTU499 | k__Fungi;p__Basidiomycota;c__Cystobasidiomycetes;o__Cystobasidiales;f__Cystobasidiaceae;g__Occultifur;s__unidentified |
| OTU213 | k__Fungi;p__Basidiomycota;c__Cystobasidiomycetes;o__Cystobasidiomycetes_ord_Incertae_sedis;f__Symmetrosporaceae;g__Symmetrospora;s__Symmetrospora_vermiculata |
| OTU1146 | k__Fungi;p__Basidiomycota;c__Exobasidiomycetes;o__Exobasidiales;f__Brachybasidiaceae;g__Meira;s__unidentified |
| OTU86 | k__Fungi;p__Basidiomycota;c__Exobasidiomycetes;o__Microstromatales;f__Quambalariaceae;g__Quambalaria;s__Quambalaria_fabacearum |
| OTU227 | k__Fungi;p__Basidiomycota;c__Exobasidiomycetes;o__Tilletiales;f__Tilletiaceae;g__Tilletia;s__Tilletia_barclayana |
| OTU1023 | k__Fungi;p__Basidiomycota;c__Geminibasidiomycetes;o__Geminibasidiales;f__Geminibasidiaceae;g__Basidioascus;s__Basidioascus_magus |
| OTU680 | k__Fungi;p__Basidiomycota;c__Geminibasidiomycetes;o__Geminibasidiales;f__Geminibasidiaceae;g__Geminibasidium;s__Geminibasidium_donsium |
| OTU376 | k__Fungi;p__Basidiomycota;c__Geminibasidiomycetes;o__Geminibasidiales;f__Geminibasidiaceae;g__Geminibasidium;s__Geminibasidium_hirsutum |
| OTU74 | k__Fungi;p__Basidiomycota;c__Geminibasidiomycetes;o__Geminibasidiales;f__Geminibasidiaceae;g__Geminibasidium;s__unidentified |
| OTU294 | k__Fungi;p__Basidiomycota;c__Geminibasidiomycetes;o__Geminibasidiales;f__Geminibasidiaceae;g__Geminibasidium;s__unidentified |
| OTU763 | k__Fungi;p__Basidiomycota;c__Malasseziomycetes;o__Malasseziales;f__Malasseziaceae;g__Malassezia;s__Malassezia_restricta |
| OTU581 | k__Fungi;p__Basidiomycota;c__Microbotryomycetes;o__Microbotryomycetes_ord_Incertae_sedis;f__Microbotryomycetes_fam_Incertae_sedis;g__Colacogloea;s__Colacogloea_philyla |
| OTU425 | k__Fungi;p__Basidiomycota;c__Microbotryomycetes;o__Sporidiobolales;f__Sporidiobolaceae;g__Rhodosporidiobolus;s__Rhodosporidiobolus_colostri |
| OTU546 | k__Fungi;p__Basidiomycota;c__Microbotryomycetes;o__Sporidiobolales;f__Sporidiobolaceae;g__Rhodotorula;s__Rhodotorula_diobovata |
| OTU439 | k__Fungi;p__Basidiomycota;c__Microbotryomycetes;o__Sporidiobolales;f__Sporidiobolaceae;g__Rhodotorula;s__unidentified |
| OTU619 | k__Fungi;p__Basidiomycota;c__Microbotryomycetes;o__Sporidiobolales;f__unidentified;g__unidentified;s__unidentified |
| OTU259 | k__Fungi;p__Basidiomycota;c__Microbotryomycetes;o__unidentified;f__unidentified;g__unidentified;s__unidentified |
| OTU1070 | k__Fungi;p__Basidiomycota;c__Tremellomycetes;o__Cystofilobasidiales;f__Cystofilobasidiaceae;g__Cystofilobasidium;s__Cystofilobasidium_capitatum |
| OTU1132 | k__Fungi;p__Basidiomycota;c__Tremellomycetes;o__Cystofilobasidiales;f__Mrakiaceae;g__Krasilnikovozyma;s__Krasilnikovozyma_huempii |
| OTU998 | k__Fungi;p__Basidiomycota;c__Tremellomycetes;o__Filobasidiales;f__Filobasidiaceae;g__Filobasidium;s__Filobasidium_sp |
| OTU635 | k__Fungi;p__Basidiomycota;c__Tremellomycetes;o__Filobasidiales;f__Filobasidiaceae;g__Heterocephalacria;s__Heterocephalacria_arrabidensis |
| OTU955 | k__Fungi;p__Basidiomycota;c__Tremellomycetes;o__Filobasidiales;f__Filobasidiaceae;g__Heterocephalacria;s__unidentified |
| OTU859 | k__Fungi;p__Basidiomycota;c__Tremellomycetes;o__Filobasidiales;f__Piskurozymaceae;g__Piskurozyma;s__Piskurozyma_cylindrica |
| OTU806 | k__Fungi;p__Basidiomycota;c__Tremellomycetes;o__Filobasidiales;f__Piskurozymaceae;g__Piskurozyma;s__unidentified |
| OTU1040 | k__Fungi;p__Basidiomycota;c__Tremellomycetes;o__Filobasidiales;f__Piskurozymaceae;g__Solicoccozyma;s__Solicoccozyma_terrea |
| OTU63 | k__Fungi;p__Basidiomycota;c__Tremellomycetes;o__Tremellales;f__Bulleraceae;g__Bullera;s__unidentified |
| OTU1314 | k__Fungi;p__Basidiomycota;c__Tremellomycetes;o__Tremellales;f__Bulleribasidiaceae;g__Hannaella;s__Hannaella_kunmingensis |
| OTU1010 | k__Fungi;p__Basidiomycota;c__Tremellomycetes;o__Tremellales;f__Bulleribasidiaceae;g__Hannaella;s__Hannaella_luteola |
| OTU1083 | k__Fungi;p__Basidiomycota;c__Tremellomycetes;o__Tremellales;f__Bulleribasidiaceae;g__Hannaella;s__Hannaella_oryzae |
| OTU1068 | k__Fungi;p__Basidiomycota;c__Tremellomycetes;o__Tremellales;f__Bulleribasidiaceae;g__Hannaella;s__Hannaella_pagnoccae |
| OTU1127 | k__Fungi;p__Basidiomycota;c__Tremellomycetes;o__Tremellales;f__Bulleribasidiaceae;g__Hannaella;s__Hannaella_surugaensis |
| OTU20 | k__Fungi;p__Basidiomycota;c__Tremellomycetes;o__Tremellales;f__Bulleribasidiaceae;g__Vishniacozyma;s__Vishniacozyma_foliicola |
| OTU1002 | k__Fungi;p__Basidiomycota;c__Tremellomycetes;o__Tremellales;f__Cryptococcaceae;g__Cryptococcus;s__Cryptococcus_longus |
| OTU383 | k__Fungi;p__Basidiomycota;c__Tremellomycetes;o__Tremellales;f__Cryptococcaceae;g__Kwoniella;s__Kwoniella_bestiolae |
| OTU192 | k__Fungi;p__Basidiomycota;c__Tremellomycetes;o__Tremellales;f__Cuniculitremaceae;g__Kockovaella;s__Kockovaella_litseae |
| OTU1003 | k__Fungi;p__Basidiomycota;c__Tremellomycetes;o__Tremellales;f__Rhynchogastremataceae;g__Papiliotrema;s__Papiliotrema_flavescens |
| OTU470 | k__Fungi;p__Basidiomycota;c__Tremellomycetes;o__Tremellales;f__Rhynchogastremataceae;g__Papiliotrema;s__Papiliotrema_nemorosus |
| OTU223 | k__Fungi;p__Basidiomycota;c__Tremellomycetes;o__Tremellales;f__Sirobasidiaceae;g__Fibulobasidium;s__Fibulobasidium_murrhardtense |
| OTU1115 | k__Fungi;p__Basidiomycota;c__Tremellomycetes;o__Tremellales;f__Tremellaceae;g__Cryptococcus;s__Cryptococcus_aspenensis |
| OTU961 | k__Fungi;p__Basidiomycota;c__Tremellomycetes;o__Tremellales;f__Tremellaceae;g__Cryptococcus;s__Cryptococcus_dimennae |
| OTU451 | k__Fungi;p__Basidiomycota;c__Tremellomycetes;o__Tremellales;f__Tremellaceae;g__Cryptococcus;s__unidentified |
| OTU1201 | k__Fungi;p__Basidiomycota;c__Tremellomycetes;o__Tremellales;f__Tremellaceae;g__Tremella;s__unidentified |
| OTU399 | k__Fungi;p__Basidiomycota;c__Tremellomycetes;o__Tremellales;f__Tremellales_fam_Incertae_sedis;g__Mingxiaea;s__Mingxiaea_panici |
| OTU394 | k__Fungi;p__Basidiomycota;c__Tremellomycetes;o__Tremellales;f__Trimorphomycetaceae;g__Saitozyma;s__Saitozyma_podzolica |
| OTU746 | k__Fungi;p__Basidiomycota;c__Tremellomycetes;o__Tremellales;f__Trimorphomycetaceae;g__Saitozyma;s__unidentified |
| OTU275 | k__Fungi;p__Basidiomycota;c__Tremellomycetes;o__Tremellales;f__unidentified;g__unidentified;s__unidentified |
| OTU357 | k__Fungi;p__Basidiomycota;c__Tremellomycetes;o__Tremellales;f__unidentified;g__unidentified;s__unidentified |
| OTU489 | k__Fungi;p__Basidiomycota;c__Tremellomycetes;o__Tremellales;f__unidentified;g__unidentified;s__unidentified |
| OTU637 | k__Fungi;p__Basidiomycota;c__Tremellomycetes;o__Trichosporonales;f__Tetragoniomycetaceae;g__Tetragoniomyces;s__Tetragoniomyces_uliginosus |
| OTU996 | k__Fungi;p__Basidiomycota;c__Tremellomycetes;o__Trichosporonales;f__Trichosporonaceae;g__Apiotrichum;s__Apiotrichum_brassicae |
| OTU1046 | k__Fungi;p__Basidiomycota;c__Tremellomycetes;o__Trichosporonales;f__Trichosporonaceae;g__Apiotrichum;s__Apiotrichum_dehoogii |
| OTU1071 | k__Fungi;p__Basidiomycota;c__Tremellomycetes;o__Trichosporonales;f__Trichosporonaceae;g__Apiotrichum;s__Apiotrichum_laibachii |
| OTU986 | k__Fungi;p__Basidiomycota;c__Tremellomycetes;o__Trichosporonales;f__Trichosporonaceae;g__Apiotrichum;s__Apiotrichum_lignicola |
| OTU760 | k__Fungi;p__Basidiomycota;c__Tremellomycetes;o__Trichosporonales;f__Trichosporonaceae;g__Apiotrichum;s__Apiotrichum_porosum |
| OTU292 | k__Fungi;p__Basidiomycota;c__Tremellomycetes;o__Trichosporonales;f__Trichosporonaceae;g__Cutaneotrichosporon;s__Cutaneotrichosporon_moniliiforme |
| OTU759 | k__Fungi;p__Basidiomycota;c__Tremellomycetes;o__Trichosporonales;f__Trichosporonaceae;g__Trichosporon;s__Trichosporon_asahii |
| OTU769 | k__Fungi;p__Basidiomycota;c__Tremellomycetes;o__Trichosporonales;f__Trichosporonaceae;g__Vanrija;s__Vanrija_humicola |
| OTU46 | k__Fungi;p__Basidiomycota;c__Tremellomycetes;o__unidentified;f__unidentified;g__unidentified;s__unidentified |
| OTU649 | k__Fungi;p__Basidiomycota;c__Tremellomycetes;o__unidentified;f__unidentified;g__unidentified;s__unidentified |
| OTU687 | k__Fungi;p__Basidiomycota;c__Tritirachiomycetes;o__Tritirachiales;f__Tritirachiaceae;g__Tritirachium;s__Tritirachium_cinnamomeum |
| OTU137 | k__Fungi;p__Basidiomycota;c__Wallemiomycetes;o__Wallemiales;f__Wallemiaceae;g__Wallemia;s__Wallemia_tropicalis |

**Blastocladiomycota**

| **OUT number** | **Taxonomy** |
| --- | --- |
| OTU755 | k__Fungi;p__Blastocladiomycota;c__Blastocladiomycetes;o__Blastocladiales;f__Blastocladiaceae;g__Allomyces;s__Allomyces_arbusculus |
| OTU212 | k__Fungi;p__Blastocladiomycota;c__Blastocladiomycetes;o__Blastocladiales;f__Catenariaceae;g__Catenaria;s__Catenaria_anguillulae |

**Chytridiomycota**

| **OUT number** | **Taxonomy** |
| --- | --- |
| OTU185 | k__Fungi;p__Chytridiomycota;c__Chytridiomycetes;o__Chytridiales;f__Chytridiaceae;g__Chytridium;s__Chytridium_olla |
| OTU623 | k__Fungi;p__Chytridiomycota;c__Chytridiomycetes;o__Chytridiales;f__Chytridiaceae;g__Dendrochytridium;s__Dendrochytridium_crassum |
| OTU951 | k__Fungi;p__Chytridiomycota;c__Chytridiomycetes;o__Chytridiales;f__Chytridiaceae;g__Phlyctochytrium;s__Phlyctochytrium_africanum |
| OTU51 | k__Fungi;p__Chytridiomycota;c__Chytridiomycetes;o__Chytridiales;f__Chytridiaceae;g__Phlyctochytrium;s__Phlyctochytrium_planicorne |
| OTU1270 | k__Fungi;p__Chytridiomycota;c__Chytridiomycetes;o__Chytridiales;f__unidentified;g__unidentified;s__unidentified |
| OTU126 | k__Fungi;p__Chytridiomycota;c__Chytridiomycetes;o__unidentified;f__unidentified;g__unidentified;s__unidentified |
| OTU1035 | k__Fungi;p__Chytridiomycota;c__Rhizophlyctidomycetes;o__Rhizophlyctidales;f__Rhizophlyctidaceae;g__Rhizophlyctis;s__Rhizophlyctis_rosea |
| OTU1192 | k__Fungi;p__Chytridiomycota;c__Rhizophlyctidomycetes;o__Rhizophlyctidales;f__Sonoraphlyctidaceae;g__Sonoraphlyctis;s__Sonoraphlyctis_ranzonii |
| OTU615 | k__Fungi;p__Chytridiomycota;c__Rhizophydiomycetes;o__Rhizophydiales;f__Alphamycetaceae;g__Alphamyces;s__Alphamyces_chaetifer |
| OTU1061 | k__Fungi;p__Chytridiomycota;c__Rhizophydiomycetes;o__Rhizophydiales;f__Alphamycetaceae;g__Betamyces;s__unidentified |
| OTU68 | k__Fungi;p__Chytridiomycota;c__Rhizophydiomycetes;o__Rhizophydiales;f__Gorgonomycetaceae;g__Gorgonomyces;s__Gorgonomyces_haynaldii |
| OTU230 | k__Fungi;p__Chytridiomycota;c__Rhizophydiomycetes;o__Rhizophydiales;f__Kappamycetaceae;g__Kappamyces;s__Kappamyces_laurelensis |
| OTU1196 | k__Fungi;p__Chytridiomycota;c__Rhizophydiomycetes;o__Rhizophydiales;f__Rhizophydiaceae;g__Rhizophydium;s__unidentified |
| OTU545 | k__Fungi;p__Chytridiomycota;c__Rhizophydiomycetes;o__Rhizophydiales;f__Rhizophydiales_fam_Incertae_sedis;g__Coralloidiomyces;s__Coralloidiomyces_digitatus |
| OTU290 | k__Fungi;p__Chytridiomycota;c__Rhizophydiomycetes;o__Rhizophydiales;f__unidentified;g__unidentified;s__unidentified |
| OTU642 | k__Fungi;p__Chytridiomycota;c__Spizellomycetes;o__Spizellomycetales;f__Powellomycetaceae;g__Powellomyces;s__unidentified |
| OTU734 | k__Fungi;p__Chytridiomycota;c__Spizellomycetes;o__Spizellomycetales;f__Spizellomycetaceae;g__Kochiomyces;s__unidentified |
| OTU110 | k__Fungi;p__Chytridiomycota;c__Spizellomycetes;o__Spizellomycetales;f__Spizellomycetaceae;g__Spizellomyces;s__Spizellomyces_kniepii |
| OTU1212 | k__Fungi;p__Chytridiomycota;c__Spizellomycetes;o__Spizellomycetales;f__Spizellomycetaceae;g__Spizellomyces;s__Spizellomyces_plurigibbosus |
| OTU182 | k__Fungi;p__Chytridiomycota;c__Spizellomycetes;o__Spizellomycetales;f__Spizellomycetaceae;g__Spizellomyces;s__Spizellomyces_pseudodichotomus |
| OTU913 | k__Fungi;p__Chytridiomycota;c__Spizellomycetes;o__Spizellomycetales;f__Spizellomycetaceae;g__Triparticalcar;s__Triparticalcar_equi |
| OTU330 | k__Fungi;p__Chytridiomycota;c__Spizellomycetes;o__Spizellomycetales;f__unidentified;g__unidentified;s__unidentified |
| OTU1245 | k__Fungi;p__Chytridiomycota;c__Spizellomycetes;o__Spizellomycetales;f__unidentified;g__unidentified;s__unidentified |
| OTU908 | k__Fungi;p__Chytridiomycota;c__Synchytriomycetes;o__Synchytriales;f__Synchytriaceae;g__Synchytrium;s__Synchytrium_endobioticum |
| OTU37 | k__Fungi;p__Chytridiomycota;c__Synchytriomycetes;o__Synchytriales;f__Synchytriaceae;g__Synchytrium;s__Synchytrium_papillatum |
| OTU80 | k__Fungi;p__Chytridiomycota;c__unidentified;o__unidentified;f__unidentified;g__unidentified;s__unidentified |
| OTU145 | k__Fungi;p__Chytridiomycota;c__unidentified;o__unidentified;f__unidentified;g__unidentified;s__unidentified |
| OTU255 | k__Fungi;p__Chytridiomycota;c__unidentified;o__unidentified;f__unidentified;g__unidentified;s__unidentified |

**Glomeromycota**

| **OUT number** | **Taxonomy** |
| --- | --- |
| OTU393 | k__Fungi;p__Glomeromycota;c__Archaeosporomycetes;o__Archaeosporales;f__Archaeosporaceae;g__Archaeospora;s__unidentified |
| OTU1130 | k__Fungi;p__Glomeromycota;c__Glomeromycetes;o__Diversisporales;f__Acaulosporaceae;g__Acaulospora;s__Acaulospora_tortuosa |
| OTU129 | k__Fungi;p__Glomeromycota;c__Glomeromycetes;o__Gigasporales;f__Gigasporaceae;g__Cetraspora;s__Cetraspora_nodosa |
| OTU1106 | k__Fungi;p__Glomeromycota;c__Glomeromycetes;o__Glomerales;f__Glomeraceae;g__Glomus;s__unidentified |
| OTU553 | k__Fungi;p__Glomeromycota;c__Glomeromycetes;o__Glomerales;f__Glomeraceae;g__Septoglomus;s__Septoglomus_constrictum |
| OTU303 | k__Fungi;p__Glomeromycota;c__Glomeromycetes;o__Glomerales;f__Glomeraceae;g__unidentified;s__unidentified |
| OTU847 | k__Fungi;p__Glomeromycota;c__Glomeromycetes;o__Glomerales;f__unidentified;g__unidentified;s__unidentified |
| OTU1112 | k__Fungi;p__Glomeromycota;c__unidentified;o__unidentified;f__unidentified;g__unidentified;s__unidentified |

**Kickxellomycota**

| **OUT number** | **Taxonomy** |
| --- | --- |
| OTU217 | k__Fungi;p__Kickxellomycota;c__Kickxellomycetes;o__Kickxellales;f__unidentified;g__unidentified;s__unidentified |

**Mortierellomycota**

| **OUT number** | **Taxonomy** |
| --- | --- |
| OTU586 | k__Fungi;p__Mortierellomycota;c__Mortierellomycetes;o__Mortierellales;f__Mortierellaceae;g__Mortierella;s__Mortierella_alpina |
| OTU437 | k__Fungi;p__Mortierellomycota;c__Mortierellomycetes;o__Mortierellales;f__Mortierellaceae;g__Mortierella;s__Mortierella_amoeboidea |
| OTU963 | k__Fungi;p__Mortierellomycota;c__Mortierellomycetes;o__Mortierellales;f__Mortierellaceae;g__Mortierella;s__Mortierella_beljakovae |
| OTU87 | k__Fungi;p__Mortierellomycota;c__Mortierellomycetes;o__Mortierellales;f__Mortierellaceae;g__Mortierella;s__Mortierella_camargensis |
| OTU713 | k__Fungi;p__Mortierellomycota;c__Mortierellomycetes;o__Mortierellales;f__Mortierellaceae;g__Mortierella;s__Mortierella_chienii |
| OTU1088 | k__Fungi;p__Mortierellomycota;c__Mortierellomycetes;o__Mortierellales;f__Mortierellaceae;g__Mortierella;s__Mortierella_dichotoma |
| OTU176 | k__Fungi;p__Mortierellomycota;c__Mortierellomycetes;o__Mortierellales;f__Mortierellaceae;g__Mortierella;s__Mortierella_elongata |
| OTU1076 | k__Fungi;p__Mortierellomycota;c__Mortierellomycetes;o__Mortierellales;f__Mortierellaceae;g__Mortierella;s__Mortierella_exigua |
| OTU423 | k__Fungi;p__Mortierellomycota;c__Mortierellomycetes;o__Mortierellales;f__Mortierellaceae;g__Mortierella;s__Mortierella_fimbricystis |
| OTU89 | k__Fungi;p__Mortierellomycota;c__Mortierellomycetes;o__Mortierellales;f__Mortierellaceae;g__Mortierella;s__Mortierella_globulifera |
| OTU189 | k__Fungi;p__Mortierellomycota;c__Mortierellomycetes;o__Mortierellales;f__Mortierellaceae;g__Mortierella;s__Mortierella_horticola |
| OTU142 | k__Fungi;p__Mortierellomycota;c__Mortierellomycetes;o__Mortierellales;f__Mortierellaceae;g__Mortierella;s__Mortierella_humilis |
| OTU242 | k__Fungi;p__Mortierellomycota;c__Mortierellomycetes;o__Mortierellales;f__Mortierellaceae;g__Mortierella;s__Mortierella_hypsicladia |
| OTU910 | k__Fungi;p__Mortierellomycota;c__Mortierellomycetes;o__Mortierellales;f__Mortierellaceae;g__Mortierella;s__Mortierella_lignicola |
| OTU923 | k__Fungi;p__Mortierellomycota;c__Mortierellomycetes;o__Mortierellales;f__Mortierellaceae;g__Mortierella;s__Mortierella_nantahalensis |
| OTU1230 | k__Fungi;p__Mortierellomycota;c__Mortierellomycetes;o__Mortierellales;f__Mortierellaceae;g__Mortierella;s__Mortierella_sarnyensis |
| OTU194 | k__Fungi;p__Mortierellomycota;c__Mortierellomycetes;o__Mortierellales;f__Mortierellaceae;g__Mortierella;s__Mortierella_umbellata |
| OTU320 | k__Fungi;p__Mortierellomycota;c__Mortierellomycetes;o__Mortierellales;f__Mortierellaceae;g__Mortierella;s__unidentified |
| OTU458 | k__Fungi;p__Mortierellomycota;c__Mortierellomycetes;o__Mortierellales;f__unidentified;g__unidentified;s__unidentified |

**Mucoromycota**

| **OUT number** | **Taxonomy** |
| --- | --- |
| OTU569 | k__Fungi;p__Mucoromycota;c__Endogonomycetes;o__Endogonales;f__unidentified;g__unidentified;s__unidentified |
| OTU710 | k__Fungi;p__Mucoromycota;c__Endogonomycetes;o__GS20;f__unidentified;g__unidentified;s__unidentified |
| OTU896 | k__Fungi;p__Mucoromycota;c__Mucoromycetes;o__Mucorales;f__Backusellaceae;g__Backusella;s__Backusella_circina |
| OTU736 | k__Fungi;p__Mucoromycota;c__Mucoromycetes;o__Mucorales;f__Cunninghamellaceae;g__Cunninghamella;s__Cunninghamella_binariae |
| OTU1276 | k__Fungi;p__Mucoromycota;c__Mucoromycetes;o__Mucorales;f__Cunninghamellaceae;g__Cunninghamella;s__Cunninghamella_elegans |
| OTU435 | k__Fungi;p__Mucoromycota;c__Mucoromycetes;o__Mucorales;f__Cunninghamellaceae;g__Gongronella;s__Gongronella_butleri |
| OTU249 | k__Fungi;p__Mucoromycota;c__Mucoromycetes;o__Mucorales;f__Lichtheimiaceae;g__Thamnostylum;s__Thamnostylum_piriforme |
| OTU568 | k__Fungi;p__Mucoromycota;c__Mucoromycetes;o__Mucorales;f__Mucoraceae;g__Isomucor;s__Isomucor_trufemiae |
| OTU805 | k__Fungi;p__Mucoromycota;c__Mucoromycetes;o__Mucorales;f__Mucoraceae;g__Mucor;s__Mucor_abundans |
| OTU931 | k__Fungi;p__Mucoromycota;c__Mucoromycetes;o__Mucorales;f__Mucoraceae;g__Mucor;s__Mucor_bainieri |
| OTU1214 | k__Fungi;p__Mucoromycota;c__Mucoromycetes;o__Mucorales;f__Mucoraceae;g__Mucor;s__Mucor_minutus |
| OTU873 | k__Fungi;p__Mucoromycota;c__Mucoromycetes;o__Mucorales;f__Mucoraceae;g__Mucor;s__Mucor_moelleri |
| OTU756 | k__Fungi;p__Mucoromycota;c__Mucoromycetes;o__Mucorales;f__Mucoraceae;g__Mucor;s__Mucor_racemosus |
| OTU700 | k__Fungi;p__Mucoromycota;c__Mucoromycetes;o__Mucorales;f__Mucoraceae;g__Mucor;s__unidentified |
| OTU98 | k__Fungi;p__Mucoromycota;c__Mucoromycetes;o__Mucorales;f__Mucoraceae;g__unidentified;s__unidentified |
| OTU100 | k__Fungi;p__Mucoromycota;c__Mucoromycetes;o__Mucorales;f__Mucoraceae;g__unidentified;s__unidentified |
| OTU438 | k__Fungi;p__Mucoromycota;c__Mucoromycetes;o__Mucorales;f__Rhizopodaceae;g__Rhizopus;s__Rhizopus_arrhizus |
| OTU347 | k__Fungi;p__Mucoromycota;c__Mucoromycotina_cls_Incertae_sedis;o__Mucoromycotina_ord_Incertae_sedis;f__Mucoromycotina_fam_Incertae_sedis;g__Bifiguratus;s__Bifiguratus_adelaidae |
| OTU233 | k__Fungi;p__Mucoromycota;c__Umbelopsidomycetes;o__Umbelopsidales;f__Umbelopsidaceae;g__Umbelopsis;s__Umbelopsis_angularis |
| OTU338 | k__Fungi;p__Mucoromycota;c__Umbelopsidomycetes;o__Umbelopsidales;f__Umbelopsidaceae;g__Umbelopsis;s__Umbelopsis_dimorpha |
| OTU863 | k__Fungi;p__Mucoromycota;c__Umbelopsidomycetes;o__Umbelopsidales;f__Umbelopsidaceae;g__Umbelopsis;s__Umbelopsis_isabellina |
| OTU703 | k__Fungi;p__Mucoromycota;c__Umbelopsidomycetes;o__Umbelopsidales;f__Umbelopsidaceae;g__Umbelopsis;s__Umbelopsis_ramanniana |
| OTU622 | k__Fungi;p__Mucoromycota;c__Umbelopsidomycetes;o__Umbelopsidales;f__Umbelopsidaceae;g__Umbelopsis;s__unidentified |

**Rozellomycota**

| **OUT number** | **Taxonomy** |
| --- | --- |
| OTU601 | k__Fungi;p__Rozellomycota;c__Rozellomycotina_cls_Incertae_sedis;o__GS04;f__unidentified;g__unidentified;s__unidentified |
| OTU814 | k__Fungi;p__Rozellomycota;c__Rozellomycotina_cls_Incertae_sedis;o__GS04;f__unidentified;g__unidentified;s__unidentified |
| OTU944 | k__Fungi;p__Rozellomycota;c__Rozellomycotina_cls_Incertae_sedis;o__GS05;f__unidentified;g__unidentified;s__unidentified |
| OTU1016 | k__Fungi;p__Rozellomycota;c__Rozellomycotina_cls_Incertae_sedis;o__GS05;f__unidentified;g__unidentified;s__unidentified |
| OTU83 | k__Fungi;p__Rozellomycota;c__Rozellomycotina_cls_Incertae_sedis;o__GS07;f__unidentified;g__unidentified;s__unidentified |
| OTU482 | k__Fungi;p__Rozellomycota;c__Rozellomycotina_cls_Incertae_sedis;o__GS08;f__unidentified;g__unidentified;s__unidentified |
| OTU1335 | k__Fungi;p__Rozellomycota;c__Rozellomycotina_cls_Incertae_sedis;o__GS09;f__unidentified;g__unidentified;s__unidentified |
| OTU47 | k__Fungi;p__Rozellomycota;c__Rozellomycotina_cls_Incertae_sedis;o__GS11;f__unidentified;g__unidentified;s__unidentified |
| OTU116 | k__Fungi;p__Rozellomycota;c__Rozellomycotina_cls_Incertae_sedis;o__GS11;f__unidentified;g__unidentified;s__unidentified |
| OTU261 | k__Fungi;p__Rozellomycota;c__Rozellomycotina_cls_Incertae_sedis;o__GS11;f__unidentified;g__unidentified;s__unidentified |
| OTU428 | k__Fungi;p__Rozellomycota;c__Rozellomycotina_cls_Incertae_sedis;o__GS11;f__unidentified;g__unidentified;s__unidentified |
| OTU662 | k__Fungi;p__Rozellomycota;c__Rozellomycotina_cls_Incertae_sedis;o__GS11;f__unidentified;g__unidentified;s__unidentified |
| OTU270 | k__Fungi;p__Rozellomycota;c__unidentified;o__unidentified;f__unidentified;g__unidentified;s__unidentified |
| OTU594 | k__Fungi;p__Rozellomycota;c__unidentified;o__unidentified;f__unidentified;g__unidentified;s__unidentified |

**Zoopagomycota**

| **OUT number** | **Taxonomy** |
| --- | --- |
| OTU269 | k__Fungi;p__Zoopagomycota;c__Zoopagomycetes;o__Zoopagales;f__Piptocephalidaceae;g__Syncephalis;s__unidentified |
| OTU278 | k__Fungi;p__Zoopagomycota;c__Zoopagomycetes;o__Zoopagales;f__Piptocephalidaceae;g__Syncephalis;s__unidentified |
